# Harmful Microalgae Exhibit Broad Environmental Adaptability in High‐Salinity Area Across the Dafengjiang River Estuary

**DOI:** 10.1002/ece3.70455

**Published:** 2024-10-23

**Authors:** Jiongqing Huang, Huaxian Zhao, WeiJun Wang, Xinyi Qin, Pengbin Wang, Qinghua Hou, Qingxiang Chen, Gonglingxia Jiang, Ke Dong, Tao Jiang, Yang Pu, Nan Li

**Affiliations:** ^1^ School of Agriculture Ludong University Yantai China; ^2^ Laboratory for Coastal Ocean Variation and Disaster Prediction, College of Ocean and Meteorology Guangdong Ocean University Zhanjiang China; ^3^ Key Laboratory of Climate, Resources and Environment in Continental Shelf Sea and Deep Sea of Department of Education of Guangdong Province Guangdong Ocean University Zhanjiang China; ^4^ Key Laboratory of Environment Change and Resources Use in Beibu Gulf, Ministry of Education Nanning Normal University Nanning China; ^5^ Key Laboratory of Marine Ecosystem Dynamics Second Institute of Oceanography, Ministry of Natural Resources Hangzhou China; ^6^ Department of Biological Sciences Kyonggi University Suwon South Korea; ^7^ School of Ocean Yantai University Yantai China

**Keywords:** 18S rRNA gene, community‐level change points, harmful algal blooms, harmful microalgae, subtropical estuary, TITAN

## Abstract

Harmful algal blooms (HABs) often occur in estuaries due to their unique environmental heterogeneity, posing significant environmental and human health risks. However, there is limited understanding of the community composition and community‐level change points (thresholds) of harmful microalgae in subtropical estuaries. This study explored harmful microalgae community structure and thresholds in the Dafengjiang River estuary using a metabarcoding approach. The results revealed 63 harmful microalgae species, and major species included *Guinardia flaccida*, *Prorocentrum cordatum*, *Thalassiosira punctigera*, *Pseudo‐nitzschia galaxiae* and *T. gravida*. Nonparametric change‐point analysis and threshold indicator taxa analysis (TITAN) showed threshold responses of harmful microalgae community structure to ammonium (57.5–60 μg·L^−1^), total phosphorus (27.8–28.5 μg·L^−1^) and dissolved inorganic phosphorus (14.5–28 μg·L^−1^) along the salinity gradient. Wider environmental thresholds were also found in hypersaline areas. Additionally, *Pyrodinium bahamense*, *Pfiesteria piscicida*, *Skeletonema tropicum* and *T. punctigera* were sensitive to environmental changes and thus could be used as bioindicators. Overall, our study unveiled diverse abrupt transitions of harmful microalgal communities, providing a risk assessment for human health and ecological safety in subtropical estuary ecosystems.

## Graphical abstract

The alpha diversity and beta diversity of the harmful microalgae community exhibited significant variations in salinity and seasonality (*p* < 0.05). A wider range of environmental thresholds in high‐salinity samples than those in other samples of the harmful microalgae community. Ammonium, total phosphorus, and dissolved inorganic phosphorus criteria were 57.5–60, 27.8–28.5 and 14.5–28 μg·L^−1^, respectively. *Pyrodinium bahamense*, *Pfiesteria piscicida*, *Skeletonema tropicum* and *T. punctigera* were the most important bioindicators of environmental change in the different salinity areas.

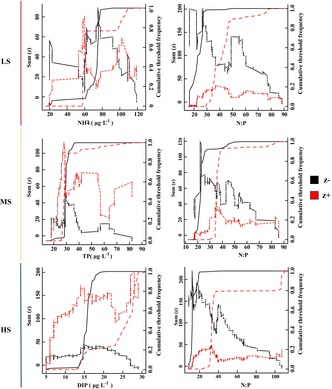

## 1 Introduction

Harmful algal blooms (HABs), which involve the excessive propagation of noxious or toxic phytoplankton, frequently occur in eutrophic coastal waters worldwide (Griffith and Gobler 2020; Astuya et al. 2015; Xiao et al. 2019). Various species induce HABs, such as *Pyrodinium bahamense*, which produces paralytic shellfish toxins (PSTs) resulting in mortality events in finfish, marine mammals and seabirds (Emslie et al. 1996; Band‐Schmidt et al. 2011, 2019; Amaya et al. 2014; Núñez‐Vázquez et al. 2007, 2022). Also, *Karlodinium veneficum* synthesizes a unique suite of polyketide toxins known as karlotoxins, which disrupt the membrane permeability of vertebrate cells, causing fish kills (Liu et al. 2020b; Deeds et al. 2015). Thus, HABs exert substantial perturbations to marine ecosystems, deleterious impacts on fisheries and human health.

Estuaries possess complex ecological processes and dynamic environmental conditions, featuring intricate currents, substantial nutrient inputs and fluctuating salinity gradients (Ge et al. 2020; Paerl 2006). Human activities and climate change can lead to severe eutrophication in estuaries (Statham 2012), rendering estuarine coastal regions susceptible to frequent and harmful algal blooms (Paerl, Otten, and Kudela 2018). In recent decades, HABs have become increasingly common in China's estuaries. For instance, the Yangtze River Estuary has been dominated by diatom species such as *Pseudo‐nitzschia*, *Skeletonema* and *Thalassiosira* (Cui et al. 2021). These diatom blooms are frequently observed along various coastal regions of China, including the Pearl River Estuary, Jiaozhou Bay, and the northern part of Bohai Bay (Wang et al. 2023; Yu, Tang, and Gobler 2023). He et al. (2023) also found that the Beibu Gulf was predominantly characterized by the presence of HAB genera *Karlodinium*, *Prorocentrum*, *Chaetoceros* and *Gymnodinium*. However, the understanding of the diversity within the harmful microalgae community in subtropical estuaries remains limited.

Previous studies indicated that environmental factors like temperature, phosphorus and nitrates play a significant role in shaping the structure of harmful microalgae communities. For example, Han et al. (2020) found that the temperature was the limiting factor for the growth of *G. catenatum* and proposed that the most optimal growth rate for the microalgae occurs at 20°C in the South Sea of Korea. In the South China Sea, Wang et al. (2022) reported that a total of 62 HAB taxa, including 26 diatoms, 33 dinoflagellates and 3 taxa belonging to Ochrophyta, were significantly positively correlated with nutrient levels. In addition, *Pr. cordatum* had the highest growth rate and became the dominant species at an N:P ratio near or above the Redfield proportions (16:1 or 25:1) (Glibert, Burkholder, and Kana 2012). Recent studies have increasingly emphasized the importance of community‐level change points, which reflect changes in taxa distributions along an environmental gradient (Zhang et al. 2016; Cao et al. 2016). Zhang et al. (2016), through Threshold Indicator Taxa Analysis (TITAN), demonstrate that distinct community change points of total nitrogen (TN) in the sensitive diatoms occurred at 0.96 × 10^3^–1.63 × 10^3^ μg·L^−1^ in connected lakes and 0.52 × 10^3^–0.63 × 10^3^ μg·L^−1^ in isolated lakes. In a shallow eutrophic Chinese lake, the thresholds in total phosphorus (TP) for phytoplankton community were 1.32× 10^5^ and 1.52 × 10^5^ μg·L^−1^ (Cao et al. 2016). However, the community‐level change points of the harmful microalgae across the intricate environmental gradients in the subtropical estuary remain poorly understood.

Dafengjiang River estuary, located on the south coast of Guangxi province, China, is a shallow subtropical estuary linking Qinzhou Bay with the Beibu Gulf (Yang et al. 2018; Lu et al. 2020). The estuary, exhibiting a triangular morphology, forms a main stream debouching into the open Beibu Gulf (Yang et al. 2018). The estuary experiences significant human impact, particularly from aquaculture, which leads to excessive nutrient accumulation, eutrophication and HABs (Yang et al. 2018; Tang et al. 2003; Chai et al. 2006; Zhu et al. 2011; Zhao et al. 2023). To explore the characteristics of the harmful microalgae community and its community‐level change points, this study used high‐throughput sequencing of the 18S rRNA gene to analyse the harmful microalgae community from the seawater samples at the Dafengjiang River estuary in the Beibu Gulf. The objectives of this study are to (i) investigate harmful microalgae species composition and distribution, (ii) explore key divers and distinct community change points of harmful microalgae communities and (iii) identify important harmful microalgae species indicators in the subtropical estuary. Our findings provide novel perspectives on the mechanisms underlying HABs and the environmental monitoring of subtropical estuaries.

## 2 Materials and Methods

### 2.1 Sampling Stations and Environmental Factors Analysis

**FIGURE 1:**
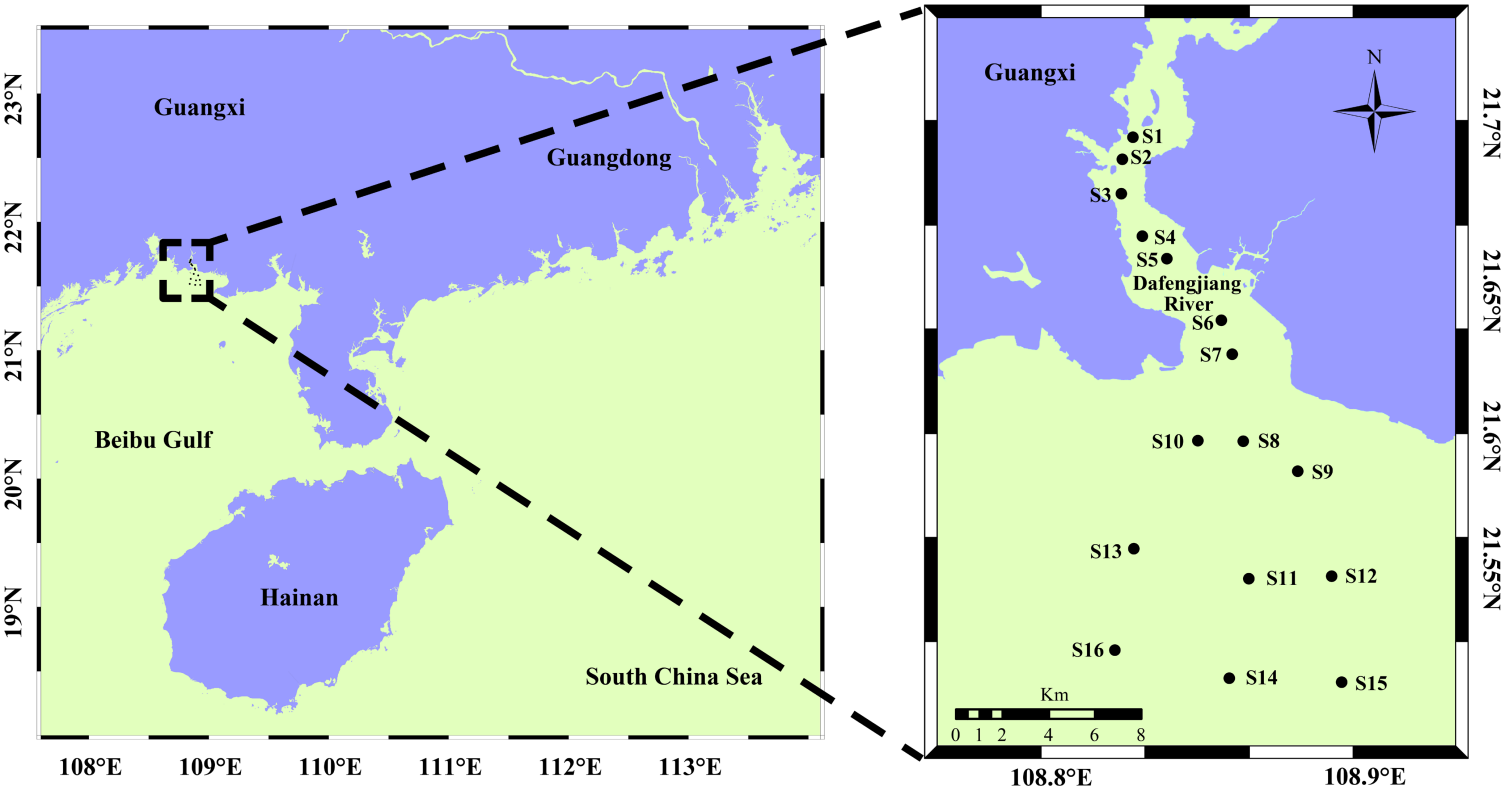
Location of the sampling sites in the Dafengjiang River estuary.

In 2018 and 2020, surface seawater samples were collected from 16 sites in the Dafengjiang River estuary, China, at a depth of 0.5 m (Figure 1). Sampling was conducted across four seasons: spring (March), summer (July), autumn (September) and winter (December), with five replicates per site. However, samples were not available at the S14 site in the spring of 2018, and no samples were collected at the S8 site in 2018, resulting in the collection of a total of 615 samples. At each site, 5 L of seawater were gathered using a Niskin bottle. Water quality parameters, including pH, temperature, salinity and dissolved oxygen (DO), were measured on‐site with a portable metre (556 MPS; YSI, USA). To analyse nutrients and Chl‐*a*, 500 mL of seawater was filtered shipboard through a 0.45‐μm filter (Millipore Corporation, Billerica, MA, USA). The filters were then stored at −80°C and transported to the laboratory in an icebox. The Chl‐*a* concentration was determined by spectrophotometry (American Public Health Association 1926). The concentrations of TP, phosphate–phosphorus (PO_4_^3−^‐P), TN, nitrite–nitrogen (NO_2_^−^‐N), nitrate–nitrogen (NO_3_^−^‐N) and ammonium–nitrogen (NH_4_^+^‐N) were measured via a continuous flow analyser (Seal‐AA3, Norderstedt, Germany). Total organic carbon (TOC) was measured using a total organic carbon analyser (TOC‐VCPH, Shimadzu, Japan). Seawater chemical oxygen demand (COD) was evaluated by alkalescent permanganate titration. The dissolved inorganic nitrogen (DIN) was composed of NO_2_^−^‐N, NO_3_^−^‐N, and NH_4_^+^‐N, whereas the dissolved inorganic phosphorus (DIP) was measured as PO_4_^3−^‐P (Lai et al. 2014). According to the salinity gradient, harmful microalgae community data were spatially divided into three groups: low‐salinity (LS) (S1‐S4), medium‐salinity (MS) (S5‐S9) and high‐salinity (HS) areas (S10‐S15), ranging from 22.30 ± 2.73, 26.56 ± 2.58 and 29.99 ± 2.23 ppt, respectively (Figure S1).

### 2.2 DNA Extraction and PCR

Three litres of seawater from each sample were filtered through a polycarbonate membrane with a pore size of 0.22 μm (Millipore Corporation, Billerica, MA, USA). The polycarbonate membranes were preserved at −80°C for subsequent DNA extraction. Environmental DNA was extracted using the DNeasy PowerWater Kit (QIAGEN, Germantown, MD, USA) following the manufacturer's protocols and stored at −80°C. Amplification of the V4 region (~380 bp) of 18S rRNA was conducted using the TAReuk454FWD1 (5′‐CCAGCASCYGCGGTAATTCC‐3′) and TAReukREV3 (5′‐ACTTTCGTTCTTGATYRA‐3′) primers (Logares et al. 2012). The PCR reaction included 1× Taq PCR Mastermix (TIANGEN, Beijing, China), 0.5 μM of each forward and reverse primer, and approximately 2.5 ng of template DNA, with double‐distilled H_2_O added to a final volume of 20 μL. PCR cycling parameters consisted of an initial denaturation at 98°C for 2 min, followed by 10 cycles of denaturation at 98°C for 10 s, annealing at 53°C for 30 s, and extension at 72°C for 30 s, then 15 cycles of denaturation at 98°C for 10 s, annealing at 48°C for 30 s and extension at 72°C for 30 s, with a final extension at 72°C for 2 min. Subsequently, PCR products were processed for sequencing using a TruSeq DNA kit (Illumina, United States).

### 2.3 High‐Throughput Sequencing and Taxonomic Annotation

A purified library was constructed for each sample following Illumina library preparation protocols. All purified libraries were sent to MajorBio Biotech (Shanghai, China) for paired‐end sequencing on an Illumina MiSeq platform. Primer mismatches and reads < 275 bp, low‐quality reads (with quality scores < 30), and barcode sequences were removed using Qiime2 (Caporaso et al. 2010). The chimeric sequences were detected and eliminated using UCHIME method based on de novo (Edgar et al. 2011). Dereplication step was performed, then operational taxonomic units (OTUs) with a 97% sequence identity were subjected to de novo clustering utilizing Qiime2. Representative sequences of each OTU were subsequently annotated using the classify consensus‐vsearch method, referencing the Protist Ribosomal Reference (PR^2^) database version 5.0.0 (https://doi.org/10.5281/zenodo.7805244). Only sequences belonging to microalgae were retained. A taxon was annotated as a harmful microalgae species if it was categorized as a harmful microalga in the online version of the IOC‐UNESCO Taxonomic Reference List of Harmful Microalgae (Moestrup 2009) or had been reported as an HAB or bloom species in previous studies (Wang et al. 2022; Huang et al. 2023; Chen, Cui, and Xu 2021; Gu et al. 2022; Cui et al. 2021). The raw sequences generated in this study were submitted to GenBank under the accession numbers PRJNA1044415 and PRJNA866330.

### 2.4 Statistical Analysis

To simplify the OTU taxonomy and reduce the complexity of subsequent analyses, OTUs with only 1 or fewer than 5 occurrences were filtered out. All statistical analyses were mainly performed using R platform with the ‘vegan’, ‘randomForest’, ‘ggplot2’ and ‘psych’ packages. Differences in environmental parameters among the sampling sites were examined by one‐way analysis of variance (ANOVA). The Shannon index was employed to represent the alpha diversity (Good 1953). Community comparison of harmful microalgae assemblages (beta diversity) was assessed using the Bray–Curtis distance, and their significant differences among the communities were determined by similarity analysis (ANOSIM) and PERMANOVA. Bray–Curtis distance‐based redundancy analysis (db‐RDA), Spearman's rank method, variation partitioning analysis (VPA) and linear regression were performed to reveal correlations between the community and physicochemical variables. Random forest analysis was used to examine important indicator taxa.

TITAN, which includes indicator species analysis (Dufrêne and Legendre 1997) and nonparametric change‐point analysis (nCPA) (King and Richardson 2003), is used for the determination of indicator values corresponding to each candidate change‐point along a gradient of environmental variables. Bootstrapping was used to identify the reliable indicator taxa. Taxa with fewer than three occurrences were excluded from data analysis. nCPA was conducted using a custom function within TITAN, based on the dbMRT method of De'ath (2002) in the ‘mvpart’ package (Baker and King 2010). The reliability (≥ 0.95) and purity (≥ 0.95) of the taxa as the minimum requirements. Additional details on TITAN have been furnished by Baker and King (2010).

## 3 Results

### 3.1 Variation in Environmental Parameters

Water quality parameters, nutrient levels, and stoichiometric ratios (C:N, C:P and N:P) assessed across a salinity gradient, considering seasonal variations (Tables S1–S3). The mean concentrations of DO in the HS group were considerably elevated at 7.22 ± 0.8 mg·L^−1^ surpassing the corresponding values in both the MS and LS groups (*p* < 0.001). Interestingly, the mean concentrations of NO_3_^−^‐N, NH_4_^+^‐N, and TP were markedly higher in the LS and MS groups compared to the HS group (*p* < 0.001). The mean temperature recorded across all seawater samples was 23.54 ± 5.85°C. Regarding salinity significant differences were observed across distinct seasons, with the lowest value (24.11 ± 3.38 ppt) documented during the summer 2020 and the highest value (29.74 ± 2.81 ppt) recorded in the winter 2020. In addition, the ratios of C:N and C:P exhibited the lowest values in the LS area and highest in the HS area (*p* < 0.001).

### 3.2 Composition and Diversity of Harmful Microalgae

**FIGURE 2:**
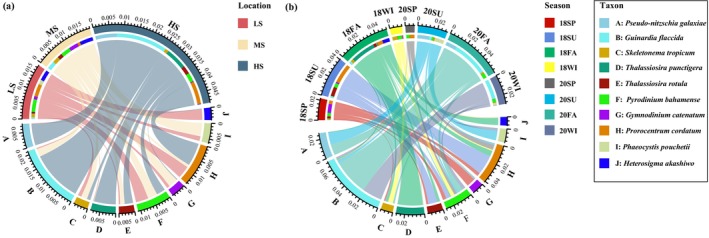
Relative abundance of the 10 most abundant harmful microalgae species in the microalgae community in different salinities (a) and seasons (b). HS, high‐salinity; LS, low‐salinity, MS, medium‐salinity.

**FIGURE 3:**
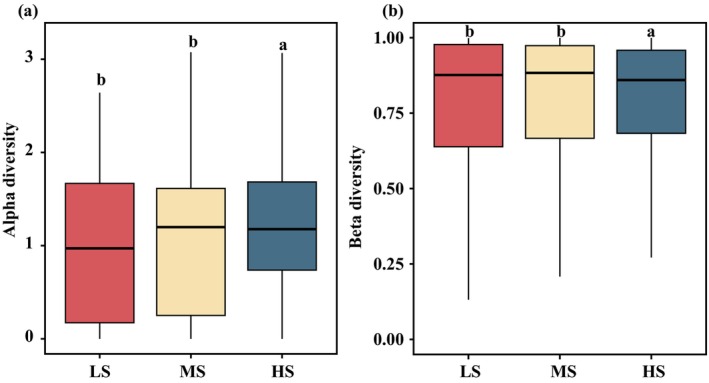
The alpha diversity (Shannon) and beta diversity among salinity areas. (a) The alpha diversity presented by boxplot; statistically significant differences (*p* < 0.05) are indicated by different areas. (b) Differences in harmful microalgae communities' beta diversity among salinity samples were estimated based on a Bray–Curtis distance matrix. The differences between pairs of two groups were tested by the Wilcoxon test. HS, high‐salinity; LS, Low‐salinity; MS, Medium‐salinity.

A total of 1,598,076 ± 256 sequences, constituting for 3.30% of the total sequence number was initially processed. Subsequently, the 607 ± 175 OTUs were acquired through clustering at a 97% similarity threshold, accounting for 21.14% of the total OTU pool. The Good's coverage, surpassing 97.61% for each sample, implies a comprehensive recovery of the majority of potentially hazardous algae. In this study, we identified 63 harmful microalgae species. Across all examined samples, the harmful microalgae community predominantly consisted of *G. flaccida*, *Pr. cordatum*, *T. punctigera*, *P. galaxiae* and *T. gravida* (Figure 2). The relative abundance of harmful microalgae species exhibited higher levels during the summer (SU) and autumn (Au) seasons compared to the spring (SP) and winter (WI) seasons (Figure 2b). *G. flaccida* emerged as the most prevalent species during the autumn and winter of 2020 (20Au and 20WI), whereas *T. punctigera* exhibited dominance in the autumn of 2018 (18Au). *T. gravida* and *Pr. cordatum* were mainly distributed during the summer of 2018 (18SU). Among the three salinity regions, the relative abundance of harmful microalgae species was observed to be highest in the HS region and lowest in the LS region across all samples (Figure 2a). The HS region exhibited a composition primarily dominated by *G. flaccida*, *Pr. cordatum*, and *P. galaxiae*. The relative abundance of *G. flaccida* and *T. gravida* demonstrated an upward trend concurrent with increasing salinity levels. Our findings revealed significant differences (*p* < 0.05) in alpha diversity, as measured by the Shannon indices, across distinct salinity zones and seasons (Figure 3a and Figure S2a). The alpha diversity indices in the MS group showed the highest mean values and in the LS group showed the lowest mean values among the three groups (Figure 3a). The alpha diversity of the harmful microalgae community reached its maximum value during the spring samples in 2018 exhibiting a statistically significant difference (*p* < 0.05) when compared to the alpha diversity values observed in other seasons (Figure S2a). The alpha diversity analysis of summer samples in 2020 revealed a statistically significant decrease (*p* < 0.05), indicating the lowest diversity compared to other seasons. Furthermore, no significant distinctions (*p* > 0.05) were observed in alpha diversities between the fall and winter groups. The observed pattern in alpha diversity during the transitional period from spring to autumn exhibited a distinctive temporal trend, characterized by an initial decrease followed by a subsequent increase in both 2018 and 2020 (Figure S2a).

The beta diversity based on Bray–Curtis distances revealed variation between the harmful microalgae communities from all samples (Figure 3b and Figure S2b). The harmful microalgae communities showed the highest beta diversity in the MS group, indicating a higher dispersion (Figure 3b). During the eight seasons examined, the beta diversity exhibited its highest values in the summer and lowest in the spring (Figure S2b). Harmful microalgae community composition among compartments, conducted through PERMANOVA utilizing the Bray–Curtis distance, yielded statistically significant differences (*R* ^2^ = 0.341, *p* < 0.001). Within each salinity zone, the harmful microalgae communities were distinct and showed a significant difference (ANOSIM, *R* ^2^ ≥ 0.043, *p* < 0.001) (Figure 3b). The ANOSIM test (*R* ^2^ = 0.155, *p* < 0.001) revealed a significantly different harmful microalgae community among the eight seasonal samples.

### 3.3 Relationships Between Harmful Microalgae Communities and Environmental Factors

**FIGURE 4:**
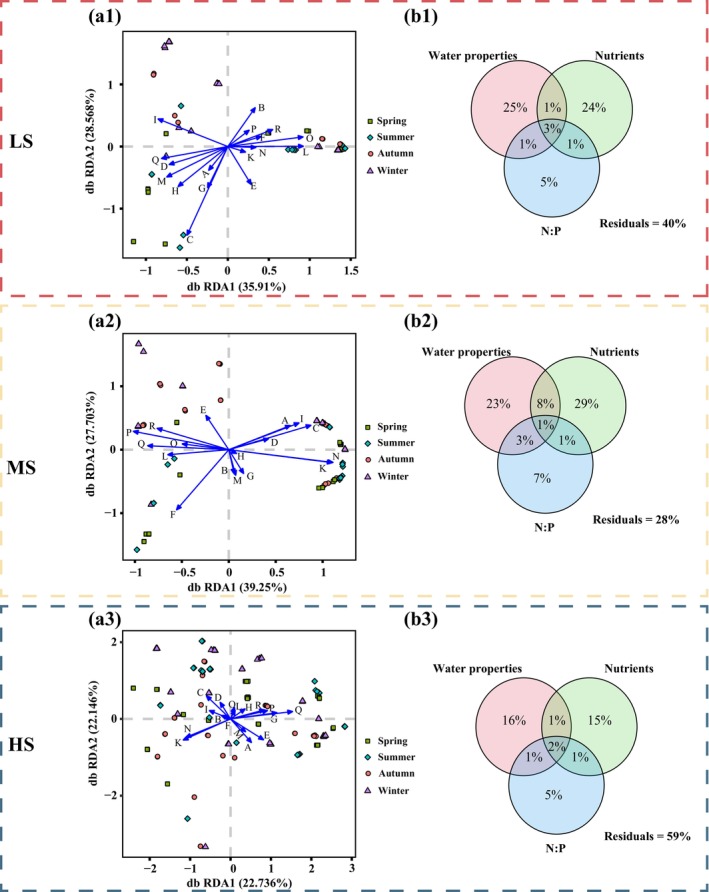
Correlation of harmful microalgae communities with environmental factors along salinity gradients. (a1–3) Distance‐based redundancy analysis (dbRDA) ordination plots of the relationship between harmful microalgae communities and measured environmental variables. A: Temperature; B: Salinity; C: PH; D: Chl‐*a*; E: COD; F: DO; G: NO_2_^−^‐N; H: NO_3_^−^‐N; I: NH_4_^+^‐N; J: DIN; K: DIP; L: TOC; M: TN; N: TP; O: C:N; P: C:P; Q: N:P; R: C:N:P. (b1–3) Variation partitioning analysis of the effects of water quality parameters (temperature, pH, salinity, Chl‐*a*, DO, and COD), nutrient levels (NO_2_^−^‐N, NO_3_^−^‐N, NH_4_^+^‐N, DIN, TN, DIP, TOC, and TP), and N:P on the harmful microalgae community composition in seawater from the Dafengjiang River.

The impact of the environmental factors on the harmful microalgae community structure and distribution was used using db‐RDA (Figure 4a1–3). The findings revealed a notable correlation between nearly all environmental variables and the harmful microalgae community. However, it was observed that the influencing factors varied among salinity samples. NH_4_^+^‐N, TP and DIP presented more significant impacts on beta diversity within the LS, MS and HS samples, respectively (*p* < 0.001), surpassing the influence of other variables. Spearman's correlation analysis revealed statistically significant positive correlations between NH_4_^+^‐N, TP and DIP and alpha diversity in LS, MS and HS groups, respectively (Table S4). Moreover, there was a significant negative correlation between the N:P ratios and alpha diversity across all salinity regions. Seawater properties, nutrients, and N:P collectively accounted for 41% of the community changes in the harmful microalgae within the HS area and more than 60% in the LS and MS areas (Figure 4b1–3). In LS and HS regions, the pure effects of nutrients were 24% and 15%, respectively, which were only 1% smaller than those of seawater properties. In MS area, the pure effects of nutrients (29%) were higher than those of seawater properties (23%). Furthermore, it is noteworthy that the contribution of the N:P ratio consistently exhibited lower magnitudes compared to the influence exerted by water parameters and nutrient levels.

### 3.4 Community‐Level Change Points of Harmful Microalgae

**FIGURE 5:**
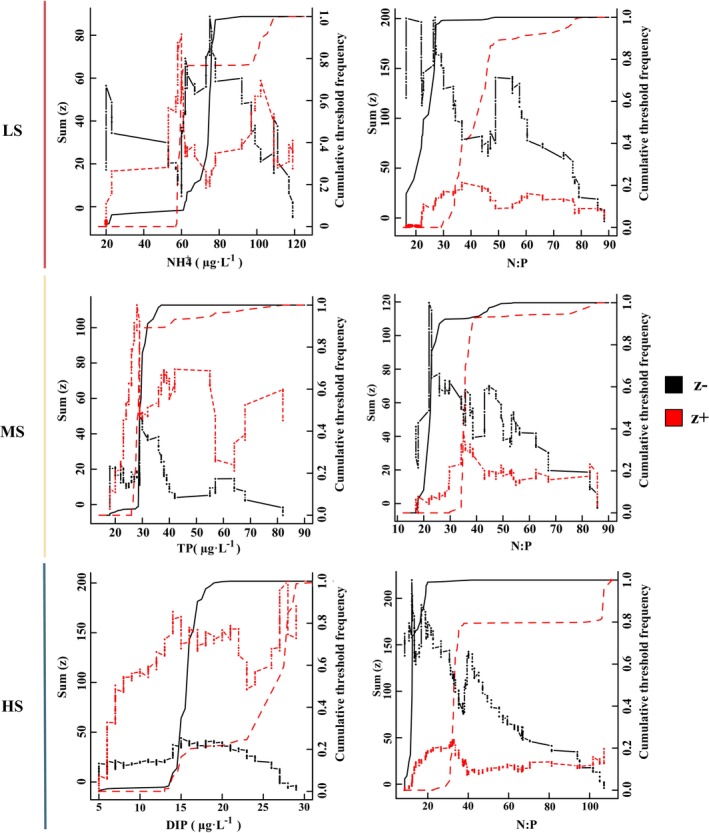
Threshold Indicator Taxa Analysis (TITAN) sums of negative (z^−^) and positive (z^+^) responding species to all candidate change points along nutrient gradients. The lines represent cumulative frequency distributions of change points for z^−^ (black) and z^+^ (red) taxa, respectively.

The identification of harmful microalgae responsive to crucial environmental variables within each salinity region in the Dafengjiang River estuary was accomplished through the TITAN (Figure 5 and Figure S4). The nCPA of NH_4_^+^‐N and N:P in LS area peaked at 57.7 μg·L^−1^ and 29.62, and yielded bootstrap frequency distributions similar to sum (z−). Likewise, the nCPA deviance reduction for TP in the MS area, as well as for DIP and N:P in the HS area, reached its maximum at 28.5 μg·L^−1^, 14.5 μg·L^−1^ and 12.20:1, respectively, closely approaching the negative threshold decline. In the MS area, the observed peaks in the nCPA deviance reduction for N:P occurred at 35.38, with trends closely resembling the sum (z+). Thus, the applicable change points for N:P were 40.33:1, 22.47:1 and 32.89:1, respectively, from the LS sample to the HS sample. And the change points of NH_4_^+^‐N, TP and DIP were 60, 28.5 and 28 μg·L^−1^, respectively.

### 3.5 Indicator Species

**FIGURE 6:**
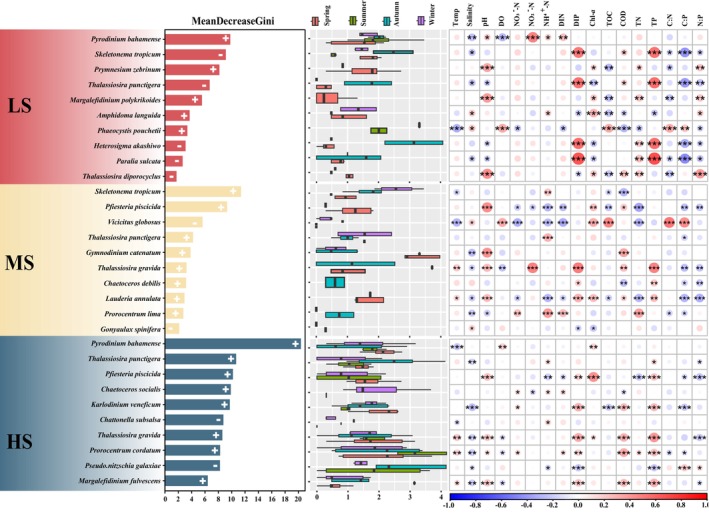
Random forest classification of the top 10 important species in different areas. Left: The top 10 taxa analysed using the Gini index, representing the importance of each species in distinguishing different groups. + and − represent positive and negative responding species, respectively. Middle: Read abundances of the top 10 species. Right: Pearson correlations between the relative abundances of the top 10 species and environmental and nutrient factors. *P < 0.05; **P < 0.01; ***P < 0.001.

The random forest method was employed to identify the most important harmful microalgae species across seasonal samples (Figure 6). The 10 most important indicator species, determined by their maximal Gini values, were displayed for each group. *Py. bahamense*, *S. tropicum* and *Prymnesium zebrinum* emerged as pivotal indicator species within the LS zone. In the MS zone, *S. tropicum*, *Pfiesteria piscicida* and *Vicicitus globosus* emerged as pivotal indicators. Conversely, within the HS zone *Py. bahamense*, *T. punctigera* and *Pf. piscicida* were identified as the foremost positive‐response indicator species. Certain harmful microalgae species demonstrated diverse adaptabilities to salinity, thereby serving as potential indicators for distinct ecological groups. Spearman's correlation analysis showed that the 10 indicator species within each group were significantly associated with multiple environmental factors. For instance, in the LS region, *Py. bahamense* displayed significant positive correlations with pH, NH_4_^+^‐N and DIP and significant negative correlations with DO. In the MS zone, both *V. globosus* and *S. tropicum* demonstrated significant positive correlations with temperature. In the HS region, *K. veneficum* and *T. gravida* had similar characteristics, both of which were significantly positively correlated with DIP, COD and TP and negatively correlated with salinity.

## 4 Discussion

HABs have recurrently manifested in coastal regions over the recent decades and imparting deleterious impacts upon the environment by inducing toxicity and precipitating the mortality of marine fauna (O'Neill 2020; Shi et al. 2022). Prior studies on harmful microalgae species have predominantly concentrated on the assessment of species diversity and the principal environmental determinants influencing the diversity (Guo et al. 2007; Tian and Lv 2010; Xu et al. 2003; Wang et al. 2022). However, the distribution of harmful microalgae species and identification of critical transition points in community dynamics within subtropical estuarine ecosystems constitute areas of limited comprehension in current scientific understanding. The current study involved the analysis of water samples collected from the Dafengjiang River along a salinity gradient during different seasons. The findings indicate that the environmental threshold of the N:P ratio exhibits a greater breadth in the HS area compared to other salinity zones, and *Pf. piscicida*, *Pr. cordatum*, *Py. bahamense* and *T. punctigera* emerged as particularly significant bioindicators within subtropical estuaries. Overall, this study explores the implications of abrupt transitions in the harmful microalgae community and their potential effects on marine ecosystems, providing scientific guidance for the control of harmful microalgae in subtropical estuaries.

### 4.1 Distribution and Potential Risk of Harmful Microalgae Species

This study investigated the seasonal and salinity variations in the diversity of harmful microalgae within a subtropical estuarine ecosystem. A total of 63 harmful microalgae species were identified. Notably, *G. flaccida*, *Pr. cordatum, T. punctigera*, *P. galaxiae* and *T. gravida* were the most abundant species (Figure 2), belonging to the phyla Bacillariophyta and Dinoflagellata, indicating their broad ecological niches in interspecies competition. Consistent with previous studies on the Sindh coast (Pakistan) and the East China Sea, our study demonstrated that *G. flaccida* exhibits pronounced dominance in high‐salinity regions (Figure 2a) (Khokhar et al. 2022; Guo, Sun, and Dai 2012). *P. galaxiae* was predominant during the summer and autumn in the Dafengjiang Estuary (Figure 2b), which contrasts with the findings of Ruggiero et al. (2015), who reported its peak abundance during the winter in the Gulf of Naples. These results underscore the influence of local environmental factors, including temperature and nutrient availability, on the distribution of harmful microalgae. In this study, certain toxic and potentially harmful was detected. For example, *Pr. cordatum* was mainly distributed in HS region of Dafengjiang River estuary. *Pr. cordatum* is recognized for causing HABs in numerous coastal estuarine ecosystems worldwide (Hajdu, Pertola, and Kuosa 2005; Heil, Glibert, and Fan 2005; Johnson 2015; Stoecker et al. 1997). Studies reported that, *Pr. cordatum* produces diarrhetic shellfish poisoning (DSP) toxins, which are associated with severe health issues, including poisoning or death from the consumption of contaminated shellfish (Silva 1985; Silva and Sousa 1981), oysters (Akiba and Hattori 1949) and clams (Nakazima 1965b, 1965a). Therefore, understanding the distribution and diversity of harmful microalgae is crucial for safeguarding both marine ecosystems and public health.

In the present study, it was observed that alpha diversity displayed notable variation, with the maximum values discerned in the samples derived from the MS samples, whereas the minimum values were recorded in those obtained from the LS samples (Figure 3a). Furthermore, our study revealed a significant positive correlation between alpha diversity and salinity (Figure S3). Olli, Tamminen, and Ptacnik (2023) observed a positive correlation between phytoplankton community diversity and salinity in Chesapeake Bay and the Baltic Sea, specifically noting an increase when salinity exceeded 10%. These findings suggest that alterations in salinity levels may potentially enhance the diversity of harmful microalgae community Nonetheless, it is important to acknowledge that elevated salinity, beyond a certain threshold, could induce stress, thereby potentially diminishing harmful microalgae community diversity (D'ors, Bartolomé, and Sánchez‐Fortún 2016). The temporal changes in community diversity of harmful microalgae within subtropical estuaries were elucidated in the present study. The peak of alpha diversity was observed during spring, while the lowest levels were recorded during summer, and one possible explanation being the less favourable conditions (e.g., high temperature (Xu et al. 2016; Gonzalez‐Camejo et al. 2019), hypoxia (Polikarpov, Saburova, and Al‐Yamani 2020) and increased nutrient load (Al‐Said et al. 2017)) for microalgae development during the summer seasons (Polikarpov, Saburova, and Al‐Yamani 2020). Our results are consistent with those of previous studies (Polikarpov, Saburova, and Al‐Yamani 2020; Slegers et al. 2013), indicative of the potential influence of temperature fluctuations on the diversity of the harmful microalgae community in subtropical estuaries. The beta diversity was significantly different between the MS and HS samples and showed the largest mean values in the MS samples. Uneven nutrient distribution and environmental heterogeneity in transition zones may contribute to increased beta diversity by influencing ecological interactions, such as species competition and niche differentiation (Pitacco et al. 2019; Chen et al. 2020). Our results indicate that the harmful microalgae community structure exhibits specific characteristics and variations with environmental changes in subtropical estuaries.

### 4.2 Nutrients Affecting the Harmful Microalgae Community

NH_4_^+^‐N, TP and DIP were identified as the main environmental factors that influenced the variation in the structure of harmful microalgae communities in the Dafengjiang River estuary (Figure 4a1–3). Consistent with prior studies, these findings align with the established literature as key determinants in shaping the dynamics of the harmful microalgae communities (Yang et al. 2021; McNaughton 2019; Wang et al. 2022). For instance, McNaughton (2019) found that NH_4_^+^‐N was the main nutrient for phytoplankton taxon abundance and diversity, and its concentration was associated with a significant increase in the abundance of flagellate taxa and calanoid copepods (*p* < 0.05). The distribution of phytoplankton communities was notably influenced by TP and PO_4_^3−^‐P with particular emphasis on their significant impact on phytoplankton density (*p* < 0.01) in the Haihe River Basin, China (Wu et al. 2022). Wang et al. (2022) found that nutrients had a great impact on HAB species, and it was significantly positively correlated with PO_4_^3−^‐P in the South China Sea. HABs substantial impact on nutrient levels in estuarine ecosystems. During HAB events, microalgae typically absorb substantial quantities of nutrients such as NH_4_^+^‐N, TP and DIP, leading to a reduction in their concentrations (Parial and Dey 2023). Conversely, the decomposition of microalgae following HABs can release these nutrients back into the water, potentially altering nutrient ratios and influencing subsequent algal growth (Wang et al. 2021). These findings suggest that the harmful microalgae communities are primarily influenced by multiple nutrients. In this study, the pure effects of nutrients (e.g., N and P) from LS to HS were 24%, 29% and 15% (Figure 4b1–3), indicating that nitrogen and phosphorus play an important role in the change and turnover of PRM communities. Additionally, we found that N:P was also an important driver of harmful microalgal community structure in subtropical estuaries. Our previous study reported that the ratio of nitrogen to phosphorus (N:P) had a notable impact on the beta diversity of the phytoplankton community (Xu et al. 2022). Nhu et al. (2019) found that as the N:P ratios increased, the species richness of the overall phytoplankton community decreased. This may because N:P ratios affect the growth of phytoplankton, including harmful microalgae such as dinoflagellates, through mechanisms involving carbon metabolism (carbon fixation, photosynthesis, biomolecular synthesis and metabolic regulation) and phosphorus metabolism (energy metabolism, cell division and growth) (Brembu et al. 2017). Overall, multiple nutrients have been proven to be important factors affecting the structure of harmful microalgae communities, and further understanding of their thresholds is critical for ecological protection.

### 4.3 Harmful Microalgae Exhibit Broad Environmental Adaptability in High‐Salinity Area

Nitrogen and phosphorus are essential nutrients in estuarine ecosystems, but excessive levels can lead to eutrophication, resulting in HABs and deteriorating water quality (Zhang et al. 2023). In this study, we used TITAN to find that the ecological threshold for TP ranges from 27.8 to 28.5 μg·L^−1^ (Figure 5 and Figure S4). This indicates that exceeding this range may trigger blooms, cause water quality deterioration, and even lead to ecological imbalance. Consistent with our study, Dodds, Smith, and Lohman (2002) and Stevenson et al. (2006) used Chl‐*a* analysis to determine a TP threshold of 30 μg·L^−1^. This suggests that different habitats respond similarly to TP levels, supporting the reliability of our findings. Furthermore, this study provides strong evidence of a wider range of N:P environmental breadths for the harmful microalgae community in the HS area than in the LS and MS areas (Figure 5), indicating that the harmful microalgae community in the HS area can maintain a relatively stable ecological balance across a broader range of nitrogen and phosphorus concentrations. In LS area, nutrient enrichment from river input and aquaculture lead to the excessive growth of harmful microalgae (Tang et al. 2017; Liu et al. 2020; Burian et al. 2016). This, in turn, triggers resource competition or niche changes, which may cause more frequent turnover of harmful microalgae communities (Liu et al. 2020; Burian et al. 2016). This suggests that the harmful microalgae community exhibited greater adaptability to varying nutrient conditions in high‐salinity areas, contributing to the maintenance of ecosystem stability (Stefanidou et al. 2018; Larson and Belovsky 2013). In summary, this study establishes a comprehensive framework for microalgal blooms using TITAN and offers valuable insights for water quality management. Future research should focus on conducting longer‐term experiments, combining molecular analysis with microscopy to improve the reliability and scientific rigor, and examining the interactions of multiple environmental factors to validate and refine environmental thresholds.

### 4.4 HABs Indicator Species in Dafengjiang Estuary

Microalgae can respond rapidly to environmental disturbance and play an important role in biological monitoring as indicator taxa for the detection and evaluation of environmental disturbances in aquatic ecosystems (Inyang, Wang, and Cheng 2022; O'Neill and Rowan 2022). In the present study, it was observed that *Py. bahamense, Pf. piscicida, S. tropicum* and *T. punctigera* emerged as crucial indicator species within different salinity zones and exhibiting sensitivity to environmental changes within the Dafengjiang River Estuary (Figure 6). The ability of these harmful microalgae species to induce environmental changes may be related to their characteristics. Consistent with Burkholder and Glasgow Jr (1997), we found that *Pf. piscicida* was significantly positively correlated with DIP and TP (*p* < 0.05; Figure 6). Previous studies have shown that *Pfiesteria* possess endogenous phosphatase activity, enabling them to survive in low‐phosphorus environments and effectively utilize phosphorus (Skelton, Parrow, and Burkholder 2006). *Py. bahamense* exhibited a positive correlation with NH_4_^+^‐N in the LS group (*p* < 0.05; Figure 6). *Py. bahamense* may preferentially absorb and utilize NH₄^+^‐N, especially in eutrophicated waters, where this easily absorbed nitrogen source facilitates its rapid growth and the formation of HABs (Wagey 2002; Brewton 2023; Sugimoto 1998). Similarly, *S. tropicum* is a warm‐water species that predominates mainly in warmer waters and is relatively rare in colder waters (Yamada, Otubo, and Tada 2022). Ueno (1993) found that the cell abundance of *S. tropicum* experienced a rapid decline at temperatures below 25°C and ceased to exist when seawater temperatures dropped below 12°C (Liu et al. 2012). In our study, a negative correlation with temperature (*p* < 0.05; Figure 6) was observed for *S. tropicum* in the MS group, where the average water temperature was 23.18°C. Collectively, these indicator species facilitate the assessment of nutrient changes and their subsequent ecological effects on estuarine ecosystems.

## 5 Conclusions

This study explored the community structure and community‐level change points of the harmful microalgae community in the Dafengjiang River Estuary. The harmful microalgae community comprised 63 species and was dominated by *G. flaccida*, *Pr. cordatum*, *T. punctigera*, *P. galaxiae* and *T. gravida*. NH_4_^+^‐N, TP and DIP were key drivers affecting variation in the harmful microalgae community and their nutrient criteria were 57.5–60, 27.8–28.5 and 14.5–28 μg·L^−1^, respectively. The harmful microalgae community showed stronger environmental adaptation in high‐salinity areas than in low‐salinity areas. *Py. bahamense, Pf. piscicida, S. tropicum* and *T. punctigera* can be used as indicator species to reflect the environmental changes in the study areas. This study establishes a scientific basis for understanding the nutrient criteria that impact species niches and harmful microalgae community stability in response to climate change and anthropogenic effects in subtropical estuaries.

## Author Contributions

**Jiongqing Huang:** conceptualization (equal), methodology (equal), writing – review and editing (equal). **Huaxian Zhao:** writing – review and editing (equal). **WeiJun Wang:** conceptualization (equal), methodology (equal), writing – review and editing (equal). **Xinyi Qin:** investigation (equal), supervision (equal). **Pengbin Wang:** writing – review and editing (equal). **Qinghua Hou:** writing – review and editing (equal). **Qingxiang Chen:** writing – review and editing (equal). **Gonglingxia Jiang:** formal analysis (equal), visualization (equal). **Ke Dong:** writing – review and editing (equal). **Tao Jiang:** supervision (equal), validation (equal), writing – review and editing (equal). **Yang Pu:** conceptualization (equal), data curation (equal), formal analysis (equal), investigation (equal), methodology (equal), visualization (equal), writing – original draft (equal), writing – review and editing (equal). **Nan Li:** conceptualization (equal), data curation (equal), formal analysis (equal), investigation (equal), methodology (equal), visualization (equal), writing – original draft (equal), writing – review and editing (equal).

## Conflicts of Interest

The authors declare no conflicts of interest.

## Supporting information


**Figure S1.** Principal component analysis of environmental parameters.
**Figure S2.** The alpha diversity (Shannon) and beta diversity during different seasons. A) The alpha diversity presented by boxplot, statistically significant differences (*p* < 0.05) were indicated by different seasons. B) Differences in harmful microalgae community beta diversity among salinity samples were estimated based on a Bray–Curtis distance matrix. The differences between pairs of two groups were tested by the Wilcoxon test.
**Figure S3.** Linear regressions for salinity associated with harmful microalgae community alpha diversity (Shannon).
**Figure S4.** The deviance reduction in the Bray–Curtis distance values for the candidate change points of the harmful microalgae community along nutrient gradients and the cumulative frequency distribution of the change points among the bootstrap replicates. The dashed blue lines represent the cumulative frequency distributions of change points.

**Table S1.** Physical and biochemical properties of seawater in different areas.
**Table S2.** One‐way ANOVA test on variation of each water chemical parameter in different areas.
**Table S3.** One‐way ANOVA test on variation of each water chemical parameter during different seasons.
**Table S4.** Spearman correlations between the harmful microalgae community alpha diversity (Shannon) and environmental factors.

## Data Availability

The datasets presented in this study can be found in the NCBI SRA under BioProjects PRJNA1044415 and PRJNA866330, with access numbers ranging from SRR28160689 to SRR28161153 and SRR22029102 to SRR22029251.
