# Supplementary material for: Harmful Microalgae Exhibit Broad Environmental Adaptability in High‐Salinity Area Across the Dafengjiang River Estuary
Source: Ecol Evol. 2024 Oct 23;14(10):e70455. doi: 10.1002/ece3.70455 (PMC11496773; doi:10.1002/ece3.70455)
Supplement: Supplementary file 1 — Figure S1. Principal component analysis of environmental parameters. Figure S2. The alpha diversity (Shannon) and beta diversity during different seasons. A) The alpha diversity presented by boxplot, statistically significant differences (p < 0.05) were indicated by different seasons. B) Differences in harmful microalgae community beta diversity among salinity samples were estimated based on a Bray–Curtis distance matrix. The differences between pairs of two groups were tested by the Wilcoxon test. Figure S3. Linear regressions for salinity associated with harmful microalgae community alpha diversity (Shannon). Figure S4. The deviance reduction in the Bray–Curtis distance values for the candidate change points of the harmful microalgae community along nutrient gradients and the cumulative frequency distribution of the change points among the bootstrap replicates. The dashed blue lines represent the cumulative frequency distributions of change points. [file ECE3-14-e70455-s001.docx]

**Supplementary Information (Figure S1-S4)**

**Harmful microalgae exhibit broad environmental adaptability in high-salinity area across subtropical estuaries**

**Jiongqing Huang ^1, 2^, Huaxian Zhao ^3^, WeiJun Wang ^1^, Xinyi Qin ^2, 3^,** **Pengbin Wang ^4^, Qinghua Hou ^2^, Qingxiang Chen ^2^, Gonglingxia Jiang ^2^, Ke Dong ^5^, Tao Jiang ^6^, Yang Pu ^1, *^, Nan Li ^2, *^**

^1^ School of Agriculture, Ludong University, Yantai, China

^2^ Laboratory for Coastal Ocean Variation and Disaster Prediction, College of Ocean and Meteorology; Key Laboratory of Climate, Resources and Environment in Continental Shelf Sea and Deep Sea of Department of Education of Guangdong Province, Guangdong Ocean University, Zhanjiang, China

^3^ Key Laboratory of Environment Change and Resources use in Beibu Gulf, Ministry of Education, Nanning Normal University, Nanning, China

^4^ Key Laboratory of Marine Ecosystem Dynamics, Second Institute of Oceanography, Ministry of Natural Resources, Hangzhou, China

^5^ Department of Biological Sciences, Kyonggi University, Gyeonggi-do, South Korea

^6^ School of Ocean, Yantai University, Yantai, China

**# Correspondence:**

* Corresponding Author: Yang Pu

ypu@ldu.edu.cn

* Corresponding Author: Nan Li

[nli0417@163.com](mailto:nli0417@163.com)


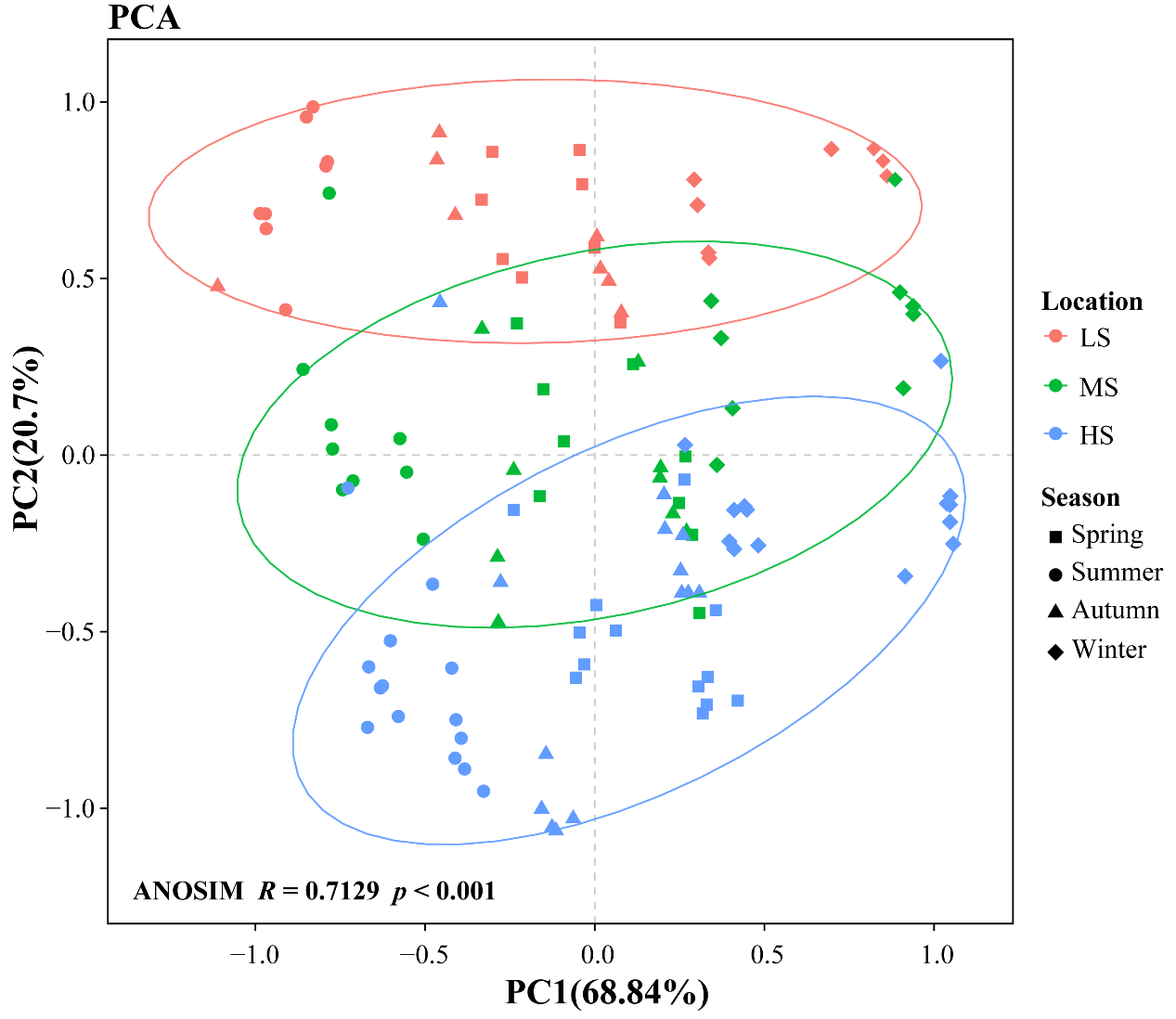


**FIGURE S1** Principal component analysis of environmental parameters.


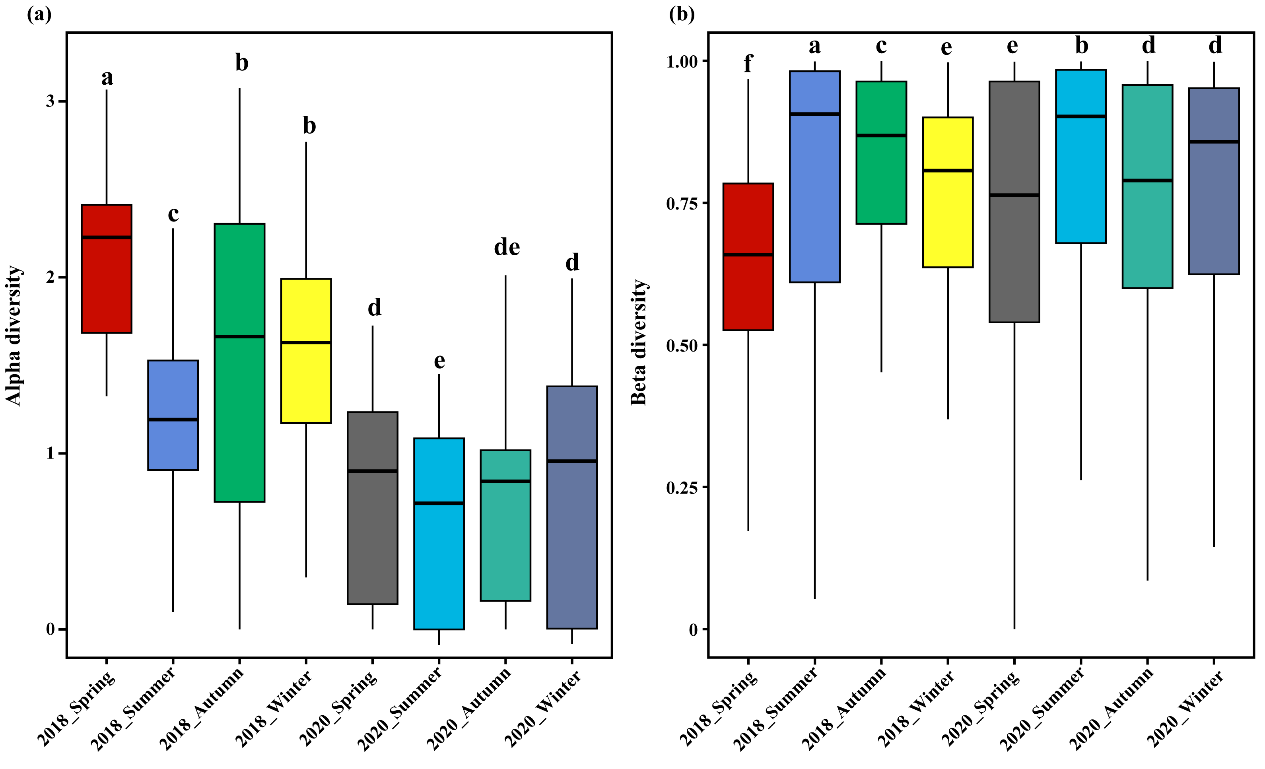


**FIGURE S2** The alpha diversity (Shannon) and beta diversity during different seasons. A) The alpha diversity presented by boxplot, statistically significant differences (*p* < 0.05) were indicated by different seasons. B) Differences in harmful microalgae community beta diversity among salinity samples were estimated based on a Bray–Curtis distance matrix. The differences between pairs of two groups were tested by the Wilcoxon test.


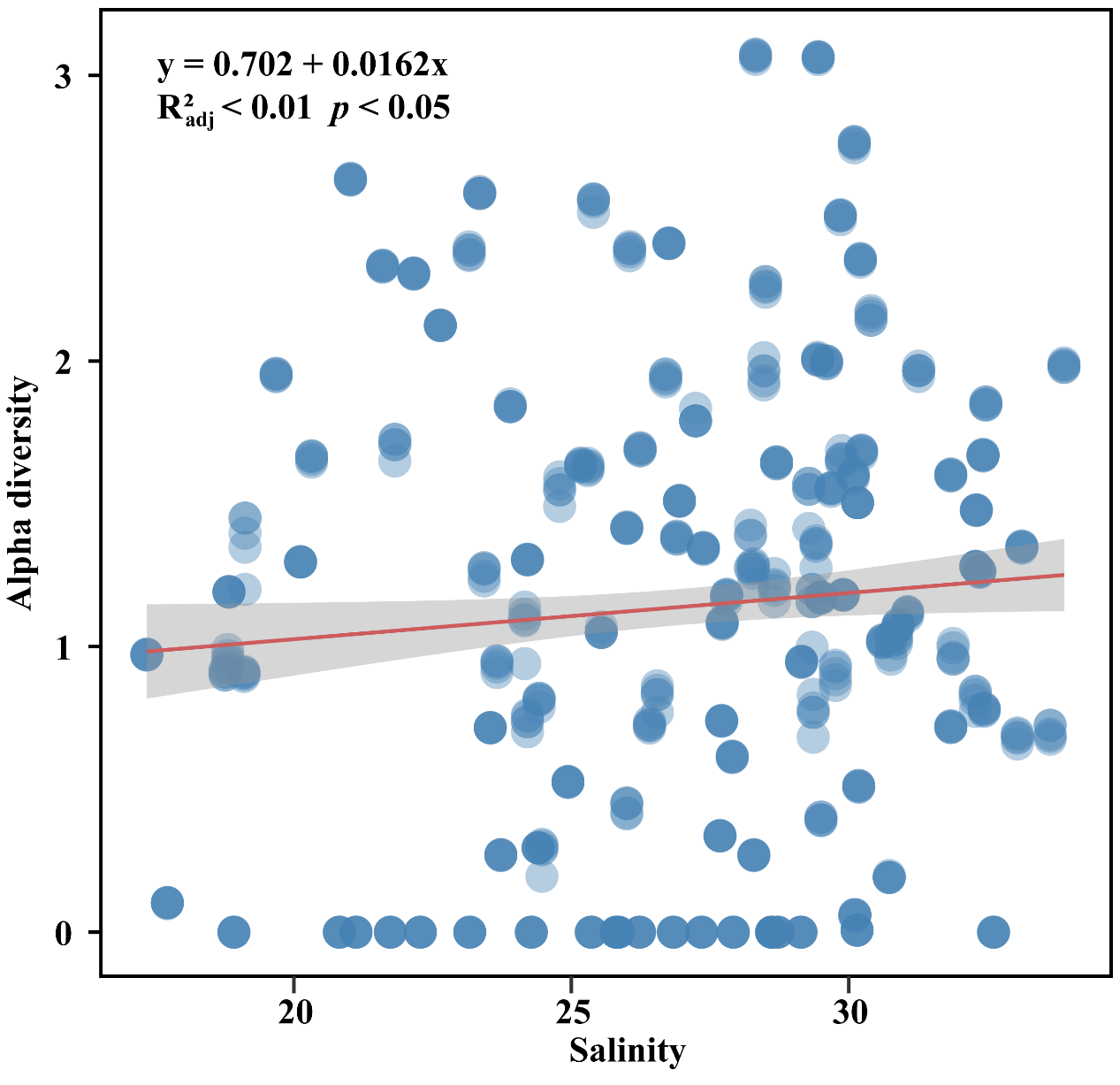
 **FIGURE S3** Linear regressions for salinity associated with harmful microalgae community alpha diversity (Shannon).

**
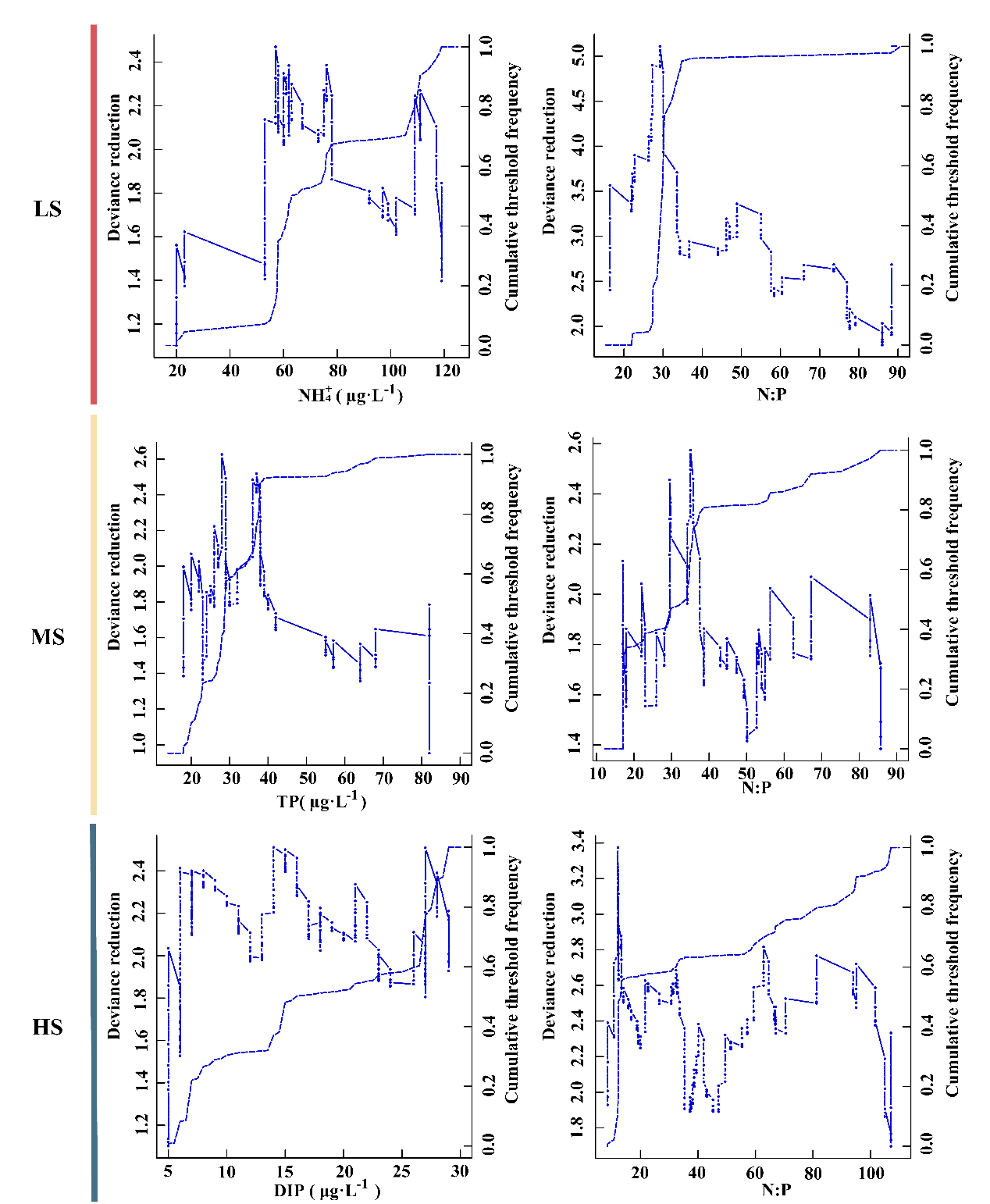
**

**FIGURE S4** The deviance reduction in the Bray–Curtis distance values for the candidate change points of the harmful microalgae community along nutrient gradients and the cumulative frequency distribution of the change points among the bootstrap replicates. The dashed blue lines represent the cumulative frequency distributions of change points.
