# Supplementary material for: Harmful Microalgae Exhibit Broad Environmental Adaptability in High‐Salinity Area Across the Dafengjiang River Estuary
Source: Ecol Evol. 2024 Oct 23;14(10):e70455. doi: 10.1002/ece3.70455 (PMC11496773; doi:10.1002/ece3.70455)
Supplement: Supplementary file 2 — Table S1. Physical and biochemical properties of seawater in different areas. Table S2. One‐way ANOVA test on variation of each water chemical parameter in different areas. Table S3. One‐way ANOVA test on variation of each water chemical parameter during different seasons. Table S4. Spearman correlations between the harmful microalgae community alpha diversity (Shannon) and environmental factors. [file ECE3-14-e70455-s002.docx]

**Supplementary Information (Table S1-S4)**

**Harmful microalgae exhibit broad environmental adaptability in high-salinity area across subtropical estuaries**

**Jiongqing Huang ^1, 2^, Huaxian Zhao ^3^, WeiJun Wang ^1^, Xinyi Qin ^2, 3^,** **Pengbin Wang ^4^, Qinghua Hou ^2^, Qingxiang Chen ^2^, Gonglingxia Jiang ^2^, Ke Dong ^5^, Tao Jiang ^6^, Yang Pu ^1, *^, Nan Li ^2, *^**

^1^ School of Agriculture, Ludong University, Yantai, China

^2^ Laboratory for Coastal Ocean Variation and Disaster Prediction, College of Ocean and Meteorology; Key Laboratory of Climate, Resources and Environment in Continental Shelf Sea and Deep Sea of Department of Education of Guangdong Province, Guangdong Ocean University, Zhanjiang, China

^3^ Key Laboratory of Environment Change and Resources use in Beibu Gulf, Ministry of Education, Nanning Normal University, Nanning, China

^4^ Key Laboratory of Marine Ecosystem Dynamics, Second Institute of Oceanography, Ministry of Natural Resources, Hangzhou, China

^5^ Department of Biological Sciences, Kyonggi University, Gyeonggi-do, South Korea

6 School of Ocean, Yantai University, Yantai, China

**# Correspondence:**

* Corresponding Author: Yang Pu

ypu@ldu.edu.cn

* Corresponding Author: Nan Li

[nli0417@163.com](mailto:nli0417@163.com)

**TABLE S1** Physical and biochemical properties of seawater in different areas.

| **Sites** | **Temperature**  **(°C)** | **Salinity**  **(ppt)** | **pH** | **DO**  **(mg/L)** | **NO_3_^-^****-N(mg/L)** | **NO_2_^-^-N**  **(mg/L)** | **NH_4_^+^-N**  **(mg/L)** | **DIN**  **(mg/L)** | **DIP**  **(mg/L)** | **Chl-*a***  **(mg/L)** | **TOC**  **(mg/L)** | **COD**  **(mg/L)** | **TN**  **(mg/L)** | **TP**  **(mg/L)** |
| --- | --- | --- | --- | --- | --- | --- | --- | --- | --- | --- | --- | --- | --- | --- |
| 18AU_01.1 | 26.3 | 20.12 | 7.73 | 6.7 | 0.606 | 0.072 | 0.06 | 0.738 | 0.046 | 1.6 | 4.19 | 2.35 | 1.369 | 0.115 |
| 18AU_01.2 | 26.3 | 20.12 | 7.73 | 6.7 | 0.606 | 0.072 | 0.06 | 0.738 | 0.046 | 1.6 | 4.19 | 2.35 | 1.369 | 0.115 |
| 18AU_01.3 | 26.3 | 20.12 | 7.73 | 6.7 | 0.606 | 0.072 | 0.06 | 0.738 | 0.046 | 1.6 | 4.19 | 2.35 | 1.369 | 0.115 |
| 18AU_01.4 | 26.3 | 20.12 | 7.73 | 6.7 | 0.606 | 0.072 | 0.06 | 0.738 | 0.046 | 1.6 | 4.19 | 2.35 | 1.369 | 0.115 |
| 18AU_01.5 | 26.3 | 20.12 | 7.73 | 6.7 | 0.606 | 0.072 | 0.06 | 0.738 | 0.046 | 1.6 | 4.19 | 2.35 | 1.369 | 0.115 |
| 18AU_02.1 | 26 | 19.68 | 7.83 | 6.8 | 0.586 | 0.047 | 0.075 | 0.708 | 0.04 | 1.8 | 3.91 | 2.73 | 1.028 | 0.1 |
| 18AU_02.2 | 26 | 19.68 | 7.83 | 6.8 | 0.586 | 0.047 | 0.075 | 0.708 | 0.04 | 1.8 | 3.91 | 2.73 | 1.028 | 0.1 |
| 18AU_02.3 | 26 | 19.68 | 7.83 | 6.8 | 0.586 | 0.047 | 0.075 | 0.708 | 0.04 | 1.8 | 3.91 | 2.73 | 1.028 | 0.1 |
| 18AU_02.4 | 26 | 19.68 | 7.83 | 6.8 | 0.586 | 0.047 | 0.075 | 0.708 | 0.04 | 1.8 | 3.91 | 2.73 | 1.028 | 0.1 |
| 18AU_02.5 | 26 | 19.68 | 7.83 | 6.8 | 0.586 | 0.047 | 0.075 | 0.708 | 0.04 | 1.8 | 3.91 | 2.73 | 1.028 | 0.1 |
| 18AU_03.1 | 36 | 20.32 | 7.77 | 6.8 | 0.548 | 0.049 | 0.092 | 0.689 | 0.021 | 1.7 | 3.6 | 2.68 | 1.224 | 0.047 |
| 18AU_03.2 | 36 | 20.32 | 7.77 | 6.8 | 0.548 | 0.049 | 0.092 | 0.689 | 0.021 | 1.7 | 3.6 | 2.68 | 1.224 | 0.047 |
| 18AU_03.3 | 36 | 20.32 | 7.77 | 6.8 | 0.548 | 0.049 | 0.092 | 0.689 | 0.021 | 1.7 | 3.6 | 2.68 | 1.224 | 0.047 |
| 18AU_03.4 | 36 | 20.32 | 7.77 | 6.8 | 0.548 | 0.049 | 0.092 | 0.689 | 0.021 | 1.7 | 3.6 | 2.68 | 1.224 | 0.047 |
| 18AU_03.5 | 36 | 20.32 | 7.77 | 6.8 | 0.548 | 0.049 | 0.092 | 0.689 | 0.021 | 1.7 | 3.6 | 2.68 | 1.224 | 0.047 |
| 18AU_04.1 | 26 | 21.12 | 7.83 | 6.8 | 0.557 | 0.079 | 0.103 | 0.739 | 0.015 | 2 | 3.17 | 2.23 | 1.127 | 0.032 |
| 18AU_04.2 | 26 | 21.12 | 7.83 | 6.8 | 0.557 | 0.079 | 0.103 | 0.739 | 0.015 | 2 | 3.17 | 2.23 | 1.127 | 0.032 |
| 18AU_04.3 | 26 | 21.12 | 7.83 | 6.8 | 0.557 | 0.079 | 0.103 | 0.739 | 0.015 | 2 | 3.17 | 2.23 | 1.127 | 0.032 |
| 18AU_04.4 | 26 | 21.12 | 7.83 | 6.8 | 0.557 | 0.079 | 0.103 | 0.739 | 0.015 | 2 | 3.17 | 2.23 | 1.127 | 0.032 |
| 18AU_04.5 | 26 | 21.12 | 7.83 | 6.8 | 0.557 | 0.079 | 0.103 | 0.739 | 0.015 | 2 | 3.17 | 2.23 | 1.127 | 0.032 |
| 18AU_05.1 | 26 | 23.35 | 7.79 | 6.9 | 0.461 | 0.03 | 0.1 | 0.591 | 0.02 | 2.9 | 2.77 | 2.42 | 0.977 | 0.04 |
| 18AU_05.2 | 26 | 23.35 | 7.79 | 6.9 | 0.461 | 0.03 | 0.1 | 0.591 | 0.02 | 2.9 | 2.77 | 2.42 | 0.977 | 0.04 |
| 18AU_05.3 | 26 | 23.35 | 7.79 | 6.9 | 0.461 | 0.03 | 0.1 | 0.591 | 0.02 | 2.9 | 2.77 | 2.42 | 0.977 | 0.04 |
| 18AU_05.4 | 26 | 23.35 | 7.79 | 6.9 | 0.461 | 0.03 | 0.1 | 0.591 | 0.02 | 2.9 | 2.77 | 2.42 | 0.977 | 0.04 |
| 18AU_05.5 | 26 | 23.35 | 7.79 | 6.9 | 0.461 | 0.03 | 0.1 | 0.591 | 0.02 | 2.9 | 2.77 | 2.42 | 0.977 | 0.04 |
| 18AU_06.1 | 26 | 26.05 | 7.93 | 6.9 | 0.529 | 0.034 | 0.085 | 0.648 | 0.016 | 2.4 | 2.79 | 1.9 | 0.967 | 0.038 |
| 18AU_06.2 | 26 | 26.05 | 7.93 | 6.9 | 0.529 | 0.034 | 0.085 | 0.648 | 0.016 | 2.4 | 2.79 | 1.9 | 0.967 | 0.038 |
| 18AU_06.3 | 26 | 26.05 | 7.93 | 6.9 | 0.529 | 0.034 | 0.085 | 0.648 | 0.016 | 2.4 | 2.79 | 1.9 | 0.967 | 0.038 |
| 18AU_06.4 | 26 | 26.05 | 7.93 | 6.9 | 0.529 | 0.034 | 0.085 | 0.648 | 0.016 | 2.4 | 2.79 | 1.9 | 0.967 | 0.038 |
| 18AU_06.5 | 26 | 26.05 | 7.93 | 6.9 | 0.529 | 0.034 | 0.085 | 0.648 | 0.016 | 2.4 | 2.79 | 1.9 | 0.967 | 0.038 |
| 18AU_07.1 | 27.1 | 27.24 | 7.97 | 7 | 0.468 | 0.023 | 0.069 | 0.56 | 0.02 | 2.5 | 2.42 | 2.06 | 0.952 | 0.042 |
| 18AU_07.2 | 27.1 | 27.24 | 7.97 | 7 | 0.468 | 0.023 | 0.069 | 0.56 | 0.02 | 2.5 | 2.42 | 2.06 | 0.952 | 0.042 |
| 18AU_07.3 | 27.1 | 27.24 | 7.97 | 7 | 0.468 | 0.023 | 0.069 | 0.56 | 0.02 | 2.5 | 2.42 | 2.06 | 0.952 | 0.042 |
| 18AU_07.4 | 27.1 | 27.24 | 7.97 | 7 | 0.468 | 0.023 | 0.069 | 0.56 | 0.02 | 2.5 | 2.42 | 2.06 | 0.952 | 0.042 |
| 18AU_07.5 | 27.1 | 27.24 | 7.97 | 7 | 0.468 | 0.023 | 0.069 | 0.56 | 0.02 | 2.5 | 2.42 | 2.06 | 0.952 | 0.042 |
| 18AU_09.1 | 27.6 | 28.32 | 8.07 | 6.9 | 0.331 | 0.006 | 0.074 | 0.411 | 0.025 | 3.1 | 2.59 | 1.66 | 0.567 | 0.057 |
| 18AU_09.2 | 27.6 | 28.32 | 8.07 | 6.9 | 0.331 | 0.006 | 0.074 | 0.411 | 0.025 | 3.1 | 2.59 | 1.66 | 0.567 | 0.057 |
| 18AU_09.3 | 27.6 | 28.32 | 8.07 | 6.9 | 0.331 | 0.006 | 0.074 | 0.411 | 0.025 | 3.1 | 2.59 | 1.66 | 0.567 | 0.057 |
| 18AU_09.4 | 27.6 | 28.32 | 8.07 | 6.9 | 0.331 | 0.006 | 0.074 | 0.411 | 0.025 | 3.1 | 2.59 | 1.66 | 0.567 | 0.057 |
| 18AU_09.5 | 27.6 | 28.32 | 8.07 | 6.9 | 0.331 | 0.006 | 0.074 | 0.411 | 0.025 | 3.1 | 2.59 | 1.66 | 0.567 | 0.057 |
| 18AU_10.1 | 27.3 | 27.68 | 8.05 | 7.1 | 0.235 | 0.018 | 0.088 | 0.341 | 0.021 | 4.1 | 2.42 | 1.7 | 0.442 | 0.049 |
| 18AU_10.2 | 27.3 | 27.68 | 8.05 | 7.1 | 0.235 | 0.018 | 0.088 | 0.341 | 0.021 | 4.1 | 2.42 | 1.7 | 0.442 | 0.049 |
| 18AU_10.3 | 27.3 | 27.68 | 8.05 | 7.1 | 0.235 | 0.018 | 0.088 | 0.341 | 0.021 | 4.1 | 2.42 | 1.7 | 0.442 | 0.049 |
| 18AU_10.4 | 27.3 | 27.68 | 8.05 | 7.1 | 0.235 | 0.018 | 0.088 | 0.341 | 0.021 | 4.1 | 2.42 | 1.7 | 0.442 | 0.049 |
| 18AU_10.5 | 27.3 | 27.68 | 8.05 | 7.1 | 0.235 | 0.018 | 0.088 | 0.341 | 0.021 | 4.1 | 2.42 | 1.7 | 0.442 | 0.049 |
| 18AU_11.1 | 27.5 | 31.84 | 8.05 | 7.2 | 0.279 | 0.005 | 0.086 | 0.37 | 0.018 | 2 | 2.2 | 1.37 | 0.44 | 0.045 |
| 18AU_11.2 | 27.5 | 31.84 | 8.05 | 7.2 | 0.279 | 0.005 | 0.086 | 0.37 | 0.018 | 2 | 2.2 | 1.37 | 0.44 | 0.045 |
| 18AU_11.3 | 27.5 | 31.84 | 8.05 | 7.2 | 0.279 | 0.005 | 0.086 | 0.37 | 0.018 | 2 | 2.2 | 1.37 | 0.44 | 0.045 |
| 18AU_11.4 | 27.5 | 31.84 | 8.05 | 7.2 | 0.279 | 0.005 | 0.086 | 0.37 | 0.018 | 2 | 2.2 | 1.37 | 0.44 | 0.045 |
| 18AU_11.5 | 27.5 | 31.84 | 8.05 | 7.2 | 0.279 | 0.005 | 0.086 | 0.37 | 0.018 | 2 | 2.2 | 1.37 | 0.44 | 0.045 |
| 18AU_12.1 | 27 | 31.06 | 8.21 | 7.1 | 0.29 | 0.007 | 0.069 | 0.366 | 0.011 | 2.2 | 2.45 | 1.53 | 0.604 | 0.027 |
| 18AU_12.2 | 27 | 31.06 | 8.21 | 7.1 | 0.29 | 0.007 | 0.069 | 0.366 | 0.011 | 2.2 | 2.45 | 1.53 | 0.604 | 0.027 |
| 18AU_12.3 | 27 | 31.06 | 8.21 | 7.1 | 0.29 | 0.007 | 0.069 | 0.366 | 0.011 | 2.2 | 2.45 | 1.53 | 0.604 | 0.027 |
| 18AU_12.4 | 27 | 31.06 | 8.21 | 7.1 | 0.29 | 0.007 | 0.069 | 0.366 | 0.011 | 2.2 | 2.45 | 1.53 | 0.604 | 0.027 |
| 18AU_12.5 | 27 | 31.06 | 8.21 | 7.1 | 0.29 | 0.007 | 0.069 | 0.366 | 0.011 | 2.2 | 2.45 | 1.53 | 0.604 | 0.027 |
| 18AU_13.1 | 27.2 | 32.42 | 8.05 | 7 | 0.229 | 0.008 | 0.063 | 0.3 | 0.017 | 2 | 2.29 | 1.35 | 0.569 | 0.033 |
| 18AU_13.2 | 27.2 | 32.42 | 8.05 | 7 | 0.229 | 0.008 | 0.063 | 0.3 | 0.017 | 2 | 2.29 | 1.35 | 0.569 | 0.033 |
| 18AU_13.3 | 27.2 | 32.42 | 8.05 | 7 | 0.229 | 0.008 | 0.063 | 0.3 | 0.017 | 2 | 2.29 | 1.35 | 0.569 | 0.033 |
| 18AU_13.4 | 27.2 | 32.42 | 8.05 | 7 | 0.229 | 0.008 | 0.063 | 0.3 | 0.017 | 2 | 2.29 | 1.35 | 0.569 | 0.033 |
| 18AU_13.5 | 27.2 | 32.42 | 8.05 | 7 | 0.229 | 0.008 | 0.063 | 0.3 | 0.017 | 2 | 2.29 | 1.35 | 0.569 | 0.033 |
| 18AU_14.1 | 27.3 | 32.3 | 7.98 | 7.1 | 0.176 | 0.008 | 0.053 | 0.237 | 0.018 | 2.4 | 2.03 | 1.52 | 0.559 | 0.035 |
| 18AU_14.2 | 27.3 | 32.3 | 7.98 | 7.1 | 0.176 | 0.008 | 0.053 | 0.237 | 0.018 | 2.4 | 2.03 | 1.52 | 0.559 | 0.035 |
| 18AU_14.3 | 27.3 | 32.3 | 7.98 | 7.1 | 0.176 | 0.008 | 0.053 | 0.237 | 0.018 | 2.4 | 2.03 | 1.52 | 0.559 | 0.035 |
| 18AU_14.4 | 27.3 | 32.3 | 7.98 | 7.1 | 0.176 | 0.008 | 0.053 | 0.237 | 0.018 | 2.4 | 2.03 | 1.52 | 0.559 | 0.035 |
| 18AU_14.5 | 27.3 | 32.3 | 7.98 | 7.1 | 0.176 | 0.008 | 0.053 | 0.237 | 0.018 | 2.4 | 2.03 | 1.52 | 0.559 | 0.035 |
| 18AU_15.1 | 26.8 | 33.63 | 7.97 | 6.5 | 0.157 | 0.007 | 0.025 | 0.189 | 0.021 | 1.8 | 2.21 | 1.48 | 0.722 | 0.038 |
| 18AU_15.2 | 26.8 | 33.63 | 7.97 | 6.5 | 0.157 | 0.007 | 0.025 | 0.189 | 0.021 | 1.8 | 2.21 | 1.48 | 0.722 | 0.038 |
| 18AU_15.3 | 26.8 | 33.63 | 7.97 | 6.5 | 0.157 | 0.007 | 0.025 | 0.189 | 0.021 | 1.8 | 2.21 | 1.48 | 0.722 | 0.038 |
| 18AU_15.4 | 26.8 | 33.63 | 7.97 | 6.5 | 0.157 | 0.007 | 0.025 | 0.189 | 0.021 | 1.8 | 2.21 | 1.48 | 0.722 | 0.038 |
| 18AU_15.5 | 26.8 | 33.63 | 7.97 | 6.5 | 0.157 | 0.007 | 0.025 | 0.189 | 0.021 | 1.8 | 2.21 | 1.48 | 0.722 | 0.038 |
| 18AU_16.1 | 27.1 | 22.16 | 8.08 | 6.8 | 0.124 | 0.01 | 0.04 | 0.174 | 0.027 | 3.8 | 2.44 | 1.21 | 0.356 | 0.05 |
| 18AU_16.2 | 27.1 | 22.16 | 8.08 | 6.8 | 0.124 | 0.01 | 0.04 | 0.174 | 0.027 | 3.8 | 2.44 | 1.21 | 0.356 | 0.05 |
| 18AU_16.3 | 27.1 | 22.16 | 8.08 | 6.8 | 0.124 | 0.01 | 0.04 | 0.174 | 0.027 | 3.8 | 2.44 | 1.21 | 0.356 | 0.05 |
| 18AU_16.4 | 27.1 | 22.16 | 8.08 | 6.8 | 0.124 | 0.01 | 0.04 | 0.174 | 0.027 | 3.8 | 2.44 | 1.21 | 0.356 | 0.05 |
| 18AU_16.5 | 27.1 | 22.16 | 8.08 | 6.8 | 0.124 | 0.01 | 0.04 | 0.174 | 0.027 | 3.8 | 2.44 | 1.21 | 0.356 | 0.05 |
| 18SP_01.1 | 25.47 | 21.6 | 10.34 | 6.7 | 0.799 | 0.018 | 0.109 | 0.926 | 0.028 | 9.3 | 3.28 | 2.76 | 1.481 | 0.067 |
| 18SP_01.2 | 25.47 | 21.6 | 10.34 | 6.7 | 0.799 | 0.018 | 0.109 | 0.926 | 0.028 | 9.3 | 3.28 | 2.76 | 1.481 | 0.067 |
| 18SP_01.3 | 25.47 | 21.6 | 10.34 | 6.7 | 0.799 | 0.018 | 0.109 | 0.926 | 0.028 | 9.3 | 3.28 | 2.76 | 1.481 | 0.067 |
| 18SP_01.4 | 25.47 | 21.6 | 10.34 | 6.7 | 0.799 | 0.018 | 0.109 | 0.926 | 0.028 | 9.3 | 3.28 | 2.76 | 1.481 | 0.067 |
| 18SP_01.5 | 25.47 | 21.6 | 10.34 | 6.7 | 0.799 | 0.018 | 0.109 | 0.926 | 0.028 | 9.3 | 3.28 | 2.76 | 1.481 | 0.067 |
| 18SP_02.1 | 24.84 | 21.02 | 10.41 | 6.5 | 0.824 | 0.017 | 0.097 | 0.938 | 0.018 | 10.6 | 3.2 | 2.73 | 1.78 | 0.037 |
| 18SP_02.2 | 24.84 | 21.02 | 10.41 | 6.5 | 0.824 | 0.017 | 0.097 | 0.938 | 0.018 | 10.6 | 3.2 | 2.73 | 1.78 | 0.037 |
| 18SP_02.3 | 24.84 | 21.02 | 10.41 | 6.5 | 0.824 | 0.017 | 0.097 | 0.938 | 0.018 | 10.6 | 3.2 | 2.73 | 1.78 | 0.037 |
| 18SP_02.4 | 24.84 | 21.02 | 10.41 | 6.5 | 0.824 | 0.017 | 0.097 | 0.938 | 0.018 | 10.6 | 3.2 | 2.73 | 1.78 | 0.037 |
| 18SP_02.5 | 24.84 | 21.02 | 10.41 | 6.5 | 0.824 | 0.017 | 0.097 | 0.938 | 0.018 | 10.6 | 3.2 | 2.73 | 1.78 | 0.037 |
| 18SP_03.1 | 24.9 | 22.64 | 10.1 | 6.3 | 0.597 | 0.012 | 0.062 | 0.671 | 0.017 | 5.9 | 2.85 | 2.52 | 1.265 | 0.038 |
| 18SP_03.2 | 24.9 | 22.64 | 10.1 | 6.3 | 0.597 | 0.012 | 0.062 | 0.671 | 0.017 | 5.9 | 2.85 | 2.52 | 1.265 | 0.038 |
| 18SP_03.3 | 24.9 | 22.64 | 10.1 | 6.3 | 0.597 | 0.012 | 0.062 | 0.671 | 0.017 | 5.9 | 2.85 | 2.52 | 1.265 | 0.038 |
| 18SP_03.4 | 24.9 | 22.64 | 10.1 | 6.3 | 0.597 | 0.012 | 0.062 | 0.671 | 0.017 | 5.9 | 2.85 | 2.52 | 1.265 | 0.038 |
| 18SP_03.5 | 24.9 | 22.64 | 10.1 | 6.3 | 0.597 | 0.012 | 0.062 | 0.671 | 0.017 | 5.9 | 2.85 | 2.52 | 1.265 | 0.038 |
| 18SP_04.1 | 24.37 | 23.16 | 10 | 6.3 | 0.387 | 0.008 | 0.058 | 0.453 | 0.012 | 4.2 | 3.19 | 2.64 | 0.905 | 0.026 |
| 18SP_04.2 | 24.37 | 23.16 | 10 | 6.3 | 0.387 | 0.008 | 0.058 | 0.453 | 0.012 | 4.2 | 3.19 | 2.64 | 0.905 | 0.026 |
| 18SP_04.3 | 24.37 | 23.16 | 10 | 6.3 | 0.387 | 0.008 | 0.058 | 0.453 | 0.012 | 4.2 | 3.19 | 2.64 | 0.905 | 0.026 |
| 18SP_04.4 | 24.37 | 23.16 | 10 | 6.3 | 0.387 | 0.008 | 0.058 | 0.453 | 0.012 | 4.2 | 3.19 | 2.64 | 0.905 | 0.026 |
| 18SP_04.5 | 24.37 | 23.16 | 10 | 6.3 | 0.387 | 0.008 | 0.058 | 0.453 | 0.012 | 4.2 | 3.19 | 2.64 | 0.905 | 0.026 |
| 18SP_05.1 | 24.75 | 23.73 | 10.2 | 6.5 | 0.395 | 0.009 | 0.06 | 0.464 | 0.013 | 3.1 | 2.89 | 2.54 | 0.698 | 0.029 |
| 18SP_05.2 | 24.75 | 23.73 | 10.2 | 6.5 | 0.395 | 0.009 | 0.06 | 0.464 | 0.013 | 3.1 | 2.89 | 2.54 | 0.698 | 0.029 |
| 18SP_05.3 | 24.75 | 23.73 | 10.2 | 6.5 | 0.395 | 0.009 | 0.06 | 0.464 | 0.013 | 3.1 | 2.89 | 2.54 | 0.698 | 0.029 |
| 18SP_05.4 | 24.75 | 23.73 | 10.2 | 6.5 | 0.395 | 0.009 | 0.06 | 0.464 | 0.013 | 3.1 | 2.89 | 2.54 | 0.698 | 0.029 |
| 18SP_05.5 | 24.75 | 23.73 | 10.2 | 6.5 | 0.395 | 0.009 | 0.06 | 0.464 | 0.013 | 3.1 | 2.89 | 2.54 | 0.698 | 0.029 |
| 18SP_06.1 | 24.46 | 25.17 | 10.94 | 6.7 | 0.343 | 0.008 | 0.059 | 0.41 | 0.012 | 4.5 | 2.69 | 2.46 | 0.564 | 0.029 |
| 18SP_06.2 | 24.46 | 25.17 | 10.94 | 6.7 | 0.343 | 0.008 | 0.059 | 0.41 | 0.012 | 4.5 | 2.69 | 2.46 | 0.564 | 0.029 |
| 18SP_06.3 | 24.46 | 25.17 | 10.94 | 6.7 | 0.343 | 0.008 | 0.059 | 0.41 | 0.012 | 4.5 | 2.69 | 2.46 | 0.564 | 0.029 |
| 18SP_06.4 | 24.46 | 25.17 | 10.94 | 6.7 | 0.343 | 0.008 | 0.059 | 0.41 | 0.012 | 4.5 | 2.69 | 2.46 | 0.564 | 0.029 |
| 18SP_06.5 | 24.46 | 25.17 | 10.94 | 6.7 | 0.343 | 0.008 | 0.059 | 0.41 | 0.012 | 4.5 | 2.69 | 2.46 | 0.564 | 0.029 |
| 18SP_07.1 | 24.15 | 26.24 | 10.5 | 6.7 | 0.328 | 0.007 | 0.049 | 0.384 | 0.012 | 4.9 | 2.16 | 2.35 | 0.469 | 0.029 |
| 18SP_07.2 | 24.15 | 26.24 | 10.5 | 6.7 | 0.328 | 0.007 | 0.049 | 0.384 | 0.012 | 4.9 | 2.16 | 2.35 | 0.469 | 0.029 |
| 18SP_07.3 | 24.15 | 26.24 | 10.5 | 6.7 | 0.328 | 0.007 | 0.049 | 0.384 | 0.012 | 4.9 | 2.16 | 2.35 | 0.469 | 0.029 |
| 18SP_07.4 | 24.15 | 26.24 | 10.5 | 6.7 | 0.328 | 0.007 | 0.049 | 0.384 | 0.012 | 4.9 | 2.16 | 2.35 | 0.469 | 0.029 |
| 18SP_07.5 | 24.15 | 26.24 | 10.5 | 6.7 | 0.328 | 0.007 | 0.049 | 0.384 | 0.012 | 4.9 | 2.16 | 2.35 | 0.469 | 0.029 |
| 18SP_09.1 | 25.6 | 26.9 | 9.11 | 7.2 | 0.176 | 0.008 | 0.035 | 0.219 | 0.028 | 6.7 | 2.46 | 2.18 | 0.303 | 0.055 |
| 18SP_09.2 | 25.6 | 26.9 | 9.11 | 7.2 | 0.176 | 0.008 | 0.035 | 0.219 | 0.028 | 6.7 | 2.46 | 2.18 | 0.303 | 0.055 |
| 18SP_09.3 | 25.6 | 26.9 | 9.11 | 7.2 | 0.176 | 0.008 | 0.035 | 0.219 | 0.028 | 6.7 | 2.46 | 2.18 | 0.303 | 0.055 |
| 18SP_09.4 | 25.6 | 26.9 | 9.11 | 7.2 | 0.176 | 0.008 | 0.035 | 0.219 | 0.028 | 6.7 | 2.46 | 2.18 | 0.303 | 0.055 |
| 18SP_09.5 | 25.6 | 26.9 | 9.11 | 7.2 | 0.176 | 0.008 | 0.035 | 0.219 | 0.028 | 6.7 | 2.46 | 2.18 | 0.303 | 0.055 |
| 18SP_10.1 | 26.6 | 26.76 | 9.19 | 7.6 | 0.129 | 0.008 | 0.025 | 0.162 | 0.038 | 8.7 | 1.93 | 2.14 | 0.266 | 0.07 |
| 18SP_10.2 | 26.6 | 26.76 | 9.19 | 7.6 | 0.129 | 0.008 | 0.025 | 0.162 | 0.038 | 8.7 | 1.93 | 2.14 | 0.266 | 0.07 |
| 18SP_10.3 | 26.6 | 26.76 | 9.19 | 7.6 | 0.129 | 0.008 | 0.025 | 0.162 | 0.038 | 8.7 | 1.93 | 2.14 | 0.266 | 0.07 |
| 18SP_10.4 | 26.6 | 26.76 | 9.19 | 7.6 | 0.129 | 0.008 | 0.025 | 0.162 | 0.038 | 8.7 | 1.93 | 2.14 | 0.266 | 0.07 |
| 18SP_10.5 | 26.6 | 26.76 | 9.19 | 7.6 | 0.129 | 0.008 | 0.025 | 0.162 | 0.038 | 8.7 | 1.93 | 2.14 | 0.266 | 0.07 |
| 18SP_11.1 | 24.6 | 29.44 | 9.6 | 7.1 | 0.147 | 0.005 | 0.026 | 0.178 | 0.029 | 7.4 | 2.28 | 1.94 | 0.241 | 0.062 |
| 18SP_11.2 | 24.6 | 29.44 | 9.6 | 7.1 | 0.147 | 0.005 | 0.026 | 0.178 | 0.029 | 7.4 | 2.28 | 1.94 | 0.241 | 0.062 |
| 18SP_11.3 | 24.6 | 29.44 | 9.6 | 7.1 | 0.147 | 0.005 | 0.026 | 0.178 | 0.029 | 7.4 | 2.28 | 1.94 | 0.241 | 0.062 |
| 18SP_11.4 | 24.6 | 29.44 | 9.6 | 7.1 | 0.147 | 0.005 | 0.026 | 0.178 | 0.029 | 7.4 | 2.28 | 1.94 | 0.241 | 0.062 |
| 18SP_11.5 | 24.6 | 29.44 | 9.6 | 7.1 | 0.147 | 0.005 | 0.026 | 0.178 | 0.029 | 7.4 | 2.28 | 1.94 | 0.241 | 0.062 |
| 18SP_12.1 | 23.8 | 29.85 | 9.63 | 6.9 | 0.173 | 0.006 | 0.024 | 0.203 | 0.019 | 3 | 1.97 | 1.43 | 0.28 | 0.044 |
| 18SP_12.2 | 23.8 | 29.85 | 9.63 | 6.9 | 0.173 | 0.006 | 0.024 | 0.203 | 0.019 | 3 | 1.97 | 1.43 | 0.28 | 0.044 |
| 18SP_12.3 | 23.8 | 29.85 | 9.63 | 6.9 | 0.173 | 0.006 | 0.024 | 0.203 | 0.019 | 3 | 1.97 | 1.43 | 0.28 | 0.044 |
| 18SP_12.4 | 23.8 | 29.85 | 9.63 | 6.9 | 0.173 | 0.006 | 0.024 | 0.203 | 0.019 | 3 | 1.97 | 1.43 | 0.28 | 0.044 |
| 18SP_12.5 | 23.8 | 29.85 | 9.63 | 6.9 | 0.173 | 0.006 | 0.024 | 0.203 | 0.019 | 3 | 1.97 | 1.43 | 0.28 | 0.044 |
| 18SP_13.1 | 25.08 | 29.45 | 10.24 | 7.1 | 0.169 | 0.006 | 0.04 | 0.215 | 0.022 | 4.9 | 2.02 | 1.24 | 0.29 | 0.053 |
| 18SP_13.2 | 25.08 | 29.45 | 10.24 | 7.1 | 0.169 | 0.006 | 0.04 | 0.215 | 0.022 | 4.9 | 2.02 | 1.24 | 0.29 | 0.053 |
| 18SP_13.3 | 25.08 | 29.45 | 10.24 | 7.1 | 0.169 | 0.006 | 0.04 | 0.215 | 0.022 | 4.9 | 2.02 | 1.24 | 0.29 | 0.053 |
| 18SP_13.4 | 25.08 | 29.45 | 10.24 | 7.1 | 0.169 | 0.006 | 0.04 | 0.215 | 0.022 | 4.9 | 2.02 | 1.24 | 0.29 | 0.053 |
| 18SP_13.5 | 25.08 | 29.45 | 10.24 | 7.1 | 0.169 | 0.006 | 0.04 | 0.215 | 0.022 | 4.9 | 2.02 | 1.24 | 0.29 | 0.053 |
| 18SP_15.1 | 25.33 | 30.21 | 10.5 | 6.7 | 0.184 | 0.008 | 0.017 | 0.209 | 0.023 | 7.8 | 2.08 | 1.34 | 0.308 | 0.055 |
| 18SP_15.2 | 25.33 | 30.21 | 10.5 | 6.7 | 0.184 | 0.008 | 0.017 | 0.209 | 0.023 | 7.8 | 2.08 | 1.34 | 0.308 | 0.055 |
| 18SP_15.3 | 25.33 | 30.21 | 10.5 | 6.7 | 0.184 | 0.008 | 0.017 | 0.209 | 0.023 | 7.8 | 2.08 | 1.34 | 0.308 | 0.055 |
| 18SP_15.4 | 25.33 | 30.21 | 10.5 | 6.7 | 0.184 | 0.008 | 0.017 | 0.209 | 0.023 | 7.8 | 2.08 | 1.34 | 0.308 | 0.055 |
| 18SP_15.5 | 25.33 | 30.21 | 10.5 | 6.7 | 0.184 | 0.008 | 0.017 | 0.209 | 0.023 | 7.8 | 2.08 | 1.34 | 0.308 | 0.055 |
| 18SP_16.1 | 25.6 | 30.23 | 9.87 | 6.8 | 0.142 | 0.005 | 0.035 | 0.182 | 0.023 | 6.1 | 2.03 | 1.26 | 0.267 | 0.055 |
| 18SP_16.2 | 25.6 | 30.23 | 9.87 | 6.8 | 0.142 | 0.005 | 0.035 | 0.182 | 0.023 | 6.1 | 2.03 | 1.26 | 0.267 | 0.055 |
| 18SP_16.3 | 25.6 | 30.23 | 9.87 | 6.8 | 0.142 | 0.005 | 0.035 | 0.182 | 0.023 | 6.1 | 2.03 | 1.26 | 0.267 | 0.055 |
| 18SP_16.4 | 25.6 | 30.23 | 9.87 | 6.8 | 0.142 | 0.005 | 0.035 | 0.182 | 0.023 | 6.1 | 2.03 | 1.26 | 0.267 | 0.055 |
| 18SP_16.5 | 25.6 | 30.23 | 9.87 | 6.8 | 0.142 | 0.005 | 0.035 | 0.182 | 0.023 | 6.1 | 2.03 | 1.26 | 0.267 | 0.055 |
| 18SU_01.1 | 29.77 | 17.35 | 9.89 | 5.2 | 0.598 | 0.204 | 0.073 | 0.875 | 0.028 | 3.6 | 4.27 | 2.48 | 1.148 | 0.054 |
| 18SU_01.2 | 29.77 | 17.35 | 9.89 | 5.2 | 0.598 | 0.204 | 0.073 | 0.875 | 0.028 | 3.6 | 4.27 | 2.48 | 1.148 | 0.054 |
| 18SU_01.3 | 29.77 | 17.35 | 9.89 | 5.2 | 0.598 | 0.204 | 0.073 | 0.875 | 0.028 | 3.6 | 4.27 | 2.48 | 1.148 | 0.054 |
| 18SU_01.4 | 29.77 | 17.35 | 9.89 | 5.2 | 0.598 | 0.204 | 0.073 | 0.875 | 0.028 | 3.6 | 4.27 | 2.48 | 1.148 | 0.054 |
| 18SU_01.5 | 29.77 | 17.35 | 9.89 | 5.2 | 0.598 | 0.204 | 0.073 | 0.875 | 0.028 | 3.6 | 4.27 | 2.48 | 1.148 | 0.054 |
| 18SU_02.1 | 29.69 | 17.72 | 9.85 | 5.5 | 0.617 | 0.08 | 0.067 | 0.764 | 0.025 | 3.6 | 4.03 | 2.65 | 0.935 | 0.047 |
| 18SU_02.2 | 29.69 | 17.72 | 9.85 | 5.5 | 0.617 | 0.08 | 0.067 | 0.764 | 0.025 | 3.6 | 4.03 | 2.65 | 0.935 | 0.047 |
| 18SU_02.3 | 29.69 | 17.72 | 9.85 | 5.5 | 0.617 | 0.08 | 0.067 | 0.764 | 0.025 | 3.6 | 4.03 | 2.65 | 0.935 | 0.047 |
| 18SU_02.4 | 29.69 | 17.72 | 9.85 | 5.5 | 0.617 | 0.08 | 0.067 | 0.764 | 0.025 | 3.6 | 4.03 | 2.65 | 0.935 | 0.047 |
| 18SU_02.5 | 29.69 | 17.72 | 9.85 | 5.5 | 0.617 | 0.08 | 0.067 | 0.764 | 0.025 | 3.6 | 4.03 | 2.65 | 0.935 | 0.047 |
| 18SU_03.1 | 29.65 | 18.83 | 9.67 | 5.4 | 0.672 | 0.182 | 0.099 | 0.953 | 0.021 | 4.4 | 3.78 | 2.48 | 1.056 | 0.04 |
| 18SU_03.2 | 29.65 | 18.83 | 9.67 | 5.4 | 0.672 | 0.182 | 0.099 | 0.953 | 0.021 | 4.4 | 3.78 | 2.48 | 1.056 | 0.04 |
| 18SU_03.3 | 29.65 | 18.83 | 9.67 | 5.4 | 0.672 | 0.182 | 0.099 | 0.953 | 0.021 | 4.4 | 3.78 | 2.48 | 1.056 | 0.04 |
| 18SU_03.4 | 29.65 | 18.83 | 9.67 | 5.4 | 0.672 | 0.182 | 0.099 | 0.953 | 0.021 | 4.4 | 3.78 | 2.48 | 1.056 | 0.04 |
| 18SU_03.5 | 29.65 | 18.83 | 9.67 | 5.4 | 0.672 | 0.182 | 0.099 | 0.953 | 0.021 | 4.4 | 3.78 | 2.48 | 1.056 | 0.04 |
| 18SU_04.1 | 29.67 | 18.76 | 10.05 | 5.4 | 0.48 | 0.162 | 0.06 | 0.702 | 0.033 | 5.8 | 2.92 | 2.26 | 0.822 | 0.082 |
| 18SU_04.2 | 29.67 | 18.76 | 10.05 | 5.4 | 0.48 | 0.162 | 0.06 | 0.702 | 0.033 | 5.8 | 2.92 | 2.26 | 0.822 | 0.082 |
| 18SU_04.3 | 29.67 | 18.76 | 10.05 | 5.4 | 0.48 | 0.162 | 0.06 | 0.702 | 0.033 | 5.8 | 2.92 | 2.26 | 0.822 | 0.082 |
| 18SU_04.4 | 29.67 | 18.76 | 10.05 | 5.4 | 0.48 | 0.162 | 0.06 | 0.702 | 0.033 | 5.8 | 2.92 | 2.26 | 0.822 | 0.082 |
| 18SU_04.5 | 29.67 | 18.76 | 10.05 | 5.4 | 0.48 | 0.162 | 0.06 | 0.702 | 0.033 | 5.8 | 2.92 | 2.26 | 0.822 | 0.082 |
| 18SU_05.1 | 29.56 | 19.1 | 9.75 | 5.6 | 0.504 | 0.12 | 0.057 | 0.681 | 0.028 | 3.3 | 2.49 | 2.47 | 0.812 | 0.064 |
| 18SU_05.2 | 29.56 | 19.1 | 9.75 | 5.6 | 0.504 | 0.12 | 0.057 | 0.681 | 0.028 | 3.3 | 2.49 | 2.47 | 0.812 | 0.064 |
| 18SU_05.3 | 29.56 | 19.1 | 9.75 | 5.6 | 0.504 | 0.12 | 0.057 | 0.681 | 0.028 | 3.3 | 2.49 | 2.47 | 0.812 | 0.064 |
| 18SU_05.4 | 29.56 | 19.1 | 9.75 | 5.6 | 0.504 | 0.12 | 0.057 | 0.681 | 0.028 | 3.3 | 2.49 | 2.47 | 0.812 | 0.064 |
| 18SU_05.5 | 29.56 | 19.1 | 9.75 | 5.6 | 0.504 | 0.12 | 0.057 | 0.681 | 0.028 | 3.3 | 2.49 | 2.47 | 0.812 | 0.064 |
| 18SU_06.1 | 29.3 | 24.21 | 10.08 | 5.8 | 0.336 | 0.104 | 0.06 | 0.5 | 0.038 | 5.1 | 2.71 | 2.57 | 0.663 | 0.082 |
| 18SU_06.2 | 29.3 | 24.21 | 10.08 | 5.8 | 0.336 | 0.104 | 0.06 | 0.5 | 0.038 | 5.1 | 2.71 | 2.57 | 0.663 | 0.082 |
| 18SU_06.3 | 29.3 | 24.21 | 10.08 | 5.8 | 0.336 | 0.104 | 0.06 | 0.5 | 0.038 | 5.1 | 2.71 | 2.57 | 0.663 | 0.082 |
| 18SU_06.4 | 29.3 | 24.21 | 10.08 | 5.8 | 0.336 | 0.104 | 0.06 | 0.5 | 0.038 | 5.1 | 2.71 | 2.57 | 0.663 | 0.082 |
| 18SU_06.5 | 29.3 | 24.21 | 10.08 | 5.8 | 0.336 | 0.104 | 0.06 | 0.5 | 0.038 | 5.1 | 2.71 | 2.57 | 0.663 | 0.082 |
| 18SU_07.1 | 29.28 | 24.79 | 10.4 | 5.8 | 0.342 | 0.093 | 0.068 | 0.503 | 0.031 | 5.1 | 2.47 | 2.27 | 0.706 | 0.068 |
| 18SU_07.2 | 29.28 | 24.79 | 10.4 | 5.8 | 0.342 | 0.093 | 0.068 | 0.503 | 0.031 | 5.1 | 2.47 | 2.27 | 0.706 | 0.068 |
| 18SU_07.3 | 29.28 | 24.79 | 10.4 | 5.8 | 0.342 | 0.093 | 0.068 | 0.503 | 0.031 | 5.1 | 2.47 | 2.27 | 0.706 | 0.068 |
| 18SU_07.4 | 29.28 | 24.79 | 10.4 | 5.8 | 0.342 | 0.093 | 0.068 | 0.503 | 0.031 | 5.1 | 2.47 | 2.27 | 0.706 | 0.068 |
| 18SU_07.5 | 29.28 | 24.79 | 10.4 | 5.8 | 0.342 | 0.093 | 0.068 | 0.503 | 0.031 | 5.1 | 2.47 | 2.27 | 0.706 | 0.068 |
| 18SU_09.1 | 29.28 | 26 | 10.03 | 6.5 | 0.324 | 0.014 | 0.069 | 0.407 | 0.034 | 4.5 | 2.33 | 2 | 0.631 | 0.082 |
| 18SU_09.2 | 29.28 | 26 | 10.03 | 6.5 | 0.324 | 0.014 | 0.069 | 0.407 | 0.034 | 4.5 | 2.33 | 2 | 0.631 | 0.082 |
| 18SU_09.3 | 29.28 | 26 | 10.03 | 6.5 | 0.324 | 0.014 | 0.069 | 0.407 | 0.034 | 4.5 | 2.33 | 2 | 0.631 | 0.082 |
| 18SU_09.4 | 29.28 | 26 | 10.03 | 6.5 | 0.324 | 0.014 | 0.069 | 0.407 | 0.034 | 4.5 | 2.33 | 2 | 0.631 | 0.082 |
| 18SU_09.5 | 29.28 | 26 | 10.03 | 6.5 | 0.324 | 0.014 | 0.069 | 0.407 | 0.034 | 4.5 | 2.33 | 2 | 0.631 | 0.082 |
| 18SU_10.1 | 29.37 | 26.95 | 10.29 | 6.4 | 0.296 | 0.012 | 0.067 | 0.375 | 0.028 | 5.2 | 2.54 | 2.16 | 0.532 | 0.07 |
| 18SU_10.2 | 29.37 | 26.95 | 10.29 | 6.4 | 0.296 | 0.012 | 0.067 | 0.375 | 0.028 | 5.2 | 2.54 | 2.16 | 0.532 | 0.07 |
| 18SU_10.3 | 29.37 | 26.95 | 10.29 | 6.4 | 0.296 | 0.012 | 0.067 | 0.375 | 0.028 | 5.2 | 2.54 | 2.16 | 0.532 | 0.07 |
| 18SU_10.4 | 29.37 | 26.95 | 10.29 | 6.4 | 0.296 | 0.012 | 0.067 | 0.375 | 0.028 | 5.2 | 2.54 | 2.16 | 0.532 | 0.07 |
| 18SU_10.5 | 29.37 | 26.95 | 10.29 | 6.4 | 0.296 | 0.012 | 0.067 | 0.375 | 0.028 | 5.2 | 2.54 | 2.16 | 0.532 | 0.07 |
| 18SU_11.1 | 29.22 | 28.5 | 9.77 | 6.2 | 0.312 | 0.023 | 0.07 | 0.405 | 0.027 | 3.7 | 2.16 | 2.13 | 0.501 | 0.049 |
| 18SU_11.2 | 29.22 | 28.5 | 9.77 | 6.2 | 0.312 | 0.023 | 0.07 | 0.405 | 0.027 | 3.7 | 2.16 | 2.13 | 0.501 | 0.049 |
| 18SU_11.3 | 29.22 | 28.5 | 9.77 | 6.2 | 0.312 | 0.023 | 0.07 | 0.405 | 0.027 | 3.7 | 2.16 | 2.13 | 0.501 | 0.049 |
| 18SU_11.4 | 29.22 | 28.5 | 9.77 | 6.2 | 0.312 | 0.023 | 0.07 | 0.405 | 0.027 | 3.7 | 2.16 | 2.13 | 0.501 | 0.049 |
| 18SU_11.5 | 29.22 | 28.5 | 9.77 | 6.2 | 0.312 | 0.023 | 0.07 | 0.405 | 0.027 | 3.7 | 2.16 | 2.13 | 0.501 | 0.049 |
| 18SU_12.1 | 29.38 | 29.36 | 10.29 | 6.1 | 0.262 | 0.013 | 0.068 | 0.343 | 0.027 | 2.4 | 2.4 | 1.82 | 0.562 | 0.066 |
| 18SU_12.2 | 29.38 | 29.36 | 10.29 | 6.1 | 0.262 | 0.013 | 0.068 | 0.343 | 0.027 | 2.4 | 2.4 | 1.82 | 0.562 | 0.066 |
| 18SU_12.3 | 29.38 | 29.36 | 10.29 | 6.1 | 0.262 | 0.013 | 0.068 | 0.343 | 0.027 | 2.4 | 2.4 | 1.82 | 0.562 | 0.066 |
| 18SU_12.4 | 29.38 | 29.36 | 10.29 | 6.1 | 0.262 | 0.013 | 0.068 | 0.343 | 0.027 | 2.4 | 2.4 | 1.82 | 0.562 | 0.066 |
| 18SU_12.5 | 29.38 | 29.36 | 10.29 | 6.1 | 0.262 | 0.013 | 0.068 | 0.343 | 0.027 | 2.4 | 2.4 | 1.82 | 0.562 | 0.066 |
| 18SU_13.1 | 29.46 | 29.68 | 11.08 | 6.7 | 0.126 | 0.009 | 0.046 | 0.181 | 0.027 | 3.7 | 2.31 | 1.56 | 0.35 | 0.058 |
| 18SU_13.2 | 29.46 | 29.68 | 11.08 | 6.7 | 0.126 | 0.009 | 0.046 | 0.181 | 0.027 | 3.7 | 2.31 | 1.56 | 0.35 | 0.058 |
| 18SU_13.3 | 29.46 | 29.68 | 11.08 | 6.7 | 0.126 | 0.009 | 0.046 | 0.181 | 0.027 | 3.7 | 2.31 | 1.56 | 0.35 | 0.058 |
| 18SU_13.4 | 29.46 | 29.68 | 11.08 | 6.7 | 0.126 | 0.009 | 0.046 | 0.181 | 0.027 | 3.7 | 2.31 | 1.56 | 0.35 | 0.058 |
| 18SU_13.5 | 29.46 | 29.68 | 11.08 | 6.7 | 0.126 | 0.009 | 0.046 | 0.181 | 0.027 | 3.7 | 2.31 | 1.56 | 0.35 | 0.058 |
| 18SU_14.1 | 29.16 | 30.89 | 10.24 | 6.4 | 0.107 | 0.016 | 0.049 | 0.172 | 0.026 | 4.9 | 1.89 | 1.65 | 0.331 | 0.055 |
| 18SU_14.2 | 29.16 | 30.89 | 10.24 | 6.4 | 0.107 | 0.016 | 0.049 | 0.172 | 0.026 | 4.9 | 1.89 | 1.65 | 0.331 | 0.055 |
| 18SU_14.3 | 29.16 | 30.89 | 10.24 | 6.4 | 0.107 | 0.016 | 0.049 | 0.172 | 0.026 | 4.9 | 1.89 | 1.65 | 0.331 | 0.055 |
| 18SU_14.4 | 29.16 | 30.89 | 10.24 | 6.4 | 0.107 | 0.016 | 0.049 | 0.172 | 0.026 | 4.9 | 1.89 | 1.65 | 0.331 | 0.055 |
| 18SU_14.5 | 29.16 | 30.89 | 10.24 | 6.4 | 0.107 | 0.016 | 0.049 | 0.172 | 0.026 | 4.9 | 1.89 | 1.65 | 0.331 | 0.055 |
| 18SU_15.1 | 29.43 | 30.16 | 11.77 | 6.3 | 0.106 | 0.019 | 0.043 | 0.168 | 0.024 | 2.9 | 2.21 | 1.18 | 0.256 | 0.046 |
| 18SU_15.2 | 29.43 | 30.16 | 11.77 | 6.3 | 0.106 | 0.019 | 0.043 | 0.168 | 0.024 | 2.9 | 2.21 | 1.18 | 0.256 | 0.046 |
| 18SU_15.3 | 29.43 | 30.16 | 11.77 | 6.3 | 0.106 | 0.019 | 0.043 | 0.168 | 0.024 | 2.9 | 2.21 | 1.18 | 0.256 | 0.046 |
| 18SU_15.4 | 29.43 | 30.16 | 11.77 | 6.3 | 0.106 | 0.019 | 0.043 | 0.168 | 0.024 | 2.9 | 2.21 | 1.18 | 0.256 | 0.046 |
| 18SU_15.5 | 29.43 | 30.16 | 11.77 | 6.3 | 0.106 | 0.019 | 0.043 | 0.168 | 0.024 | 2.9 | 2.21 | 1.18 | 0.256 | 0.046 |
| 18SU_16.1 | 29.66 | 29.87 | 11.6 | 6.3 | 0.123 | 0.008 | 0.044 | 0.175 | 0.017 | 2.9 | 2.22 | 1.29 | 0.269 | 0.031 |
| 18SU_16.2 | 29.66 | 29.87 | 11.6 | 6.3 | 0.123 | 0.008 | 0.044 | 0.175 | 0.017 | 2.9 | 2.22 | 1.29 | 0.269 | 0.031 |
| 18SU_16.3 | 29.66 | 29.87 | 11.6 | 6.3 | 0.123 | 0.008 | 0.044 | 0.175 | 0.017 | 2.9 | 2.22 | 1.29 | 0.269 | 0.031 |
| 18SU_16.4 | 29.66 | 29.87 | 11.6 | 6.3 | 0.123 | 0.008 | 0.044 | 0.175 | 0.017 | 2.9 | 2.22 | 1.29 | 0.269 | 0.031 |
| 18SU_16.5 | 29.66 | 29.87 | 11.6 | 6.3 | 0.123 | 0.008 | 0.044 | 0.175 | 0.017 | 2.9 | 2.22 | 1.29 | 0.269 | 0.031 |
| 18WI_01.1 | 18.6 | 24.4 | 7.99 | 6.2 | 0.559 | 0.013 | 0.15 | 0.722 | 0.012 | 7.2 | 3.12 | 2.02 | 1.159 | 0.029 |
| 18WI_01.2 | 18.6 | 24.4 | 7.99 | 6.2 | 0.559 | 0.013 | 0.15 | 0.722 | 0.012 | 7.2 | 3.12 | 2.02 | 1.159 | 0.029 |
| 18WI_01.3 | 18.6 | 24.4 | 7.99 | 6.2 | 0.559 | 0.013 | 0.15 | 0.722 | 0.012 | 7.2 | 3.12 | 2.02 | 1.159 | 0.029 |
| 18WI_01.4 | 18.6 | 24.4 | 7.99 | 6.2 | 0.559 | 0.013 | 0.15 | 0.722 | 0.012 | 7.2 | 3.12 | 2.02 | 1.159 | 0.029 |
| 18WI_01.5 | 18.6 | 24.4 | 7.99 | 6.2 | 0.559 | 0.013 | 0.15 | 0.722 | 0.012 | 7.2 | 3.12 | 2.02 | 1.159 | 0.029 |
| 18WI_02.1 | 18.5 | 23.9 | 7.99 | 6.2 | 0.473 | 0.007 | 0.117 | 0.597 | 0.014 | 7.1 | 3.05 | 2.05 | 0.895 | 0.025 |
| 18WI_02.2 | 18.5 | 23.9 | 7.99 | 6.2 | 0.473 | 0.007 | 0.117 | 0.597 | 0.014 | 7.1 | 3.05 | 2.05 | 0.895 | 0.025 |
| 18WI_02.3 | 18.5 | 23.9 | 7.99 | 6.2 | 0.473 | 0.007 | 0.117 | 0.597 | 0.014 | 7.1 | 3.05 | 2.05 | 0.895 | 0.025 |
| 18WI_02.4 | 18.5 | 23.9 | 7.99 | 6.2 | 0.473 | 0.007 | 0.117 | 0.597 | 0.014 | 7.1 | 3.05 | 2.05 | 0.895 | 0.025 |
| 18WI_02.5 | 18.5 | 23.9 | 7.99 | 6.2 | 0.473 | 0.007 | 0.117 | 0.597 | 0.014 | 7.1 | 3.05 | 2.05 | 0.895 | 0.025 |
| 18WI_03.1 | 18.6 | 25.3 | 8.04 | 6.2 | 0.391 | 0.009 | 0.111 | 0.511 | 0.016 | 7.2 | 3.1 | 2.01 | 0.792 | 0.029 |
| 18WI_03.2 | 18.6 | 25.3 | 8.04 | 6.2 | 0.391 | 0.009 | 0.111 | 0.511 | 0.016 | 7.2 | 3.1 | 2.01 | 0.792 | 0.029 |
| 18WI_03.3 | 18.6 | 25.3 | 8.04 | 6.2 | 0.391 | 0.009 | 0.111 | 0.511 | 0.016 | 7.2 | 3.1 | 2.01 | 0.792 | 0.029 |
| 18WI_03.4 | 18.6 | 25.3 | 8.04 | 6.2 | 0.391 | 0.009 | 0.111 | 0.511 | 0.016 | 7.2 | 3.1 | 2.01 | 0.792 | 0.029 |
| 18WI_03.5 | 18.6 | 25.3 | 8.04 | 6.2 | 0.391 | 0.009 | 0.111 | 0.511 | 0.016 | 7.2 | 3.1 | 2.01 | 0.792 | 0.029 |
| 18WI_04.1 | 18.6 | 25.4 | 7.83 | 6.1 | 0.374 | 0.009 | 0.109 | 0.492 | 0.016 | 7.1 | 3.1 | 1.91 | 0.871 | 0.035 |
| 18WI_04.2 | 18.6 | 25.4 | 7.83 | 6.1 | 0.374 | 0.009 | 0.109 | 0.492 | 0.016 | 7.1 | 3.1 | 1.91 | 0.871 | 0.035 |
| 18WI_04.3 | 18.6 | 25.4 | 7.83 | 6.1 | 0.374 | 0.009 | 0.109 | 0.492 | 0.016 | 7.1 | 3.1 | 1.91 | 0.871 | 0.035 |
| 18WI_04.4 | 18.6 | 25.4 | 7.83 | 6.1 | 0.374 | 0.009 | 0.109 | 0.492 | 0.016 | 7.1 | 3.1 | 1.91 | 0.871 | 0.035 |
| 18WI_04.5 | 18.6 | 25.4 | 7.83 | 6.1 | 0.374 | 0.009 | 0.109 | 0.492 | 0.016 | 7.1 | 3.1 | 1.91 | 0.871 | 0.035 |
| 18WI_05.1 | 18.7 | 26 | 8.1 | 6.3 | 0.342 | 0.009 | 0.095 | 0.446 | 0.018 | 5.3 | 2.86 | 1.93 | 0.662 | 0.039 |
| 18WI_05.2 | 18.7 | 26 | 8.1 | 6.3 | 0.342 | 0.009 | 0.095 | 0.446 | 0.018 | 5.3 | 2.86 | 1.93 | 0.662 | 0.039 |
| 18WI_05.3 | 18.7 | 26 | 8.1 | 6.3 | 0.342 | 0.009 | 0.095 | 0.446 | 0.018 | 5.3 | 2.86 | 1.93 | 0.662 | 0.039 |
| 18WI_05.4 | 18.7 | 26 | 8.1 | 6.3 | 0.342 | 0.009 | 0.095 | 0.446 | 0.018 | 5.3 | 2.86 | 1.93 | 0.662 | 0.039 |
| 18WI_05.5 | 18.7 | 26 | 8.1 | 6.3 | 0.342 | 0.009 | 0.095 | 0.446 | 0.018 | 5.3 | 2.86 | 1.93 | 0.662 | 0.039 |
| 18WI_06.1 | 18.6 | 26.7 | 8.15 | 6.1 | 0.289 | 0.008 | 0.086 | 0.383 | 0.016 | 5 | 2.77 | 1.62 | 0.663 | 0.038 |
| 18WI_06.2 | 18.6 | 26.7 | 8.15 | 6.1 | 0.289 | 0.008 | 0.086 | 0.383 | 0.016 | 5 | 2.77 | 1.62 | 0.663 | 0.038 |
| 18WI_06.3 | 18.6 | 26.7 | 8.15 | 6.1 | 0.289 | 0.008 | 0.086 | 0.383 | 0.016 | 5 | 2.77 | 1.62 | 0.663 | 0.038 |
| 18WI_06.4 | 18.6 | 26.7 | 8.15 | 6.1 | 0.289 | 0.008 | 0.086 | 0.383 | 0.016 | 5 | 2.77 | 1.62 | 0.663 | 0.038 |
| 18WI_06.5 | 18.6 | 26.7 | 8.15 | 6.1 | 0.289 | 0.008 | 0.086 | 0.383 | 0.016 | 5 | 2.77 | 1.62 | 0.663 | 0.038 |
| 18WI_07.1 | 18.8 | 27.9 | 8.2 | 7.1 | 0.328 | 0.008 | 0.074 | 0.41 | 0.013 | 4.9 | 2.4 | 1.66 | 0.642 | 0.03 |
| 18WI_07.2 | 18.8 | 27.9 | 8.2 | 7.1 | 0.328 | 0.008 | 0.074 | 0.41 | 0.013 | 4.9 | 2.4 | 1.66 | 0.642 | 0.03 |
| 18WI_07.3 | 18.8 | 27.9 | 8.2 | 7.1 | 0.328 | 0.008 | 0.074 | 0.41 | 0.013 | 4.9 | 2.4 | 1.66 | 0.642 | 0.03 |
| 18WI_07.4 | 18.8 | 27.9 | 8.2 | 7.1 | 0.328 | 0.008 | 0.074 | 0.41 | 0.013 | 4.9 | 2.4 | 1.66 | 0.642 | 0.03 |
| 18WI_07.5 | 18.8 | 27.9 | 8.2 | 7.1 | 0.328 | 0.008 | 0.074 | 0.41 | 0.013 | 4.9 | 2.4 | 1.66 | 0.642 | 0.03 |
| 18WI_09.1 | 19.6 | 28.7 | 8.41 | 6.2 | 0.373 | 0.006 | 0.092 | 0.471 | 0.012 | 4.9 | 2.44 | 1.65 | 0.546 | 0.022 |
| 18WI_09.2 | 19.6 | 28.7 | 8.41 | 6.2 | 0.373 | 0.006 | 0.092 | 0.471 | 0.012 | 4.9 | 2.44 | 1.65 | 0.546 | 0.022 |
| 18WI_09.3 | 19.6 | 28.7 | 8.41 | 6.2 | 0.373 | 0.006 | 0.092 | 0.471 | 0.012 | 4.9 | 2.44 | 1.65 | 0.546 | 0.022 |
| 18WI_09.4 | 19.6 | 28.7 | 8.41 | 6.2 | 0.373 | 0.006 | 0.092 | 0.471 | 0.012 | 4.9 | 2.44 | 1.65 | 0.546 | 0.022 |
| 18WI_09.5 | 19.6 | 28.7 | 8.41 | 6.2 | 0.373 | 0.006 | 0.092 | 0.471 | 0.012 | 4.9 | 2.44 | 1.65 | 0.546 | 0.022 |
| 18WI_10.1 | 20.5 | 27.8 | 8.36 | 7.2 | 0.366 | 0.007 | 0.08 | 0.453 | 0.012 | 4.7 | 2.1 | 1.49 | 0.552 | 0.027 |
| 18WI_10.2 | 20.5 | 27.8 | 8.36 | 7.2 | 0.366 | 0.007 | 0.08 | 0.453 | 0.012 | 4.7 | 2.1 | 1.49 | 0.552 | 0.027 |
| 18WI_10.3 | 20.5 | 27.8 | 8.36 | 7.2 | 0.366 | 0.007 | 0.08 | 0.453 | 0.012 | 4.7 | 2.1 | 1.49 | 0.552 | 0.027 |
| 18WI_10.4 | 20.5 | 27.8 | 8.36 | 7.2 | 0.366 | 0.007 | 0.08 | 0.453 | 0.012 | 4.7 | 2.1 | 1.49 | 0.552 | 0.027 |
| 18WI_10.5 | 20.5 | 27.8 | 8.36 | 7.2 | 0.366 | 0.007 | 0.08 | 0.453 | 0.012 | 4.7 | 2.1 | 1.49 | 0.552 | 0.027 |
| 18WI_11.1 | 19.4 | 29.5 | 8.4 | 7.2 | 0.294 | 0.006 | 0.108 | 0.408 | 0.014 | 3.8 | 2.3 | 1.3 | 0.517 | 0.034 |
| 18WI_11.2 | 19.4 | 29.5 | 8.4 | 7.2 | 0.294 | 0.006 | 0.108 | 0.408 | 0.014 | 3.8 | 2.3 | 1.3 | 0.517 | 0.034 |
| 18WI_11.3 | 19.4 | 29.5 | 8.4 | 7.2 | 0.294 | 0.006 | 0.108 | 0.408 | 0.014 | 3.8 | 2.3 | 1.3 | 0.517 | 0.034 |
| 18WI_11.4 | 19.4 | 29.5 | 8.4 | 7.2 | 0.294 | 0.006 | 0.108 | 0.408 | 0.014 | 3.8 | 2.3 | 1.3 | 0.517 | 0.034 |
| 18WI_11.5 | 19.4 | 29.5 | 8.4 | 7.2 | 0.294 | 0.006 | 0.108 | 0.408 | 0.014 | 3.8 | 2.3 | 1.3 | 0.517 | 0.034 |
| 18WI_12.1 | 19.7 | 29.9 | 8.37 | 7.1 | 0.315 | 0.005 | 0.082 | 0.402 | 0.015 | 3.2 | 2.1 | 1.18 | 0.535 | 0.03 |
| 18WI_12.2 | 19.7 | 29.9 | 8.37 | 7.1 | 0.315 | 0.005 | 0.082 | 0.402 | 0.015 | 3.2 | 2.1 | 1.18 | 0.535 | 0.03 |
| 18WI_12.3 | 19.7 | 29.9 | 8.37 | 7.1 | 0.315 | 0.005 | 0.082 | 0.402 | 0.015 | 3.2 | 2.1 | 1.18 | 0.535 | 0.03 |
| 18WI_12.4 | 19.7 | 29.9 | 8.37 | 7.1 | 0.315 | 0.005 | 0.082 | 0.402 | 0.015 | 3.2 | 2.1 | 1.18 | 0.535 | 0.03 |
| 18WI_12.5 | 19.7 | 29.9 | 8.37 | 7.1 | 0.315 | 0.005 | 0.082 | 0.402 | 0.015 | 3.2 | 2.1 | 1.18 | 0.535 | 0.03 |
| 18WI_13.1 | 19 | 29.5 | 8.16 | 7.2 | 0.287 | 0.005 | 0.088 | 0.38 | 0.015 | 3.4 | 2.13 | 0.97 | 0.493 | 0.029 |
| 18WI_13.2 | 19 | 29.5 | 8.16 | 7.2 | 0.287 | 0.005 | 0.088 | 0.38 | 0.015 | 3.4 | 2.13 | 0.97 | 0.493 | 0.029 |
| 18WI_13.3 | 19 | 29.5 | 8.16 | 7.2 | 0.287 | 0.005 | 0.088 | 0.38 | 0.015 | 3.4 | 2.13 | 0.97 | 0.493 | 0.029 |
| 18WI_13.4 | 19 | 29.5 | 8.16 | 7.2 | 0.287 | 0.005 | 0.088 | 0.38 | 0.015 | 3.4 | 2.13 | 0.97 | 0.493 | 0.029 |
| 18WI_13.5 | 19 | 29.5 | 8.16 | 7.2 | 0.287 | 0.005 | 0.088 | 0.38 | 0.015 | 3.4 | 2.13 | 0.97 | 0.493 | 0.029 |
| 18WI_14.1 | 19.7 | 30.1 | 8.74 | 7.2 | 0.184 | 0.006 | 0.178 | 0.368 | 0.02 | 4.8 | 2.06 | 0.82 | 0.552 | 0.039 |
| 18WI_14.2 | 19.7 | 30.1 | 8.74 | 7.2 | 0.184 | 0.006 | 0.178 | 0.368 | 0.02 | 4.8 | 2.06 | 0.82 | 0.552 | 0.039 |
| 18WI_14.3 | 19.7 | 30.1 | 8.74 | 7.2 | 0.184 | 0.006 | 0.178 | 0.368 | 0.02 | 4.8 | 2.06 | 0.82 | 0.552 | 0.039 |
| 18WI_14.4 | 19.7 | 30.1 | 8.74 | 7.2 | 0.184 | 0.006 | 0.178 | 0.368 | 0.02 | 4.8 | 2.06 | 0.82 | 0.552 | 0.039 |
| 18WI_14.5 | 19.7 | 30.1 | 8.74 | 7.2 | 0.184 | 0.006 | 0.178 | 0.368 | 0.02 | 4.8 | 2.06 | 0.82 | 0.552 | 0.039 |
| 18WI_15.1 | 18.9 | 29.6 | 8.27 | 6.8 | 0.184 | 0.007 | 0.152 | 0.343 | 0.016 | 3.6 | 2.04 | 0.93 | 0.486 | 0.035 |
| 18WI_15.2 | 18.9 | 29.6 | 8.27 | 6.8 | 0.184 | 0.007 | 0.152 | 0.343 | 0.016 | 3.6 | 2.04 | 0.93 | 0.486 | 0.035 |
| 18WI_15.3 | 18.9 | 29.6 | 8.27 | 6.8 | 0.184 | 0.007 | 0.152 | 0.343 | 0.016 | 3.6 | 2.04 | 0.93 | 0.486 | 0.035 |
| 18WI_15.4 | 18.9 | 29.6 | 8.27 | 6.8 | 0.184 | 0.007 | 0.152 | 0.343 | 0.016 | 3.6 | 2.04 | 0.93 | 0.486 | 0.035 |
| 18WI_15.5 | 18.9 | 29.6 | 8.27 | 6.8 | 0.184 | 0.007 | 0.152 | 0.343 | 0.016 | 3.6 | 2.04 | 0.93 | 0.486 | 0.035 |
| 18WI_16.1 | 18.8 | 30.4 | 8.27 | 6.4 | 0.208 | 0.008 | 0.178 | 0.394 | 0.016 | 4 | 2.11 | 0.85 | 0.573 | 0.034 |
| 18WI_16.2 | 18.8 | 30.4 | 8.27 | 6.4 | 0.208 | 0.008 | 0.178 | 0.394 | 0.016 | 4 | 2.11 | 0.85 | 0.573 | 0.034 |
| 18WI_16.3 | 18.8 | 30.4 | 8.27 | 6.4 | 0.208 | 0.008 | 0.178 | 0.394 | 0.016 | 4 | 2.11 | 0.85 | 0.573 | 0.034 |
| 18WI_16.4 | 18.8 | 30.4 | 8.27 | 6.4 | 0.208 | 0.008 | 0.178 | 0.394 | 0.016 | 4 | 2.11 | 0.85 | 0.573 | 0.034 |
| 18WI_16.5 | 18.8 | 30.4 | 8.27 | 6.4 | 0.208 | 0.008 | 0.178 | 0.394 | 0.016 | 4 | 2.11 | 0.85 | 0.573 | 0.034 |
| 20AU_01.1 | 21.94 | 24.47 | 8.11 | 7.1 | 0.256 | 0.009 | 0.061 | 0.326 | 0.03 | 2.1 | 4.77 | 2.75 | 0.565 | 0.081 |
| 20AU_01.2 | 21.94 | 24.47 | 8.11 | 7.1 | 0.256 | 0.009 | 0.061 | 0.326 | 0.03 | 2.1 | 4.77 | 2.75 | 0.565 | 0.081 |
| 20AU_01.3 | 21.94 | 24.47 | 8.11 | 7.1 | 0.256 | 0.009 | 0.061 | 0.326 | 0.03 | 2.1 | 4.77 | 2.75 | 0.565 | 0.081 |
| 20AU_01.4 | 21.94 | 24.47 | 8.11 | 7.1 | 0.256 | 0.009 | 0.061 | 0.326 | 0.03 | 2.1 | 4.77 | 2.75 | 0.565 | 0.081 |
| 20AU_01.5 | 21.94 | 24.47 | 8.11 | 7.1 | 0.256 | 0.009 | 0.061 | 0.326 | 0.03 | 2.1 | 4.77 | 2.75 | 0.565 | 0.081 |
| 20AU_02.1 | 22.14 | 23.66 | 8.18 | 7.3 | 0.286 | 0.008 | 0.062 | 0.356 | 0.029 | 3.5 | 4.99 | 2.81 | 0.55 | 0.074 |
| 20AU_02.2 | 22.14 | 23.66 | 8.18 | 7.3 | 0.286 | 0.008 | 0.062 | 0.356 | 0.029 | 3.5 | 4.99 | 2.81 | 0.55 | 0.074 |
| 20AU_02.3 | 22.14 | 23.66 | 8.18 | 7.3 | 0.286 | 0.008 | 0.062 | 0.356 | 0.029 | 3.5 | 4.99 | 2.81 | 0.55 | 0.074 |
| 20AU_02.4 | 22.14 | 23.66 | 8.18 | 7.3 | 0.286 | 0.008 | 0.062 | 0.356 | 0.029 | 3.5 | 4.99 | 2.81 | 0.55 | 0.074 |
| 20AU_02.5 | 22.14 | 23.66 | 8.18 | 7.3 | 0.286 | 0.008 | 0.062 | 0.356 | 0.029 | 3.5 | 4.99 | 2.81 | 0.55 | 0.074 |
| 20AU_03.1 | 22.53 | 24.42 | 8.2 | 7.3 | 0.234 | 0.008 | 0.058 | 0.3 | 0.019 | 4.6 | 6.19 | 2.47 | 0.592 | 0.039 |
| 20AU_03.2 | 22.53 | 24.42 | 8.2 | 7.3 | 0.234 | 0.008 | 0.058 | 0.3 | 0.019 | 4.6 | 6.19 | 2.47 | 0.592 | 0.039 |
| 20AU_03.3 | 22.53 | 24.42 | 8.2 | 7.3 | 0.234 | 0.008 | 0.058 | 0.3 | 0.019 | 4.6 | 6.19 | 2.47 | 0.592 | 0.039 |
| 20AU_03.4 | 22.53 | 24.42 | 8.2 | 7.3 | 0.234 | 0.008 | 0.058 | 0.3 | 0.019 | 4.6 | 6.19 | 2.47 | 0.592 | 0.039 |
| 20AU_03.5 | 22.53 | 24.42 | 8.2 | 7.3 | 0.234 | 0.008 | 0.058 | 0.3 | 0.019 | 4.6 | 6.19 | 2.47 | 0.592 | 0.039 |
| 20AU_04.1 | 22.16 | 25.36 | 8.22 | 7.3 | 0.291 | 0.009 | 0.052 | 0.352 | 0.023 | 5.7 | 5.51 | 2.3 | 0.559 | 0.048 |
| 20AU_04.2 | 22.16 | 25.36 | 8.22 | 7.3 | 0.291 | 0.009 | 0.052 | 0.352 | 0.023 | 5.7 | 5.51 | 2.3 | 0.559 | 0.048 |
| 20AU_04.3 | 22.16 | 25.36 | 8.22 | 7.3 | 0.291 | 0.009 | 0.052 | 0.352 | 0.023 | 5.7 | 5.51 | 2.3 | 0.559 | 0.048 |
| 20AU_04.4 | 22.16 | 25.36 | 8.22 | 7.3 | 0.291 | 0.009 | 0.052 | 0.352 | 0.023 | 5.7 | 5.51 | 2.3 | 0.559 | 0.048 |
| 20AU_04.5 | 22.16 | 25.36 | 8.22 | 7.3 | 0.291 | 0.009 | 0.052 | 0.352 | 0.023 | 5.7 | 5.51 | 2.3 | 0.559 | 0.048 |
| 20AU_05.1 | 21.97 | 26.41 | 8.26 | 7.3 | 0.271 | 0.008 | 0.055 | 0.334 | 0.014 | 6.2 | 5.16 | 2.62 | 0.526 | 0.026 |
| 20AU_05.2 | 21.97 | 26.41 | 8.26 | 7.3 | 0.271 | 0.008 | 0.055 | 0.334 | 0.014 | 6.2 | 5.16 | 2.62 | 0.526 | 0.026 |
| 20AU_05.3 | 21.97 | 26.41 | 8.26 | 7.3 | 0.271 | 0.008 | 0.055 | 0.334 | 0.014 | 6.2 | 5.16 | 2.62 | 0.526 | 0.026 |
| 20AU_05.4 | 21.97 | 26.41 | 8.26 | 7.3 | 0.271 | 0.008 | 0.055 | 0.334 | 0.014 | 6.2 | 5.16 | 2.62 | 0.526 | 0.026 |
| 20AU_05.5 | 21.97 | 26.41 | 8.26 | 7.3 | 0.271 | 0.008 | 0.055 | 0.334 | 0.014 | 6.2 | 5.16 | 2.62 | 0.526 | 0.026 |
| 20AU_06.1 | 21.96 | 28.47 | 8.29 | 7.3 | 0.263 | 0.008 | 0.061 | 0.332 | 0.015 | 3.1 | 5.41 | 2.05 | 0.511 | 0.038 |
| 20AU_06.2 | 21.96 | 28.47 | 8.29 | 7.3 | 0.263 | 0.008 | 0.061 | 0.332 | 0.015 | 3.1 | 5.41 | 2.05 | 0.511 | 0.038 |
| 20AU_06.3 | 21.96 | 28.47 | 8.29 | 7.3 | 0.263 | 0.008 | 0.061 | 0.332 | 0.015 | 3.1 | 5.41 | 2.05 | 0.511 | 0.038 |
| 20AU_06.4 | 21.96 | 28.47 | 8.29 | 7.3 | 0.263 | 0.008 | 0.061 | 0.332 | 0.015 | 3.1 | 5.41 | 2.05 | 0.511 | 0.038 |
| 20AU_06.5 | 21.96 | 28.47 | 8.29 | 7.3 | 0.263 | 0.008 | 0.061 | 0.332 | 0.015 | 3.1 | 5.41 | 2.05 | 0.511 | 0.038 |
| 20AU_07.1 | 21.61 | 29.76 | 8.27 | 7.3 | 0.268 | 0.009 | 0.071 | 0.348 | 0.012 | 4.8 | 5.26 | 1.93 | 0.548 | 0.023 |
| 20AU_07.2 | 21.61 | 29.76 | 8.27 | 7.3 | 0.268 | 0.009 | 0.071 | 0.348 | 0.012 | 4.8 | 5.26 | 1.93 | 0.548 | 0.023 |
| 20AU_07.3 | 21.61 | 29.76 | 8.27 | 7.3 | 0.268 | 0.009 | 0.071 | 0.348 | 0.012 | 4.8 | 5.26 | 1.93 | 0.548 | 0.023 |
| 20AU_07.4 | 21.61 | 29.76 | 8.27 | 7.3 | 0.268 | 0.009 | 0.071 | 0.348 | 0.012 | 4.8 | 5.26 | 1.93 | 0.548 | 0.023 |
| 20AU_07.5 | 21.61 | 29.76 | 8.27 | 7.3 | 0.268 | 0.009 | 0.071 | 0.348 | 0.012 | 4.8 | 5.26 | 1.93 | 0.548 | 0.023 |
| 20AU_08.1 | 21.84 | 28.23 | 8.34 | 7.4 | 0.251 | 0.007 | 0.082 | 0.34 | 0.011 | 5.2 | 4.31 | 2.04 | 0.489 | 0.028 |
| 20AU_08.2 | 21.84 | 28.23 | 8.34 | 7.4 | 0.251 | 0.007 | 0.082 | 0.34 | 0.011 | 5.2 | 4.31 | 2.04 | 0.489 | 0.028 |
| 20AU_08.3 | 21.84 | 28.23 | 8.34 | 7.4 | 0.251 | 0.007 | 0.082 | 0.34 | 0.011 | 5.2 | 4.31 | 2.04 | 0.489 | 0.028 |
| 20AU_08.4 | 21.84 | 28.23 | 8.34 | 7.4 | 0.251 | 0.007 | 0.082 | 0.34 | 0.011 | 5.2 | 4.31 | 2.04 | 0.489 | 0.028 |
| 20AU_08.5 | 21.84 | 28.23 | 8.34 | 7.4 | 0.251 | 0.007 | 0.082 | 0.34 | 0.011 | 5.2 | 4.31 | 2.04 | 0.489 | 0.028 |
| 20AU_09.1 | 21.82 | 29.14 | 8.33 | 7.8 | 0.266 | 0.011 | 0.054 | 0.331 | 0.007 | 3.8 | 4.82 | 2 | 0.481 | 0.016 |
| 20AU_09.2 | 21.82 | 29.14 | 8.33 | 7.8 | 0.266 | 0.011 | 0.054 | 0.331 | 0.007 | 3.8 | 4.82 | 2 | 0.481 | 0.016 |
| 20AU_09.3 | 21.82 | 29.14 | 8.33 | 7.8 | 0.266 | 0.011 | 0.054 | 0.331 | 0.007 | 3.8 | 4.82 | 2 | 0.481 | 0.016 |
| 20AU_09.4 | 21.82 | 29.14 | 8.33 | 7.8 | 0.266 | 0.011 | 0.054 | 0.331 | 0.007 | 3.8 | 4.82 | 2 | 0.481 | 0.016 |
| 20AU_09.5 | 21.82 | 29.14 | 8.33 | 7.8 | 0.266 | 0.011 | 0.054 | 0.331 | 0.007 | 3.8 | 4.82 | 2 | 0.481 | 0.016 |
| 20AU_10.1 | 21.95 | 28.72 | 8.3 | 7.4 | 0.251 | 0.013 | 0.031 | 0.295 | 0.007 | 4.6 | 4.69 | 1.8 | 0.375 | 0.015 |
| 20AU_10.2 | 21.95 | 28.72 | 8.3 | 7.4 | 0.251 | 0.013 | 0.031 | 0.295 | 0.007 | 4.6 | 4.69 | 1.8 | 0.375 | 0.015 |
| 20AU_10.3 | 21.95 | 28.72 | 8.3 | 7.4 | 0.251 | 0.013 | 0.031 | 0.295 | 0.007 | 4.6 | 4.69 | 1.8 | 0.375 | 0.015 |
| 20AU_10.4 | 21.95 | 28.72 | 8.3 | 7.4 | 0.251 | 0.013 | 0.031 | 0.295 | 0.007 | 4.6 | 4.69 | 1.8 | 0.375 | 0.015 |
| 20AU_10.5 | 21.95 | 28.72 | 8.3 | 7.4 | 0.251 | 0.013 | 0.031 | 0.295 | 0.007 | 4.6 | 4.69 | 1.8 | 0.375 | 0.015 |
| 20AU_11.1 | 22.18 | 29.15 | 8.38 | 8.1 | 0.241 | 0.011 | 0.021 | 0.273 | 0.005 | 4.9 | 4.14 | 1.41 | 0.361 | 0.012 |
| 20AU_11.2 | 22.18 | 29.15 | 8.38 | 8.1 | 0.241 | 0.011 | 0.021 | 0.273 | 0.005 | 4.9 | 4.14 | 1.41 | 0.361 | 0.012 |
| 20AU_11.3 | 22.18 | 29.15 | 8.38 | 8.1 | 0.241 | 0.011 | 0.021 | 0.273 | 0.005 | 4.9 | 4.14 | 1.41 | 0.361 | 0.012 |
| 20AU_11.4 | 22.18 | 29.15 | 8.38 | 8.1 | 0.241 | 0.011 | 0.021 | 0.273 | 0.005 | 4.9 | 4.14 | 1.41 | 0.361 | 0.012 |
| 20AU_11.5 | 22.18 | 29.15 | 8.38 | 8.1 | 0.241 | 0.011 | 0.021 | 0.273 | 0.005 | 4.9 | 4.14 | 1.41 | 0.361 | 0.012 |
| 20AU_12.1 | 22.29 | 30.11 | 8.33 | 7.7 | 0.198 | 0.012 | 0.022 | 0.232 | 0.006 | 5.7 | 8 | 1.47 | 0.372 | 0.016 |
| 20AU_12.2 | 22.29 | 30.11 | 8.33 | 7.7 | 0.198 | 0.012 | 0.022 | 0.232 | 0.006 | 5.7 | 8 | 1.47 | 0.372 | 0.016 |
| 20AU_12.3 | 22.29 | 30.11 | 8.33 | 7.7 | 0.198 | 0.012 | 0.022 | 0.232 | 0.006 | 5.7 | 8 | 1.47 | 0.372 | 0.016 |
| 20AU_12.4 | 22.29 | 30.11 | 8.33 | 7.7 | 0.198 | 0.012 | 0.022 | 0.232 | 0.006 | 5.7 | 8 | 1.47 | 0.372 | 0.016 |
| 20AU_12.5 | 22.29 | 30.11 | 8.33 | 7.7 | 0.198 | 0.012 | 0.022 | 0.232 | 0.006 | 5.7 | 8 | 1.47 | 0.372 | 0.016 |
| 20AU_13.1 | 22.16 | 31.26 | 8.36 | 7.6 | 0.152 | 0.015 | 0.028 | 0.195 | 0.007 | 6.4 | 7.54 | 1.49 | 0.349 | 0.013 |
| 20AU_13.2 | 22.16 | 31.26 | 8.36 | 7.6 | 0.152 | 0.015 | 0.028 | 0.195 | 0.007 | 6.4 | 7.54 | 1.49 | 0.349 | 0.013 |
| 20AU_13.3 | 22.16 | 31.26 | 8.36 | 7.6 | 0.152 | 0.015 | 0.028 | 0.195 | 0.007 | 6.4 | 7.54 | 1.49 | 0.349 | 0.013 |
| 20AU_13.4 | 22.16 | 31.26 | 8.36 | 7.6 | 0.152 | 0.015 | 0.028 | 0.195 | 0.007 | 6.4 | 7.54 | 1.49 | 0.349 | 0.013 |
| 20AU_13.5 | 22.16 | 31.26 | 8.36 | 7.6 | 0.152 | 0.015 | 0.028 | 0.195 | 0.007 | 6.4 | 7.54 | 1.49 | 0.349 | 0.013 |
| 20AU_14.1 | 22.11 | 30.18 | 8.35 | 7.9 | 0.116 | 0.011 | 0.024 | 0.151 | 0.006 | 5.8 | 4.63 | 1.57 | 0.333 | 0.011 |
| 20AU_14.2 | 22.11 | 30.18 | 8.35 | 7.9 | 0.116 | 0.011 | 0.024 | 0.151 | 0.006 | 5.8 | 4.63 | 1.57 | 0.333 | 0.011 |
| 20AU_14.3 | 22.11 | 30.18 | 8.35 | 7.9 | 0.116 | 0.011 | 0.024 | 0.151 | 0.006 | 5.8 | 4.63 | 1.57 | 0.333 | 0.011 |
| 20AU_14.4 | 22.11 | 30.18 | 8.35 | 7.9 | 0.116 | 0.011 | 0.024 | 0.151 | 0.006 | 5.8 | 4.63 | 1.57 | 0.333 | 0.011 |
| 20AU_14.5 | 22.11 | 30.18 | 8.35 | 7.9 | 0.116 | 0.011 | 0.024 | 0.151 | 0.006 | 5.8 | 4.63 | 1.57 | 0.333 | 0.011 |
| 20AU_15.1 | 22.48 | 30.76 | 8.38 | 8 | 0.105 | 0.008 | 0.02 | 0.133 | 0.006 | 5.9 | 6.03 | 1.46 | 0.473 | 0.011 |
| 20AU_15.2 | 22.48 | 30.76 | 8.38 | 8 | 0.105 | 0.008 | 0.02 | 0.133 | 0.006 | 5.9 | 6.03 | 1.46 | 0.473 | 0.011 |
| 20AU_15.3 | 22.48 | 30.76 | 8.38 | 8 | 0.105 | 0.008 | 0.02 | 0.133 | 0.006 | 5.9 | 6.03 | 1.46 | 0.473 | 0.011 |
| 20AU_15.4 | 22.48 | 30.76 | 8.38 | 8 | 0.105 | 0.008 | 0.02 | 0.133 | 0.006 | 5.9 | 6.03 | 1.46 | 0.473 | 0.011 |
| 20AU_15.5 | 22.48 | 30.76 | 8.38 | 8 | 0.105 | 0.008 | 0.02 | 0.133 | 0.006 | 5.9 | 6.03 | 1.46 | 0.473 | 0.011 |
| 20AU_16.1 | 21.93 | 30.61 | 8.4 | 8.2 | 0.112 | 0.009 | 0.028 | 0.149 | 0.006 | 3.1 | 4.96 | 2.03 | 0.341 | 0.012 |
| 20AU_16.2 | 21.93 | 30.61 | 8.4 | 8.2 | 0.112 | 0.009 | 0.028 | 0.149 | 0.006 | 3.1 | 4.96 | 2.03 | 0.341 | 0.012 |
| 20AU_16.3 | 21.93 | 30.61 | 8.4 | 8.2 | 0.112 | 0.009 | 0.028 | 0.149 | 0.006 | 3.1 | 4.96 | 2.03 | 0.341 | 0.012 |
| 20AU_16.4 | 21.93 | 30.61 | 8.4 | 8.2 | 0.112 | 0.009 | 0.028 | 0.149 | 0.006 | 3.1 | 4.96 | 2.03 | 0.341 | 0.012 |
| 20AU_16.5 | 21.93 | 30.61 | 8.4 | 8.2 | 0.112 | 0.009 | 0.028 | 0.149 | 0.006 | 3.1 | 4.96 | 2.03 | 0.341 | 0.012 |
| 20SP_01.1 | 21.7 | 21.82 | 7.96 | 7.1 | 0.94 | 0.044 | 0.02 | 1.004 | 0.022 | 1.3 | 3.74 | 2.73 | 1.756 | 0.05 |
| 20SP_01.2 | 21.7 | 21.82 | 7.96 | 7.1 | 0.94 | 0.044 | 0.02 | 1.004 | 0.022 | 1.3 | 3.74 | 2.73 | 1.756 | 0.05 |
| 20SP_01.3 | 21.7 | 21.82 | 7.96 | 7.1 | 0.94 | 0.044 | 0.02 | 1.004 | 0.022 | 1.3 | 3.74 | 2.73 | 1.756 | 0.05 |
| 20SP_01.4 | 21.7 | 21.82 | 7.96 | 7.1 | 0.94 | 0.044 | 0.02 | 1.004 | 0.022 | 1.3 | 3.74 | 2.73 | 1.756 | 0.05 |
| 20SP_01.5 | 21.7 | 21.82 | 7.96 | 7.1 | 0.94 | 0.044 | 0.02 | 1.004 | 0.022 | 1.3 | 3.74 | 2.73 | 1.756 | 0.05 |
| 20SP_02.1 | 21.8 | 22.28 | 7.98 | 7.2 | 0.76 | 0.036 | 0.102 | 0.898 | 0.018 | 1.5 | 3.17 | 2.41 | 1.361 | 0.035 |
| 20SP_02.2 | 21.8 | 22.28 | 7.98 | 7.2 | 0.76 | 0.036 | 0.102 | 0.898 | 0.018 | 1.5 | 3.17 | 2.41 | 1.361 | 0.035 |
| 20SP_02.3 | 21.8 | 22.28 | 7.98 | 7.2 | 0.76 | 0.036 | 0.102 | 0.898 | 0.018 | 1.5 | 3.17 | 2.41 | 1.361 | 0.035 |
| 20SP_02.4 | 21.8 | 22.28 | 7.98 | 7.2 | 0.76 | 0.036 | 0.102 | 0.898 | 0.018 | 1.5 | 3.17 | 2.41 | 1.361 | 0.035 |
| 20SP_02.5 | 21.8 | 22.28 | 7.98 | 7.2 | 0.76 | 0.036 | 0.102 | 0.898 | 0.018 | 1.5 | 3.17 | 2.41 | 1.361 | 0.035 |
| 20SP_03.1 | 21.8 | 23.43 | 8.01 | 7.4 | 0.57 | 0.023 | 0.016 | 0.609 | 0.016 | 0.8 | 2.91 | 2.86 | 0.955 | 0.032 |
| 20SP_03.2 | 21.8 | 23.43 | 8.01 | 7.4 | 0.57 | 0.023 | 0.016 | 0.609 | 0.016 | 0.8 | 2.91 | 2.86 | 0.955 | 0.032 |
| 20SP_03.3 | 21.8 | 23.43 | 8.01 | 7.4 | 0.57 | 0.023 | 0.016 | 0.609 | 0.016 | 0.8 | 2.91 | 2.86 | 0.955 | 0.032 |
| 20SP_03.4 | 21.8 | 23.43 | 8.01 | 7.4 | 0.57 | 0.023 | 0.016 | 0.609 | 0.016 | 0.8 | 2.91 | 2.86 | 0.955 | 0.032 |
| 20SP_03.5 | 21.8 | 23.43 | 8.01 | 7.4 | 0.57 | 0.023 | 0.016 | 0.609 | 0.016 | 0.8 | 2.91 | 2.86 | 0.955 | 0.032 |
| 20SP_04.1 | 21.6 | 24.94 | 8.08 | 7.5 | 0.23 | 0.018 | 0.023 | 0.271 | 0.014 | 2.1 | 2.75 | 2.3 | 0.481 | 0.031 |
| 20SP_04.2 | 21.6 | 24.94 | 8.08 | 7.5 | 0.23 | 0.018 | 0.023 | 0.271 | 0.014 | 2.1 | 2.75 | 2.3 | 0.481 | 0.031 |
| 20SP_04.3 | 21.6 | 24.94 | 8.08 | 7.5 | 0.23 | 0.018 | 0.023 | 0.271 | 0.014 | 2.1 | 2.75 | 2.3 | 0.481 | 0.031 |
| 20SP_04.4 | 21.6 | 24.94 | 8.08 | 7.5 | 0.23 | 0.018 | 0.023 | 0.271 | 0.014 | 2.1 | 2.75 | 2.3 | 0.481 | 0.031 |
| 20SP_04.5 | 21.6 | 24.94 | 8.08 | 7.5 | 0.23 | 0.018 | 0.023 | 0.271 | 0.014 | 2.1 | 2.75 | 2.3 | 0.481 | 0.031 |
| 20SP_05.1 | 21.5 | 25.81 | 8.04 | 7.6 | 0.29 | 0.032 | 0.026 | 0.348 | 0.013 | 1.7 | 2.79 | 2.45 | 0.602 | 0.027 |
| 20SP_05.2 | 21.5 | 25.81 | 8.04 | 7.6 | 0.29 | 0.032 | 0.026 | 0.348 | 0.013 | 1.7 | 2.79 | 2.45 | 0.602 | 0.027 |
| 20SP_05.3 | 21.5 | 25.81 | 8.04 | 7.6 | 0.29 | 0.032 | 0.026 | 0.348 | 0.013 | 1.7 | 2.79 | 2.45 | 0.602 | 0.027 |
| 20SP_05.4 | 21.5 | 25.81 | 8.04 | 7.6 | 0.29 | 0.032 | 0.026 | 0.348 | 0.013 | 1.7 | 2.79 | 2.45 | 0.602 | 0.027 |
| 20SP_05.5 | 21.5 | 25.81 | 8.04 | 7.6 | 0.29 | 0.032 | 0.026 | 0.348 | 0.013 | 1.7 | 2.79 | 2.45 | 0.602 | 0.027 |
| 20SP_06.1 | 21 | 28.66 | 8.11 | 7.5 | 0.35 | 0.037 | 0.041 | 0.428 | 0.011 | 1.7 | 2.39 | 2.12 | 0.79 | 0.026 |
| 20SP_06.2 | 21 | 28.66 | 8.11 | 7.5 | 0.35 | 0.037 | 0.041 | 0.428 | 0.011 | 1.7 | 2.39 | 2.12 | 0.79 | 0.026 |
| 20SP_06.3 | 21 | 28.66 | 8.11 | 7.5 | 0.35 | 0.037 | 0.041 | 0.428 | 0.011 | 1.7 | 2.39 | 2.12 | 0.79 | 0.026 |
| 20SP_06.4 | 21 | 28.66 | 8.11 | 7.5 | 0.35 | 0.037 | 0.041 | 0.428 | 0.011 | 1.7 | 2.39 | 2.12 | 0.79 | 0.026 |
| 20SP_06.5 | 21 | 28.66 | 8.11 | 7.5 | 0.35 | 0.037 | 0.041 | 0.428 | 0.011 | 1.7 | 2.39 | 2.12 | 0.79 | 0.026 |
| 20SP_07.1 | 20.8 | 29.34 | 8.06 | 7.5 | 0.33 | 0.035 | 0.055 | 0.42 | 0.01 | 1.7 | 2.28 | 2.1 | 0.65 | 0.023 |
| 20SP_07.2 | 20.8 | 29.34 | 8.06 | 7.5 | 0.33 | 0.035 | 0.055 | 0.42 | 0.01 | 1.7 | 2.28 | 2.1 | 0.65 | 0.023 |
| 20SP_07.3 | 20.8 | 29.34 | 8.06 | 7.5 | 0.33 | 0.035 | 0.055 | 0.42 | 0.01 | 1.7 | 2.28 | 2.1 | 0.65 | 0.023 |
| 20SP_07.4 | 20.8 | 29.34 | 8.06 | 7.5 | 0.33 | 0.035 | 0.055 | 0.42 | 0.01 | 1.7 | 2.28 | 2.1 | 0.65 | 0.023 |
| 20SP_07.5 | 20.8 | 29.34 | 8.06 | 7.5 | 0.33 | 0.035 | 0.055 | 0.42 | 0.01 | 1.7 | 2.28 | 2.1 | 0.65 | 0.023 |
| 20SP_08.1 | 20.4 | 27.92 | 8.24 | 7.5 | 0.35 | 0.023 | 0.032 | 0.405 | 0.011 | 0.9 | 2.43 | 2.06 | 0.521 | 0.021 |
| 20SP_08.2 | 20.4 | 27.92 | 8.24 | 7.5 | 0.35 | 0.023 | 0.032 | 0.405 | 0.011 | 0.9 | 2.43 | 2.06 | 0.521 | 0.021 |
| 20SP_08.3 | 20.4 | 27.92 | 8.24 | 7.5 | 0.35 | 0.023 | 0.032 | 0.405 | 0.011 | 0.9 | 2.43 | 2.06 | 0.521 | 0.021 |
| 20SP_08.4 | 20.4 | 27.92 | 8.24 | 7.5 | 0.35 | 0.023 | 0.032 | 0.405 | 0.011 | 0.9 | 2.43 | 2.06 | 0.521 | 0.021 |
| 20SP_08.5 | 20.4 | 27.92 | 8.24 | 7.5 | 0.35 | 0.023 | 0.032 | 0.405 | 0.011 | 0.9 | 2.43 | 2.06 | 0.521 | 0.021 |
| 20SP_09.1 | 21.1 | 30.73 | 8.18 | 7.5 | 0.52 | 0.019 | 0.053 | 0.592 | 0.007 | 1.1 | 2.26 | 2.13 | 0.716 | 0.014 |
| 20SP_09.2 | 21.1 | 30.73 | 8.18 | 7.5 | 0.52 | 0.019 | 0.053 | 0.592 | 0.007 | 1.1 | 2.26 | 2.13 | 0.716 | 0.014 |
| 20SP_09.3 | 21.1 | 30.73 | 8.18 | 7.5 | 0.52 | 0.019 | 0.053 | 0.592 | 0.007 | 1.1 | 2.26 | 2.13 | 0.716 | 0.014 |
| 20SP_09.4 | 21.1 | 30.73 | 8.18 | 7.5 | 0.52 | 0.019 | 0.053 | 0.592 | 0.007 | 1.1 | 2.26 | 2.13 | 0.716 | 0.014 |
| 20SP_09.5 | 21.1 | 30.73 | 8.18 | 7.5 | 0.52 | 0.019 | 0.053 | 0.592 | 0.007 | 1.1 | 2.26 | 2.13 | 0.716 | 0.014 |
| 20SP_10.1 | 20.6 | 28.29 | 8.06 | 7.5 | 0.34 | 0.022 | 0.04 | 0.402 | 0.008 | 1.1 | 2.29 | 2.13 | 0.51 | 0.016 |
| 20SP_10.2 | 20.6 | 28.29 | 8.06 | 7.5 | 0.34 | 0.022 | 0.04 | 0.402 | 0.008 | 1.1 | 2.29 | 2.13 | 0.51 | 0.016 |
| 20SP_10.3 | 20.6 | 28.29 | 8.06 | 7.5 | 0.34 | 0.022 | 0.04 | 0.402 | 0.008 | 1.1 | 2.29 | 2.13 | 0.51 | 0.016 |
| 20SP_10.4 | 20.6 | 28.29 | 8.06 | 7.5 | 0.34 | 0.022 | 0.04 | 0.402 | 0.008 | 1.1 | 2.29 | 2.13 | 0.51 | 0.016 |
| 20SP_10.5 | 20.6 | 28.29 | 8.06 | 7.5 | 0.34 | 0.022 | 0.04 | 0.402 | 0.008 | 1.1 | 2.29 | 2.13 | 0.51 | 0.016 |
| 20SP_11.1 | 20.6 | 30.86 | 8.06 | 7.6 | 0.33 | 0.042 | 0.064 | 0.436 | 0.007 | 2.1 | 1.97 | 1.98 | 0.526 | 0.018 |
| 20SP_11.2 | 20.6 | 30.86 | 8.06 | 7.6 | 0.33 | 0.042 | 0.064 | 0.436 | 0.007 | 2.1 | 1.97 | 1.98 | 0.526 | 0.018 |
| 20SP_11.3 | 20.6 | 30.86 | 8.06 | 7.6 | 0.33 | 0.042 | 0.064 | 0.436 | 0.007 | 2.1 | 1.97 | 1.98 | 0.526 | 0.018 |
| 20SP_11.4 | 20.6 | 30.86 | 8.06 | 7.6 | 0.33 | 0.042 | 0.064 | 0.436 | 0.007 | 2.1 | 1.97 | 1.98 | 0.526 | 0.018 |
| 20SP_11.5 | 20.6 | 30.86 | 8.06 | 7.6 | 0.33 | 0.042 | 0.064 | 0.436 | 0.007 | 2.1 | 1.97 | 1.98 | 0.526 | 0.018 |
| 20SP_12.1 | 21.6 | 31.84 | 8.13 | 7.3 | 0.63 | 0.023 | 0.048 | 0.701 | 0.007 | 1.3 | 1.87 | 1.31 | 0.99 | 0.019 |
| 20SP_12.2 | 21.6 | 31.84 | 8.13 | 7.3 | 0.63 | 0.023 | 0.048 | 0.701 | 0.007 | 1.3 | 1.87 | 1.31 | 0.99 | 0.019 |
| 20SP_12.3 | 21.6 | 31.84 | 8.13 | 7.3 | 0.63 | 0.023 | 0.048 | 0.701 | 0.007 | 1.3 | 1.87 | 1.31 | 0.99 | 0.019 |
| 20SP_12.4 | 21.6 | 31.84 | 8.13 | 7.3 | 0.63 | 0.023 | 0.048 | 0.701 | 0.007 | 1.3 | 1.87 | 1.31 | 0.99 | 0.019 |
| 20SP_12.5 | 21.6 | 31.84 | 8.13 | 7.3 | 0.63 | 0.023 | 0.048 | 0.701 | 0.007 | 1.3 | 1.87 | 1.31 | 0.99 | 0.019 |
| 20SP_13.1 | 20.6 | 32.61 | 8.02 | 7.5 | 0.35 | 0.021 | 0.041 | 0.412 | 0.007 | 2.8 | 1.91 | 1.26 | 0.586 | 0.015 |
| 20SP_13.2 | 20.6 | 32.61 | 8.02 | 7.5 | 0.35 | 0.021 | 0.041 | 0.412 | 0.007 | 2.8 | 1.91 | 1.26 | 0.586 | 0.015 |
| 20SP_13.3 | 20.6 | 32.61 | 8.02 | 7.5 | 0.35 | 0.021 | 0.041 | 0.412 | 0.007 | 2.8 | 1.91 | 1.26 | 0.586 | 0.015 |
| 20SP_13.4 | 20.6 | 32.61 | 8.02 | 7.5 | 0.35 | 0.021 | 0.041 | 0.412 | 0.007 | 2.8 | 1.91 | 1.26 | 0.586 | 0.015 |
| 20SP_13.5 | 20.6 | 32.61 | 8.02 | 7.5 | 0.35 | 0.021 | 0.041 | 0.412 | 0.007 | 2.8 | 1.91 | 1.26 | 0.586 | 0.015 |
| 20SP_14.1 | 21.4 | 31.88 | 7.99 | 7.3 | 0.377 | 0.019 | 0.053 | 0.449 | 0.006 | 3.3 | 2.02 | 1.34 | 0.644 | 0.014 |
| 20SP_14.2 | 21.4 | 31.88 | 7.99 | 7.3 | 0.377 | 0.019 | 0.053 | 0.449 | 0.006 | 3.3 | 2.02 | 1.34 | 0.644 | 0.014 |
| 20SP_14.3 | 21.4 | 31.88 | 7.99 | 7.3 | 0.377 | 0.019 | 0.053 | 0.449 | 0.006 | 3.3 | 2.02 | 1.34 | 0.644 | 0.014 |
| 20SP_14.4 | 21.4 | 31.88 | 7.99 | 7.3 | 0.377 | 0.019 | 0.053 | 0.449 | 0.006 | 3.3 | 2.02 | 1.34 | 0.644 | 0.014 |
| 20SP_14.5 | 21.4 | 31.88 | 7.99 | 7.3 | 0.377 | 0.019 | 0.053 | 0.449 | 0.006 | 3.3 | 2.02 | 1.34 | 0.644 | 0.014 |
| 20SP_15.1 | 21.6 | 32.28 | 8.12 | 7.4 | 0.33 | 0.02 | 0.055 | 0.405 | 0.005 | 3 | 1.91 | 1.27 | 0.63 | 0.013 |
| 20SP_15.2 | 21.6 | 32.28 | 8.12 | 7.4 | 0.33 | 0.02 | 0.055 | 0.405 | 0.005 | 3 | 1.91 | 1.27 | 0.63 | 0.013 |
| 20SP_15.3 | 21.6 | 32.28 | 8.12 | 7.4 | 0.33 | 0.02 | 0.055 | 0.405 | 0.005 | 3 | 1.91 | 1.27 | 0.63 | 0.013 |
| 20SP_15.4 | 21.6 | 32.28 | 8.12 | 7.4 | 0.33 | 0.02 | 0.055 | 0.405 | 0.005 | 3 | 1.91 | 1.27 | 0.63 | 0.013 |
| 20SP_15.5 | 21.6 | 32.28 | 8.12 | 7.4 | 0.33 | 0.02 | 0.055 | 0.405 | 0.005 | 3 | 1.91 | 1.27 | 0.63 | 0.013 |
| 20SP_16.1 | 21.8 | 32.36 | 8.11 | 7.5 | 0.312 | 0.014 | 0.049 | 0.375 | 0.006 | 3.4 | 1.73 | 1.3 | 0.57 | 0.012 |
| 20SP_16.2 | 21.8 | 32.36 | 8.11 | 7.5 | 0.312 | 0.014 | 0.049 | 0.375 | 0.006 | 3.4 | 1.73 | 1.3 | 0.57 | 0.012 |
| 20SP_16.3 | 21.8 | 32.36 | 8.11 | 7.5 | 0.312 | 0.014 | 0.049 | 0.375 | 0.006 | 3.4 | 1.73 | 1.3 | 0.57 | 0.012 |
| 20SP_16.4 | 21.8 | 32.36 | 8.11 | 7.5 | 0.312 | 0.014 | 0.049 | 0.375 | 0.006 | 3.4 | 1.73 | 1.3 | 0.57 | 0.012 |
| 20SP_16.5 | 21.8 | 32.36 | 8.11 | 7.5 | 0.312 | 0.014 | 0.049 | 0.375 | 0.006 | 3.4 | 1.73 | 1.3 | 0.57 | 0.012 |
| 20SU_01.1 | 32.1 | 18.81 | 7.78 | 5.4 | 0.683 | 0.039 | 0.119 | 0.841 | 0.027 | 1.4 | 4.32 | 2.81 | 1.127 | 0.054 |
| 20SU_01.2 | 32.1 | 18.81 | 7.78 | 5.4 | 0.683 | 0.039 | 0.119 | 0.841 | 0.027 | 1.4 | 4.32 | 2.81 | 1.127 | 0.054 |
| 20SU_01.3 | 32.1 | 18.81 | 7.78 | 5.4 | 0.683 | 0.039 | 0.119 | 0.841 | 0.027 | 1.4 | 4.32 | 2.81 | 1.127 | 0.054 |
| 20SU_01.4 | 32.1 | 18.81 | 7.78 | 5.4 | 0.683 | 0.039 | 0.119 | 0.841 | 0.027 | 1.4 | 4.32 | 2.81 | 1.127 | 0.054 |
| 20SU_01.5 | 32.1 | 18.81 | 7.78 | 5.4 | 0.683 | 0.039 | 0.119 | 0.841 | 0.027 | 1.4 | 4.32 | 2.81 | 1.127 | 0.054 |
| 20SU_02.1 | 32 | 18.92 | 7.75 | 5.1 | 0.361 | 0.025 | 0.078 | 0.464 | 0.022 | 2.7 | 4.41 | 2.78 | 0.557 | 0.041 |
| 20SU_02.2 | 32 | 18.92 | 7.75 | 5.1 | 0.361 | 0.025 | 0.078 | 0.464 | 0.022 | 2.7 | 4.41 | 2.78 | 0.557 | 0.041 |
| 20SU_02.3 | 32 | 18.92 | 7.75 | 5.1 | 0.361 | 0.025 | 0.078 | 0.464 | 0.022 | 2.7 | 4.41 | 2.78 | 0.557 | 0.041 |
| 20SU_02.4 | 32 | 18.92 | 7.75 | 5.1 | 0.361 | 0.025 | 0.078 | 0.464 | 0.022 | 2.7 | 4.41 | 2.78 | 0.557 | 0.041 |
| 20SU_02.5 | 32 | 18.92 | 7.75 | 5.1 | 0.361 | 0.025 | 0.078 | 0.464 | 0.022 | 2.7 | 4.41 | 2.78 | 0.557 | 0.041 |
| 20SU_03.1 | 32.2 | 19.12 | 7.8 | 5.6 | 0.381 | 0.025 | 0.076 | 0.482 | 0.018 | 4.1 | 4.18 | 2.32 | 0.531 | 0.032 |
| 20SU_03.2 | 32.2 | 19.12 | 7.8 | 5.6 | 0.381 | 0.025 | 0.076 | 0.482 | 0.018 | 4.1 | 4.18 | 2.32 | 0.531 | 0.032 |
| 20SU_03.3 | 32.2 | 19.12 | 7.8 | 5.6 | 0.381 | 0.025 | 0.076 | 0.482 | 0.018 | 4.1 | 4.18 | 2.32 | 0.531 | 0.032 |
| 20SU_03.4 | 32.2 | 19.12 | 7.8 | 5.6 | 0.381 | 0.025 | 0.076 | 0.482 | 0.018 | 4.1 | 4.18 | 2.32 | 0.531 | 0.032 |
| 20SU_03.5 | 32.2 | 19.12 | 7.8 | 5.6 | 0.381 | 0.025 | 0.076 | 0.482 | 0.018 | 4.1 | 4.18 | 2.32 | 0.531 | 0.032 |
| 20SU_04.1 | 32.2 | 20.82 | 7.86 | 5.7 | 0.382 | 0.026 | 0.071 | 0.479 | 0.017 | 4.1 | 3.13 | 2.52 | 0.585 | 0.04 |
| 20SU_04.2 | 32.2 | 20.82 | 7.86 | 5.7 | 0.382 | 0.026 | 0.071 | 0.479 | 0.017 | 4.1 | 3.13 | 2.52 | 0.585 | 0.04 |
| 20SU_04.3 | 32.2 | 20.82 | 7.86 | 5.7 | 0.382 | 0.026 | 0.071 | 0.479 | 0.017 | 4.1 | 3.13 | 2.52 | 0.585 | 0.04 |
| 20SU_04.4 | 32.2 | 20.82 | 7.86 | 5.7 | 0.382 | 0.026 | 0.071 | 0.479 | 0.017 | 4.1 | 3.13 | 2.52 | 0.585 | 0.04 |
| 20SU_04.5 | 32.2 | 20.82 | 7.86 | 5.7 | 0.382 | 0.026 | 0.071 | 0.479 | 0.017 | 4.1 | 3.13 | 2.52 | 0.585 | 0.04 |
| 20SU_05.1 | 31.8 | 21.74 | 7.84 | 5.6 | 0.375 | 0.025 | 0.071 | 0.471 | 0.016 | 2.7 | 3.11 | 2.33 | 0.584 | 0.039 |
| 20SU_05.2 | 31.8 | 21.74 | 7.84 | 5.6 | 0.375 | 0.025 | 0.071 | 0.471 | 0.016 | 2.7 | 3.11 | 2.33 | 0.584 | 0.039 |
| 20SU_05.3 | 31.8 | 21.74 | 7.84 | 5.6 | 0.375 | 0.025 | 0.071 | 0.471 | 0.016 | 2.7 | 3.11 | 2.33 | 0.584 | 0.039 |
| 20SU_05.4 | 31.8 | 21.74 | 7.84 | 5.6 | 0.375 | 0.025 | 0.071 | 0.471 | 0.016 | 2.7 | 3.11 | 2.33 | 0.584 | 0.039 |
| 20SU_05.5 | 31.8 | 21.74 | 7.84 | 5.6 | 0.375 | 0.025 | 0.071 | 0.471 | 0.016 | 2.7 | 3.11 | 2.33 | 0.584 | 0.039 |
| 20SU_06.1 | 31.4 | 23.17 | 7.81 | 5 | 0.406 | 0.038 | 0.072 | 0.516 | 0.012 | 4.8 | 3.11 | 2.25 | 0.69 | 0.027 |
| 20SU_06.2 | 31.4 | 23.17 | 7.81 | 5 | 0.406 | 0.038 | 0.072 | 0.516 | 0.012 | 4.8 | 3.11 | 2.25 | 0.69 | 0.027 |
| 20SU_06.3 | 31.4 | 23.17 | 7.81 | 5 | 0.406 | 0.038 | 0.072 | 0.516 | 0.012 | 4.8 | 3.11 | 2.25 | 0.69 | 0.027 |
| 20SU_06.4 | 31.4 | 23.17 | 7.81 | 5 | 0.406 | 0.038 | 0.072 | 0.516 | 0.012 | 4.8 | 3.11 | 2.25 | 0.69 | 0.027 |
| 20SU_06.5 | 31.4 | 23.17 | 7.81 | 5 | 0.406 | 0.038 | 0.072 | 0.516 | 0.012 | 4.8 | 3.11 | 2.25 | 0.69 | 0.027 |
| 20SU_07.1 | 31.4 | 23.54 | 7.86 | 4.9 | 0.401 | 0.028 | 0.077 | 0.506 | 0.009 | 3 | 3.13 | 2.41 | 0.698 | 0.018 |
| 20SU_07.2 | 31.4 | 23.54 | 7.86 | 4.9 | 0.401 | 0.028 | 0.077 | 0.506 | 0.009 | 3 | 3.13 | 2.41 | 0.698 | 0.018 |
| 20SU_07.3 | 31.4 | 23.54 | 7.86 | 4.9 | 0.401 | 0.028 | 0.077 | 0.506 | 0.009 | 3 | 3.13 | 2.41 | 0.698 | 0.018 |
| 20SU_07.4 | 31.4 | 23.54 | 7.86 | 4.9 | 0.401 | 0.028 | 0.077 | 0.506 | 0.009 | 3 | 3.13 | 2.41 | 0.698 | 0.018 |
| 20SU_07.5 | 31.4 | 23.54 | 7.86 | 4.9 | 0.401 | 0.028 | 0.077 | 0.506 | 0.009 | 3 | 3.13 | 2.41 | 0.698 | 0.018 |
| 20SU_08.1 | 31.6 | 24.28 | 7.95 | 6.1 | 0.419 | 0.028 | 0.085 | 0.532 | 0.009 | 4.8 | 2.88 | 2.09 | 0.75 | 0.02 |
| 20SU_08.2 | 31.6 | 24.28 | 7.95 | 6.1 | 0.419 | 0.028 | 0.085 | 0.532 | 0.009 | 4.8 | 2.88 | 2.09 | 0.75 | 0.02 |
| 20SU_08.3 | 31.6 | 24.28 | 7.95 | 6.1 | 0.419 | 0.028 | 0.085 | 0.532 | 0.009 | 4.8 | 2.88 | 2.09 | 0.75 | 0.02 |
| 20SU_08.4 | 31.6 | 24.28 | 7.95 | 6.1 | 0.419 | 0.028 | 0.085 | 0.532 | 0.009 | 4.8 | 2.88 | 2.09 | 0.75 | 0.02 |
| 20SU_08.5 | 31.6 | 24.28 | 7.95 | 6.1 | 0.419 | 0.028 | 0.085 | 0.532 | 0.009 | 4.8 | 2.88 | 2.09 | 0.75 | 0.02 |
| 20SU_09.1 | 31.1 | 24.16 | 7.99 | 6.4 | 0.203 | 0.025 | 0.034 | 0.262 | 0.01 | 3.2 | 2.73 | 2.43 | 0.38 | 0.024 |
| 20SU_09.2 | 31.1 | 24.16 | 7.99 | 6.4 | 0.203 | 0.025 | 0.034 | 0.262 | 0.01 | 3.2 | 2.73 | 2.43 | 0.38 | 0.024 |
| 20SU_09.3 | 31.1 | 24.16 | 7.99 | 6.4 | 0.203 | 0.025 | 0.034 | 0.262 | 0.01 | 3.2 | 2.73 | 2.43 | 0.38 | 0.024 |
| 20SU_09.4 | 31.1 | 24.16 | 7.99 | 6.4 | 0.203 | 0.025 | 0.034 | 0.262 | 0.01 | 3.2 | 2.73 | 2.43 | 0.38 | 0.024 |
| 20SU_09.5 | 31.1 | 24.16 | 7.99 | 6.4 | 0.203 | 0.025 | 0.034 | 0.262 | 0.01 | 3.2 | 2.73 | 2.43 | 0.38 | 0.024 |
| 20SU_10.1 | 31.1 | 24.21 | 7.88 | 5.3 | 0.388 | 0.036 | 0.071 | 0.495 | 0.008 | 2.9 | 2.23 | 2.28 | 0.595 | 0.014 |
| 20SU_10.2 | 31.1 | 24.21 | 7.88 | 5.3 | 0.388 | 0.036 | 0.071 | 0.495 | 0.008 | 2.9 | 2.23 | 2.28 | 0.595 | 0.014 |
| 20SU_10.3 | 31.1 | 24.21 | 7.88 | 5.3 | 0.388 | 0.036 | 0.071 | 0.495 | 0.008 | 2.9 | 2.23 | 2.28 | 0.595 | 0.014 |
| 20SU_10.4 | 31.1 | 24.21 | 7.88 | 5.3 | 0.388 | 0.036 | 0.071 | 0.495 | 0.008 | 2.9 | 2.23 | 2.28 | 0.595 | 0.014 |
| 20SU_10.5 | 31.1 | 24.21 | 7.88 | 5.3 | 0.388 | 0.036 | 0.071 | 0.495 | 0.008 | 2.9 | 2.23 | 2.28 | 0.595 | 0.014 |
| 20SU_11.1 | 32.1 | 27.38 | 8.03 | 6.8 | 0.112 | 0.018 | 0.021 | 0.151 | 0.007 | 5.9 | 2.16 | 1.68 | 0.238 | 0.016 |
| 20SU_11.2 | 32.1 | 27.38 | 8.03 | 6.8 | 0.112 | 0.018 | 0.021 | 0.151 | 0.007 | 5.9 | 2.16 | 1.68 | 0.238 | 0.016 |
| 20SU_11.3 | 32.1 | 27.38 | 8.03 | 6.8 | 0.112 | 0.018 | 0.021 | 0.151 | 0.007 | 5.9 | 2.16 | 1.68 | 0.238 | 0.016 |
| 20SU_11.4 | 32.1 | 27.38 | 8.03 | 6.8 | 0.112 | 0.018 | 0.021 | 0.151 | 0.007 | 5.9 | 2.16 | 1.68 | 0.238 | 0.016 |
| 20SU_11.5 | 32.1 | 27.38 | 8.03 | 6.8 | 0.112 | 0.018 | 0.021 | 0.151 | 0.007 | 5.9 | 2.16 | 1.68 | 0.238 | 0.016 |
| 20SU_12.1 | 31.3 | 27.35 | 7.96 | 6.2 | 0.118 | 0.026 | 0.021 | 0.165 | 0.007 | 8.2 | 1.99 | 1.66 | 0.296 | 0.016 |
| 20SU_12.2 | 31.3 | 27.35 | 7.96 | 6.2 | 0.118 | 0.026 | 0.021 | 0.165 | 0.007 | 8.2 | 1.99 | 1.66 | 0.296 | 0.016 |
| 20SU_12.3 | 31.3 | 27.35 | 7.96 | 6.2 | 0.118 | 0.026 | 0.021 | 0.165 | 0.007 | 8.2 | 1.99 | 1.66 | 0.296 | 0.016 |
| 20SU_12.4 | 31.3 | 27.35 | 7.96 | 6.2 | 0.118 | 0.026 | 0.021 | 0.165 | 0.007 | 8.2 | 1.99 | 1.66 | 0.296 | 0.016 |
| 20SU_12.5 | 31.3 | 27.35 | 7.96 | 6.2 | 0.118 | 0.026 | 0.021 | 0.165 | 0.007 | 8.2 | 1.99 | 1.66 | 0.296 | 0.016 |
| 20SU_13.1 | 32.3 | 28.28 | 8.08 | 6 | 0.135 | 0.02 | 0.022 | 0.177 | 0.007 | 3.8 | 1.87 | 1.49 | 0.341 | 0.016 |
| 20SU_13.2 | 32.3 | 28.28 | 8.08 | 6 | 0.135 | 0.02 | 0.022 | 0.177 | 0.007 | 3.8 | 1.87 | 1.49 | 0.341 | 0.016 |
| 20SU_13.3 | 32.3 | 28.28 | 8.08 | 6 | 0.135 | 0.02 | 0.022 | 0.177 | 0.007 | 3.8 | 1.87 | 1.49 | 0.341 | 0.016 |
| 20SU_13.4 | 32.3 | 28.28 | 8.08 | 6 | 0.135 | 0.02 | 0.022 | 0.177 | 0.007 | 3.8 | 1.87 | 1.49 | 0.341 | 0.016 |
| 20SU_13.5 | 32.3 | 28.28 | 8.08 | 6 | 0.135 | 0.02 | 0.022 | 0.177 | 0.007 | 3.8 | 1.87 | 1.49 | 0.341 | 0.016 |
| 20SU_14.1 | 31.6 | 27.71 | 7.96 | 6.8 | 0.231 | 0.011 | 0.038 | 0.28 | 0.006 | 3.6 | 1.82 | 1.31 | 0.441 | 0.012 |
| 20SU_14.2 | 31.6 | 27.71 | 7.96 | 6.8 | 0.231 | 0.011 | 0.038 | 0.28 | 0.006 | 3.6 | 1.82 | 1.31 | 0.441 | 0.012 |
| 20SU_14.3 | 31.6 | 27.71 | 7.96 | 6.8 | 0.231 | 0.011 | 0.038 | 0.28 | 0.006 | 3.6 | 1.82 | 1.31 | 0.441 | 0.012 |
| 20SU_14.4 | 31.6 | 27.71 | 7.96 | 6.8 | 0.231 | 0.011 | 0.038 | 0.28 | 0.006 | 3.6 | 1.82 | 1.31 | 0.441 | 0.012 |
| 20SU_14.5 | 31.6 | 27.71 | 7.96 | 6.8 | 0.231 | 0.011 | 0.038 | 0.28 | 0.006 | 3.6 | 1.82 | 1.31 | 0.441 | 0.012 |
| 20SU_15.1 | 31.6 | 28.61 | 7.95 | 6.7 | 0.101 | 0.012 | 0.016 | 0.129 | 0.006 | 7.5 | 1.93 | 1.4 | 0.2 | 0.012 |
| 20SU_15.2 | 31.6 | 28.61 | 7.95 | 6.7 | 0.101 | 0.012 | 0.016 | 0.129 | 0.006 | 7.5 | 1.93 | 1.4 | 0.2 | 0.012 |
| 20SU_15.3 | 31.6 | 28.61 | 7.95 | 6.7 | 0.101 | 0.012 | 0.016 | 0.129 | 0.006 | 7.5 | 1.93 | 1.4 | 0.2 | 0.012 |
| 20SU_15.4 | 31.6 | 28.61 | 7.95 | 6.7 | 0.101 | 0.012 | 0.016 | 0.129 | 0.006 | 7.5 | 1.93 | 1.4 | 0.2 | 0.012 |
| 20SU_15.5 | 31.6 | 28.61 | 7.95 | 6.7 | 0.101 | 0.012 | 0.016 | 0.129 | 0.006 | 7.5 | 1.93 | 1.4 | 0.2 | 0.012 |
| 20SU_16.1 | 31.7 | 27.72 | 8.02 | 6.8 | 0.138 | 0.012 | 0.035 | 0.185 | 0.005 | 3.9 | 1.82 | 1.31 | 0.394 | 0.013 |
| 20SU_16.2 | 31.7 | 27.72 | 8.02 | 6.8 | 0.138 | 0.012 | 0.035 | 0.185 | 0.005 | 3.9 | 1.82 | 1.31 | 0.394 | 0.013 |
| 20SU_16.3 | 31.7 | 27.72 | 8.02 | 6.8 | 0.138 | 0.012 | 0.035 | 0.185 | 0.005 | 3.9 | 1.82 | 1.31 | 0.394 | 0.013 |
| 20SU_16.4 | 31.7 | 27.72 | 8.02 | 6.8 | 0.138 | 0.012 | 0.035 | 0.185 | 0.005 | 3.9 | 1.82 | 1.31 | 0.394 | 0.013 |
| 20SU_16.5 | 31.7 | 27.72 | 8.02 | 6.8 | 0.138 | 0.012 | 0.035 | 0.185 | 0.005 | 3.9 | 1.82 | 1.31 | 0.394 | 0.013 |
| 20WI_01.1 | 12.56 | 25.54 | 8.23 | 8.8 | 0.271 | 0.007 | 0.053 | 0.331 | 0.021 | 3.1 | 6.2 | 2.1 | 0.526 | 0.053 |
| 20WI_01.2 | 12.56 | 25.54 | 8.23 | 8.8 | 0.271 | 0.007 | 0.053 | 0.331 | 0.021 | 3.1 | 6.2 | 2.1 | 0.526 | 0.053 |
| 20WI_01.3 | 12.56 | 25.54 | 8.23 | 8.8 | 0.271 | 0.007 | 0.053 | 0.331 | 0.021 | 3.1 | 6.2 | 2.1 | 0.526 | 0.053 |
| 20WI_01.4 | 12.56 | 25.54 | 8.23 | 8.8 | 0.271 | 0.007 | 0.053 | 0.331 | 0.021 | 3.1 | 6.2 | 2.1 | 0.526 | 0.053 |
| 20WI_01.5 | 12.56 | 25.54 | 8.23 | 8.8 | 0.271 | 0.007 | 0.053 | 0.331 | 0.021 | 3.1 | 6.2 | 2.1 | 0.526 | 0.053 |
| 20WI_02.1 | 12.49 | 25.85 | 8.22 | 8.8 | 0.278 | 0.009 | 0.053 | 0.34 | 0.022 | 3.5 | 5.78 | 1.91 | 0.537 | 0.044 |
| 20WI_02.2 | 12.49 | 25.85 | 8.22 | 8.8 | 0.278 | 0.009 | 0.053 | 0.34 | 0.022 | 3.5 | 5.78 | 1.91 | 0.537 | 0.044 |
| 20WI_02.3 | 12.49 | 25.85 | 8.22 | 8.8 | 0.278 | 0.009 | 0.053 | 0.34 | 0.022 | 3.5 | 5.78 | 1.91 | 0.537 | 0.044 |
| 20WI_02.4 | 12.49 | 25.85 | 8.22 | 8.8 | 0.278 | 0.009 | 0.053 | 0.34 | 0.022 | 3.5 | 5.78 | 1.91 | 0.537 | 0.044 |
| 20WI_02.5 | 12.49 | 25.85 | 8.22 | 8.8 | 0.278 | 0.009 | 0.053 | 0.34 | 0.022 | 3.5 | 5.78 | 1.91 | 0.537 | 0.044 |
| 20WI_03.1 | 12.38 | 26.23 | 8.26 | 8.7 | 0.269 | 0.01 | 0.057 | 0.336 | 0.021 | 5.4 | 5.61 | 1.83 | 0.518 | 0.042 |
| 20WI_03.2 | 12.38 | 26.23 | 8.26 | 8.7 | 0.269 | 0.01 | 0.057 | 0.336 | 0.021 | 5.4 | 5.61 | 1.83 | 0.518 | 0.042 |
| 20WI_03.3 | 12.38 | 26.23 | 8.26 | 8.7 | 0.269 | 0.01 | 0.057 | 0.336 | 0.021 | 5.4 | 5.61 | 1.83 | 0.518 | 0.042 |
| 20WI_03.4 | 12.38 | 26.23 | 8.26 | 8.7 | 0.269 | 0.01 | 0.057 | 0.336 | 0.021 | 5.4 | 5.61 | 1.83 | 0.518 | 0.042 |
| 20WI_03.5 | 12.38 | 26.23 | 8.26 | 8.7 | 0.269 | 0.01 | 0.057 | 0.336 | 0.021 | 5.4 | 5.61 | 1.83 | 0.518 | 0.042 |
| 20WI_04.1 | 12.42 | 26.55 | 8.28 | 8.8 | 0.276 | 0.01 | 0.063 | 0.349 | 0.021 | 5.6 | 5.76 | 1.76 | 0.568 | 0.043 |
| 20WI_04.2 | 12.42 | 26.55 | 8.28 | 8.8 | 0.276 | 0.01 | 0.063 | 0.349 | 0.021 | 5.6 | 5.76 | 1.76 | 0.568 | 0.043 |
| 20WI_04.3 | 12.42 | 26.55 | 8.28 | 8.8 | 0.276 | 0.01 | 0.063 | 0.349 | 0.021 | 5.6 | 5.76 | 1.76 | 0.568 | 0.043 |
| 20WI_04.4 | 12.42 | 26.55 | 8.28 | 8.8 | 0.276 | 0.01 | 0.063 | 0.349 | 0.021 | 5.6 | 5.76 | 1.76 | 0.568 | 0.043 |
| 20WI_04.5 | 12.42 | 26.55 | 8.28 | 8.8 | 0.276 | 0.01 | 0.063 | 0.349 | 0.021 | 5.6 | 5.76 | 1.76 | 0.568 | 0.043 |
| 20WI_05.1 | 12.33 | 26.84 | 8.26 | 8.8 | 0.289 | 0.01 | 0.056 | 0.355 | 0.019 | 6.7 | 6.18 | 1.87 | 0.572 | 0.037 |
| 20WI_05.2 | 12.33 | 26.84 | 8.26 | 8.8 | 0.289 | 0.01 | 0.056 | 0.355 | 0.019 | 6.7 | 6.18 | 1.87 | 0.572 | 0.037 |
| 20WI_05.3 | 12.33 | 26.84 | 8.26 | 8.8 | 0.289 | 0.01 | 0.056 | 0.355 | 0.019 | 6.7 | 6.18 | 1.87 | 0.572 | 0.037 |
| 20WI_05.4 | 12.33 | 26.84 | 8.26 | 8.8 | 0.289 | 0.01 | 0.056 | 0.355 | 0.019 | 6.7 | 6.18 | 1.87 | 0.572 | 0.037 |
| 20WI_05.5 | 12.33 | 26.84 | 8.26 | 8.8 | 0.289 | 0.01 | 0.056 | 0.355 | 0.019 | 6.7 | 6.18 | 1.87 | 0.572 | 0.037 |
| 20WI_06.1 | 12.37 | 29.28 | 8.3 | 8.7 | 0.24 | 0.008 | 0.047 | 0.295 | 0.016 | 6.5 | 6.14 | 1.49 | 0.557 | 0.036 |
| 20WI_06.2 | 12.37 | 29.28 | 8.3 | 8.7 | 0.24 | 0.008 | 0.047 | 0.295 | 0.016 | 6.5 | 6.14 | 1.49 | 0.557 | 0.036 |
| 20WI_06.3 | 12.37 | 29.28 | 8.3 | 8.7 | 0.24 | 0.008 | 0.047 | 0.295 | 0.016 | 6.5 | 6.14 | 1.49 | 0.557 | 0.036 |
| 20WI_06.4 | 12.37 | 29.28 | 8.3 | 8.7 | 0.24 | 0.008 | 0.047 | 0.295 | 0.016 | 6.5 | 6.14 | 1.49 | 0.557 | 0.036 |
| 20WI_06.5 | 12.37 | 29.28 | 8.3 | 8.7 | 0.24 | 0.008 | 0.047 | 0.295 | 0.016 | 6.5 | 6.14 | 1.49 | 0.557 | 0.036 |
| 20WI_07.1 | 12.39 | 29.41 | 8.3 | 8.7 | 0.238 | 0.008 | 0.042 | 0.288 | 0.014 | 6.1 | 6.12 | 1.71 | 0.427 | 0.032 |
| 20WI_07.2 | 12.39 | 29.41 | 8.3 | 8.7 | 0.238 | 0.008 | 0.042 | 0.288 | 0.014 | 6.1 | 6.12 | 1.71 | 0.427 | 0.032 |
| 20WI_07.3 | 12.39 | 29.41 | 8.3 | 8.7 | 0.238 | 0.008 | 0.042 | 0.288 | 0.014 | 6.1 | 6.12 | 1.71 | 0.427 | 0.032 |
| 20WI_07.4 | 12.39 | 29.41 | 8.3 | 8.7 | 0.238 | 0.008 | 0.042 | 0.288 | 0.014 | 6.1 | 6.12 | 1.71 | 0.427 | 0.032 |
| 20WI_07.5 | 12.39 | 29.41 | 8.3 | 8.7 | 0.238 | 0.008 | 0.042 | 0.288 | 0.014 | 6.1 | 6.12 | 1.71 | 0.427 | 0.032 |
| 20WI_08.1 | 13.48 | 28.61 | 8.31 | 8.7 | 0.226 | 0.006 | 0.034 | 0.266 | 0.016 | 4.3 | 6.48 | 1.58 | 0.348 | 0.034 |
| 20WI_08.2 | 13.48 | 28.61 | 8.31 | 8.7 | 0.226 | 0.006 | 0.034 | 0.266 | 0.016 | 4.3 | 6.48 | 1.58 | 0.348 | 0.034 |
| 20WI_08.3 | 13.48 | 28.61 | 8.31 | 8.7 | 0.226 | 0.006 | 0.034 | 0.266 | 0.016 | 4.3 | 6.48 | 1.58 | 0.348 | 0.034 |
| 20WI_08.4 | 13.48 | 28.61 | 8.31 | 8.7 | 0.226 | 0.006 | 0.034 | 0.266 | 0.016 | 4.3 | 6.48 | 1.58 | 0.348 | 0.034 |
| 20WI_08.5 | 13.48 | 28.61 | 8.31 | 8.7 | 0.226 | 0.006 | 0.034 | 0.266 | 0.016 | 4.3 | 6.48 | 1.58 | 0.348 | 0.034 |
| 20WI_09.1 | 13.9 | 30.08 | 8.33 | 8.8 | 0.194 | 0.006 | 0.031 | 0.231 | 0.014 | 4.7 | 5.16 | 1.71 | 0.293 | 0.025 |
| 20WI_09.2 | 13.9 | 30.08 | 8.33 | 8.8 | 0.194 | 0.006 | 0.031 | 0.231 | 0.014 | 4.7 | 5.16 | 1.71 | 0.293 | 0.025 |
| 20WI_09.3 | 13.9 | 30.08 | 8.33 | 8.8 | 0.194 | 0.006 | 0.031 | 0.231 | 0.014 | 4.7 | 5.16 | 1.71 | 0.293 | 0.025 |
| 20WI_09.4 | 13.9 | 30.08 | 8.33 | 8.8 | 0.194 | 0.006 | 0.031 | 0.231 | 0.014 | 4.7 | 5.16 | 1.71 | 0.293 | 0.025 |
| 20WI_09.5 | 13.9 | 30.08 | 8.33 | 8.8 | 0.194 | 0.006 | 0.031 | 0.231 | 0.014 | 4.7 | 5.16 | 1.71 | 0.293 | 0.025 |
| 20WI_10.1 | 12.54 | 30.15 | 8.33 | 8.7 | 0.206 | 0.007 | 0.024 | 0.237 | 0.013 | 5.2 | 5.15 | 1.63 | 0.36 | 0.03 |
| 20WI_10.2 | 12.54 | 30.15 | 8.33 | 8.7 | 0.206 | 0.007 | 0.024 | 0.237 | 0.013 | 5.2 | 5.15 | 1.63 | 0.36 | 0.03 |
| 20WI_10.3 | 12.54 | 30.15 | 8.33 | 8.7 | 0.206 | 0.007 | 0.024 | 0.237 | 0.013 | 5.2 | 5.15 | 1.63 | 0.36 | 0.03 |
| 20WI_10.4 | 12.54 | 30.15 | 8.33 | 8.7 | 0.206 | 0.007 | 0.024 | 0.237 | 0.013 | 5.2 | 5.15 | 1.63 | 0.36 | 0.03 |
| 20WI_10.5 | 12.54 | 30.15 | 8.33 | 8.7 | 0.206 | 0.007 | 0.024 | 0.237 | 0.013 | 5.2 | 5.15 | 1.63 | 0.36 | 0.03 |
| 20WI_11.1 | 13.28 | 32.47 | 8.34 | 8.7 | 0.284 | 0.009 | 0.029 | 0.322 | 0.01 | 5.9 | 4.13 | 1.42 | 0.366 | 0.025 |
| 20WI_11.2 | 13.28 | 32.47 | 8.34 | 8.7 | 0.284 | 0.009 | 0.029 | 0.322 | 0.01 | 5.9 | 4.13 | 1.42 | 0.366 | 0.025 |
| 20WI_11.3 | 13.28 | 32.47 | 8.34 | 8.7 | 0.284 | 0.009 | 0.029 | 0.322 | 0.01 | 5.9 | 4.13 | 1.42 | 0.366 | 0.025 |
| 20WI_11.4 | 13.28 | 32.47 | 8.34 | 8.7 | 0.284 | 0.009 | 0.029 | 0.322 | 0.01 | 5.9 | 4.13 | 1.42 | 0.366 | 0.025 |
| 20WI_11.5 | 13.28 | 32.47 | 8.34 | 8.7 | 0.284 | 0.009 | 0.029 | 0.322 | 0.01 | 5.9 | 4.13 | 1.42 | 0.366 | 0.025 |
| 20WI_12.1 | 13.49 | 33.88 | 8.33 | 8.9 | 0.249 | 0.008 | 0.023 | 0.28 | 0.011 | 5.3 | 4.16 | 1.37 | 0.367 | 0.021 |
| 20WI_12.2 | 13.49 | 33.88 | 8.33 | 8.9 | 0.249 | 0.008 | 0.023 | 0.28 | 0.011 | 5.3 | 4.16 | 1.37 | 0.367 | 0.021 |
| 20WI_12.3 | 13.49 | 33.88 | 8.33 | 8.9 | 0.249 | 0.008 | 0.023 | 0.28 | 0.011 | 5.3 | 4.16 | 1.37 | 0.367 | 0.021 |
| 20WI_12.4 | 13.49 | 33.88 | 8.33 | 8.9 | 0.249 | 0.008 | 0.023 | 0.28 | 0.011 | 5.3 | 4.16 | 1.37 | 0.367 | 0.021 |
| 20WI_12.5 | 13.49 | 33.88 | 8.33 | 8.9 | 0.249 | 0.008 | 0.023 | 0.28 | 0.011 | 5.3 | 4.16 | 1.37 | 0.367 | 0.021 |
| 20WI_13.1 | 12.64 | 33.04 | 8.36 | 8.6 | 0.267 | 0.012 | 0.033 | 0.312 | 0.014 | 4.6 | 5.12 | 0.97 | 0.431 | 0.027 |
| 20WI_13.2 | 12.64 | 33.04 | 8.36 | 8.6 | 0.267 | 0.012 | 0.033 | 0.312 | 0.014 | 4.6 | 5.12 | 0.97 | 0.431 | 0.027 |
| 20WI_13.3 | 12.64 | 33.04 | 8.36 | 8.6 | 0.267 | 0.012 | 0.033 | 0.312 | 0.014 | 4.6 | 5.12 | 0.97 | 0.431 | 0.027 |
| 20WI_13.4 | 12.64 | 33.04 | 8.36 | 8.6 | 0.267 | 0.012 | 0.033 | 0.312 | 0.014 | 4.6 | 5.12 | 0.97 | 0.431 | 0.027 |
| 20WI_13.5 | 12.64 | 33.04 | 8.36 | 8.6 | 0.267 | 0.012 | 0.033 | 0.312 | 0.014 | 4.6 | 5.12 | 0.97 | 0.431 | 0.027 |
| 20WI_14.1 | 13.32 | 33.12 | 8.41 | 8.9 | 0.289 | 0.012 | 0.031 | 0.332 | 0.013 | 4.8 | 4.11 | 0.98 | 0.467 | 0.024 |
| 20WI_14.2 | 13.32 | 33.12 | 8.41 | 8.9 | 0.289 | 0.012 | 0.031 | 0.332 | 0.013 | 4.8 | 4.11 | 0.98 | 0.467 | 0.024 |
| 20WI_14.3 | 13.32 | 33.12 | 8.41 | 8.9 | 0.289 | 0.012 | 0.031 | 0.332 | 0.013 | 4.8 | 4.11 | 0.98 | 0.467 | 0.024 |
| 20WI_14.4 | 13.32 | 33.12 | 8.41 | 8.9 | 0.289 | 0.012 | 0.031 | 0.332 | 0.013 | 4.8 | 4.11 | 0.98 | 0.467 | 0.024 |
| 20WI_14.5 | 13.32 | 33.12 | 8.41 | 8.9 | 0.289 | 0.012 | 0.031 | 0.332 | 0.013 | 4.8 | 4.11 | 0.98 | 0.467 | 0.024 |
| 20WI_15.1 | 13.05 | 32.29 | 8.35 | 8.8 | 0.287 | 0.012 | 0.031 | 0.33 | 0.012 | 4.7 | 4.12 | 1.01 | 0.472 | 0.026 |
| 20WI_15.2 | 13.05 | 32.29 | 8.35 | 8.8 | 0.287 | 0.012 | 0.031 | 0.33 | 0.012 | 4.7 | 4.12 | 1.01 | 0.472 | 0.026 |
| 20WI_15.3 | 13.05 | 32.29 | 8.35 | 8.8 | 0.287 | 0.012 | 0.031 | 0.33 | 0.012 | 4.7 | 4.12 | 1.01 | 0.472 | 0.026 |
| 20WI_15.4 | 13.05 | 32.29 | 8.35 | 8.8 | 0.287 | 0.012 | 0.031 | 0.33 | 0.012 | 4.7 | 4.12 | 1.01 | 0.472 | 0.026 |
| 20WI_15.5 | 13.05 | 32.29 | 8.35 | 8.8 | 0.287 | 0.012 | 0.031 | 0.33 | 0.012 | 4.7 | 4.12 | 1.01 | 0.472 | 0.026 |
| 20WI_16.1 | 13.07 | 32.44 | 8.42 | 8.6 | 0.278 | 0.013 | 0.029 | 0.32 | 0.009 | 4.3 | 4.12 | 1.12 | 0.517 | 0.02 |
| 20WI_16.2 | 13.07 | 32.44 | 8.42 | 8.6 | 0.278 | 0.013 | 0.029 | 0.32 | 0.009 | 4.3 | 4.12 | 1.12 | 0.517 | 0.02 |
| 20WI_16.3 | 13.07 | 32.44 | 8.42 | 8.6 | 0.278 | 0.013 | 0.029 | 0.32 | 0.009 | 4.3 | 4.12 | 1.12 | 0.517 | 0.02 |
| 20WI_16.4 | 13.07 | 32.44 | 8.42 | 8.6 | 0.278 | 0.013 | 0.029 | 0.32 | 0.009 | 4.3 | 4.12 | 1.12 | 0.517 | 0.02 |
| 20WI_16.5 | 13.07 | 32.44 | 8.42 | 8.6 | 0.278 | 0.013 | 0.029 | 0.32 | 0.009 | 4.3 | 4.12 | 1.12 | 0.517 | 0.02 |

**TABLE S2** One-way ANOVA test on variation of each water chemical parameter in different areas.

| Variables | *P*-values | All | | LS | | MS | | HS | |
| --- | --- | --- | --- | --- | --- | --- | --- | --- | --- |
|  |  | Mean | SD | Mean | SD | Mean | SD | Mean | SD |
| Temp | ＞0.05 | 23.535 | 5.849 | 23.781 | 6.238 | 23.182 | 5.780 | 23.623 | 5.669 |
| Salinity | <0.001 | 26.978 | 3.985 | 22.298 | 2.730 | 26.555 | 2.578 | 29.978 | 2.231 |
| pH | ＞0.05 | 8.613 | 0.936 | 8.507 | 0.908 | 8.575 | 0.880 | 8.699 | 0.983 |
| DO | <0.001 | 7.009 | 0.957 | 6.694 | 1.050 | 6.961 | 1.011 | 7.224 | 0.799 |
| NO_3_^-^-N | <0.001 | 0.326 | 0.165 | 0.486 | 0.191 | 0.334 | 0.089 | 0.228 | 0.100 |
| NO_2_^-^-N | <0.001 | 0.023 | 0.032 | 0.040 | 0.051 | 0.024 | 0.027 | 0.013 | 0.007 |
| NH_4_^+^-N | <0.001 | 0.060 | 0.032 | 0.075 | 0.030 | 0.061 | 0.019 | 0.051 | 0.036 |
| DIN | <0.001 | 0.409 | 0.196 | 0.601 | 0.224 | 0.419 | 0.113 | 0.291 | 0.116 |
| DIP | <0.001 | 0.017 | 0.008 | 0.022 | 0.008 | 0.016 | 0.007 | 0.014 | 0.008 |
| Chl-*a* | ＞0.05 | 4.172 | 1.888 | 4.206 | 2.417 | 4.033 | 1.570 | 4.244 | 1.724 |
| TOC | <0.001 | 3.324 | 1.405 | 4.006 | 1.057 | 3.428 | 1.358 | 2.861 | 1.442 |
| COD | <0.001 | 1.891 | 0.523 | 2.411 | 0.323 | 2.086 | 0.312 | 1.460 | 0.345 |
| TN | <0.001 | 0.618 | 0.301 | 0.926 | 0.367 | 0.610 | 0.164 | 0.444 | 0.144 |
| TP | <0.001 | 0.037 | 0.020 | 0.048 | 0.021 | 0.036 | 0.017 | 0.031 | 0.017 |
| C:N | <0.001 | 7.568 | 4.710 | 6.186 | 3.672 | 7.486 | 4.910 | 8.399 | 5.051 |
| C:P | <0.001 | 311.122 | 250.936 | 237.955 | 77.538 | 299.375 | 165.264 | 382.816 | 372.943 |
| N:P | ＞0.05 | 45.534 | 25.204 | 48.570 | 24.300 | 44.707 | 21.886 | 46.724 | 29.392 |

**TABLE S3** One-way ANOVA test on variation of each water chemical parameter in eight seasons

| Variables | *P*-values | 18SP | | 18SU | | 18Au | | 18WI | | 20SP | | 20SU | | 20Au | | 20WI | |
| --- | --- | --- | --- | --- | --- | --- | --- | --- | --- | --- | --- | --- | --- | --- | --- | --- | --- |
|  |  | Mean | SD | Mean | SD | Mean | SD | Mean | SD | Mean | SD | Mean | SD | Mean | SD | Mean | SD |
| Temperature | <0.001 | 24.968 | 0.692 | 29.459 | 0.188 | 27.413 | 2.364 | 19.067 | 0.563 | 21.244 | 0.490 | 31.719 | 0.384 | 22.067 | 0.232 | 12.857 | 0.492 |
| Salinity | <0.001 | 26.171 | 3.213 | 24.811 | 4.943 | 26.486 | 4.881 | 27.673 | 2.149 | 28.524 | 3.561 | 24.114 | 3.377 | 28.169 | 2.423 | 29.736 | 2.809 |
| pH | <0.001 | 10.045 | 0.501 | 10.317 | 0.633 | 7.954 | 0.133 | 8.219 | 0.216 | 8.073 | 0.072 | 7.908 | 0.095 | 8.294 | 0.080 | 8.314 | 0.055 |
| DO | <0.001 | 6.793 | 0.349 | 5.973 | 0.457 | 6.907 | 0.177 | 6.633 | 0.464 | 7.435 | 0.131 | 5.900 | 0.649 | 7.563 | 0.331 | 8.750 | 0.087 |
| NO_3_^-^-N | <0.001 | 0.342 | 0.231 | 0.347 | 0.184 | 0.372 | 0.165 | 0.331 | 0.098 | 0.432 | 0.181 | 0.302 | 0.156 | 0.223 | 0.063 | 0.259 | 0.029 |
| NO_2_^-^-N | <0.001 | 0.009 | 0.004 | 0.071 | 0.067 | 0.027 | 0.024 | 0.008 | 0.002 | 0.027 | 0.009 | 0.025 | 0.008 | 0.010 | 0.002 | 0.009 | 0.002 |
| NH_4_^+^-N | <0.001 | 0.050 | 0.026 | 0.063 | 0.014 | 0.072 | 0.021 | 0.113 | 0.034 | 0.045 | 0.020 | 0.057 | 0.029 | 0.046 | 0.020 | 0.040 | 0.013 |
| DIN | <0.001 | 0.401 | 0.259 | 0.480 | 0.254 | 0.471 | 0.199 | 0.452 | 0.096 | 0.503 | 0.189 | 0.383 | 0.191 | 0.278 | 0.077 | 0.308 | 0.037 |
| DIP | <0.001 | 0.021 | 0.008 | 0.028 | 0.005 | 0.022 | 0.009 | 0.015 | 0.002 | 0.010 | 0.005 | 0.012 | 0.006 | 0.013 | 0.008 | 0.015 | 0.004 |
| Chl-*a* | <0.001 | 6.221 | 2.231 | 4.073 | 0.973 | 2.420 | 0.729 | 5.080 | 1.387 | 1.870 | 0.823 | 4.163 | 1.724 | 4.713 | 1.231 | 5.044 | 0.968 |
| TOC | <0.001 | 2.502 | 0.484 | 2.715 | 0.701 | 2.765 | 0.636 | 2.512 | 0.422 | 2.384 | 0.512 | 2.801 | 0.872 | 5.401 | 1.043 | 5.271 | 0.860 |
| COD | <0.001 | 2.109 | 0.547 | 2.065 | 0.453 | 1.879 | 0.484 | 1.493 | 0.438 | 1.975 | 0.513 | 2.067 | 0.498 | 2.013 | 0.456 | 1.529 | 0.345 |
| TN | <0.001 | 0.651 | 0.494 | 0.638 | 0.269 | 0.794 | 0.307 | 0.663 | 0.183 | 0.755 | 0.322 | 0.525 | 0.224 | 0.464 | 0.090 | 0.458 | 0.087 |
| TP | <0.001 | 0.046 | 0.015 | 0.060 | 0.015 | 0.050 | 0.024 | 0.032 | 0.005 | 0.023 | 0.010 | 0.025 | 0.012 | 0.029 | 0.021 | 0.032 | 0.009 |
| C:N | <0.001 | 6.373 | 2.757 | 5.603 | 1.954 | 4.475 | 1.361 | 4.540 | 0.528 | 4.013 | 1.143 | 6.833 | 2.203 | 14.210 | 4.592 | 13.865 | 3.360 |
| C:P | <0.001 | 159.629 | 73.397 | 127.583 | 54.077 | 158.196 | 45.406 | 210.443 | 52.276 | 296.245 | 65.554 | 324.088 | 73.844 | 732.386 | 424.486 | 435.438 | 69.877 |
| N:P | <0.001 | 36.872 | 30.321 | 24.918 | 13.348 | 39.270 | 17.426 | 47.752 | 16.608 | 78.845 | 23.713 | 52.402 | 21.619 | 48.796 | 20.731 | 32.810 | 8.631 |

**TABLE S4** Spearman correlations between the harmful microalgae community alpha diversity (Shannon) and environmental factors.

| Variables | Alpha diversity | | |
| --- | --- | --- | --- |
|  | LS | MS | HS |
| Temperature | 0.05 | -0.04 | 0 |
| Salinity | -0.13 | 0.13 | 0.01 |
| pH | 0.13 | 0.11 | 0.49 *** |
| DO | -0.19 * | -0.1 | -0.17 ** |
| NO_3_^-^-N | 0.4 *** | -0.01 | -0.16 ** |
| NO_2_^-^-N | -0.09 | -0.28 *** | -0.34 *** |
| NH_4_^+^-N | 0.19 * | 0.32 *** | 0.11 |
| DIN | 0.34 *** | 0.05 | -0.09 |
| DIP | -0.1 | 0.35 *** | 0.52 *** |
| Chl-*a* | 0.24 ** | 0.12 | 0.15 * |
| TOC | -0.35 *** | -0.18 * | -0.15 * |
| COD | 0.22 ** | -0.18 * | -0.21 *** |
| TN | 0.46 *** | 0.05 | -0.19 ** |
| TP | -0.07 | 0.41 *** | 0.51 *** |
| C:N | -0.51 *** | -0.19 * | -0.05 |
| C:P | -0.28 *** | -0.39 *** | -0.49 *** |
| N:P | 0.35 *** | -0.23 ** | -0.5 *** |

The number is the *r* values. The asterisk indicates the *p*-value; *, *p* < 0.05, pale green; **, *p* < 0.01, yellow; ***, *p* < 0.001, pale red.
